# “Bind, cleave and leave”: multiple turnover catalysis of RNA cleavage by bulge–loop inducing supramolecular conjugates

**DOI:** 10.1093/nar/gkab1273

**Published:** 2021-12-30

**Authors:** Bahareh Amirloo, Yaroslav Staroseletz, Sameen Yousaf, David J Clarke, Tom Brown, Harmesh Aojula, Marina A Zenkova, Elena V Bichenkova

**Affiliations:** School of Health Sciences, Faculty of Biology, Medicine and Health, University of Manchester, Oxford Road, Manchester M13 9PT, UK; Institute of Chemical Biology and Fundamental Medicine SB RAS, 8 Laurentiev Avenue, 630090 Novosibirsk, Russian Federation; School of Health Sciences, Faculty of Biology, Medicine and Health, University of Manchester, Oxford Road, Manchester M13 9PT, UK; School of Health Sciences, Faculty of Biology, Medicine and Health, University of Manchester, Oxford Road, Manchester M13 9PT, UK; Department of Chemistry, Chemistry Research Laboratory, University of Oxford, 12 Mansfield Road, Oxford OX1 3TA, UK; School of Health Sciences, Faculty of Biology, Medicine and Health, University of Manchester, Oxford Road, Manchester M13 9PT, UK; Institute of Chemical Biology and Fundamental Medicine SB RAS, 8 Laurentiev Avenue, 630090 Novosibirsk, Russian Federation; School of Health Sciences, Faculty of Biology, Medicine and Health, University of Manchester, Oxford Road, Manchester M13 9PT, UK

## Abstract

Antisense sequence-specific knockdown of pathogenic RNA offers opportunities to find new solutions for therapeutic treatments. However, to gain a desired therapeutic effect, the multiple turnover catalysis is critical to inactivate many copies of emerging RNA sequences, which is difficult to achieve without sacrificing the sequence-specificity of cleavage. Here, engineering two or three catalytic peptides into the bulge–loop inducing molecular framework of antisense oligonucleotides achieved catalytic turnover of targeted RNA. Different supramolecular configurations revealed that cleavage of the RNA backbone upon sequence-specific hybridization with the catalyst accelerated with increase in the number of catalytic guanidinium groups, with almost complete demolition of target RNA in 24 h. Multiple sequence-specific cuts at different locations within and around the bulge–loop facilitated release of the catalyst for subsequent attacks of at least 10 further RNA substrate copies, such that delivery of only a few catalytic molecules could be sufficient to maintain knockdown of typical RNA copy numbers. We have developed fluorescent assay and kinetic simulation tools to characterise how the limited availability of different targets and catalysts had restrained catalytic reaction progress considerably, and to inform how to accelerate the catalytic destruction of shorter linear and larger RNAs even further.

## Graphical Abstract

**Graphical Abstract:**
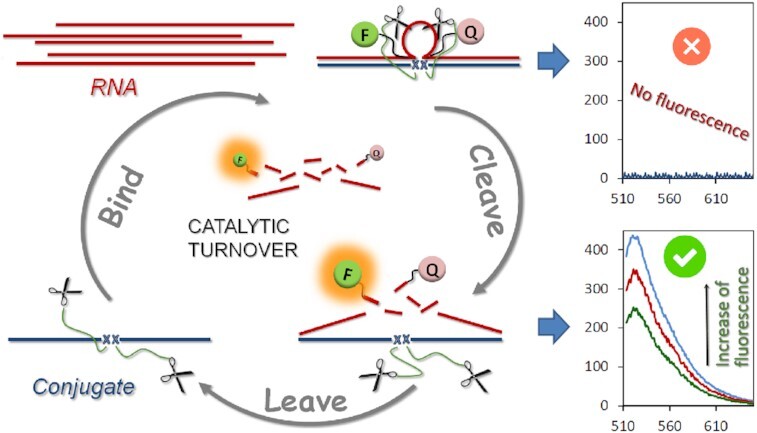
Multiple substrate turnover in RNA cleavage is catalysed by *‘smart’* conjugate bearing several cleaving groups to attack the induced bulge–loops from different directions. Multiple cuts in the target region cause the reduction in size of the RNA cleavage products, thus facilitating collapse of the *RNA-conjugate* complex and release of the conjugate, which then becomes available for the next attack on target RNA. The destruction of multiple RNA copies through reaction catalytic turnover is monitored here by fluorescence.

## INTRODUCTION

RNA plays a pivotal role in transforming biology from health to disease due to its involvement in many biological pathways, including transcriptional and translational regulation (1), epigenetic memory (2), RNA splicing (3) and retroviral replication (4). The importance of RNAs in initiation and development of pathophysiology in humans (5) (cancer (6–8), inflammation (9), neurodegeneration (10,11) or persistent intracellular infections (12)) has sparked rapidly growing interest in sequence-specific targeting of disease-relevant RNAs (*e.g*. messenger RNA transcripts encoding pathogenic proteins, non-coding RNAs involved in cellular signalling pathways, or viral genomic RNAs).

Knockdown of gene expression by RNA silencing is widely used to study complex biological processes (13,14) including disease origin, initiation and progression. Success here is leading to a new class of therapeutic interventions (15–17) to confront diseases ‘*upstream*’ by targeting their genomic origin, with precise molecular destruction of pathogenic RNAs before they translate into the disease. Over recent decades, powerful and versatile sequence-specific interventions, such as siRNAs (18–24) CRISPR (25–27) and antisense oligonucleotides (ASOs) (28–41) have emerged for genome editing and for manipulation of gene expression, reliant upon the precision of Watson-Crick base-pairing between short RNA or DNA guide sequences and target nucleic acids. Recent advances have enhanced their serum stability, safety profiles and improved cell-targeting capabilities (22–26,31). Successful oligonucleotide therapeutics include three siRNA drugs recently approved by FDA (patisiran (28), givosiran (29) and lumasiran (30)) and further seven candidates in Phase 3 clinical trials (31). Eleven antisense oligonucleotide-based therapeutics have broken into the pharmaceutical market (32–42), with many others (>100) in advanced stages of the drug development pipeline. All rely on the recruitment of intracellular enzyme complexes (Cas proteins for CRISPR, RISC complexes for siRNA and RNase H for ASOs) and other endogenous cofactors, whose levels fluctuate within and between cell types. The biological cascade triggered by siRNA is complex; it can initiate the activation of the innate immune response (19) and lead to off-target effects (18). Saturation of cellular machinery and the subsequent interference with miRNA-mediated gene regulation has been raised for siRNA (42). CRISPR was developed for DNA editing and is only recently reported for RNA editing (27,43), albeit with off-target effects at this stage (44). More developed and simpler ASO-mediated mRNA knockdown relies upon the rate-limiting availability of RNase H1 in the cytosol and nucleus, which varies within and between different cell and tissue types (45), and may help explain variable therapeutic responses (46,47). Artificial enzymes may avoid reliance on intracellular enzyme complexes. Chemically-engineered artificial ribonucleases with metal-dependent (Zn(II), Cu(II), Dy(III), Eu(III)) cleaving groups demonstrated an enzyme-like activity with reaction turnover in RNA cleavage (48–52), but remained susceptible to uncontrollable metal loss, which may lead to degradation of non-target biopolymers (53,54) and trigger cytotoxicity. Biologically-stable derivatives of PNA oligomers, PNAzymes, incorporating either Cu^2+^-2,9-dimethylphenanthroline or tris(2-aminobenzimidazole) RNA-cleaving constructs also showed multiple substrate turnover against model synthetic RNA (55–57), but were never tested against clinically-relevant biological RNAs.

Recently-discovered metal-free peptidyl-oligonucleotide conjugates (58–66) offer an alternative to post-transcriptional gene silencing because they cleave functional and regulatory RNA sequences without reliance on these additional enzymes, endogenous cofactors or exogenous species (e.g. metal ions). Chemical conjugation of catalytic peptides and RNA recognition motifs (antisense oligonucleotides) within the same molecular scaffold produces self-contained silencing molecules, which recognize, bind and destroy the RNA target without the necessity to rely on the larger complexes with ‘external’ guides and enzyme-mediated cleavage. The catalytic activity of these peptidyl-oligonucleotide conjugates is provided by short amphiphilic peptides (59–63,65,66), which contain alternating basic (arginine) and hydrophobic (leucine) amino acids. Arginine guanidinium groups are the key players in catalysis (61,62), which appear to act as proton shuttles (67) through various tautomeric forms, by facilitating the proton transfer between the attacking 2′-OH, non-bridging phosphate oxygen and departing 5′-hydroxyalkyl group. The merger of the recognition and catalytic functionalities into the same structure evades the complexities inherent in existing gene-editing and RNA silencing approaches, which require Cas9 (for CRISPR), RISC (for siRNA), RNase H (for antisense) endogenous cofactors and/or metal ions. Crucially, irreversible cleavage of pathological and regulatory RNAs allow cellular pathways to be switched from ‘*diseased*’ to ‘*normal*’ to achieve therapeutic effect. In this context our pilot structures (58–60,64) have progressed into such biologically-active conjugates (61–63,65–66) capable of inhibiting malignant growth in tumour models (63,66). Recently, we have shown that ‘hairpin’ peptidyl-oligonucleotide conjugates could selectively demolish highly-oncogenic microRNA, trigger apoptosis in cancer cells, inhibit cell invasiveness and suppress tumour proliferation *in vitro* (63,66). A single treatment of tumour cells with such catalytic conjugates prior to transplantation into mice knocked-out their malignant properties and continued to inhibit subsequent tumour growth *in vivo* (66).

Hitherto, our peptidyl-oligonucleotide conjugates showed the ability to cleave RNA at two different extremes, either: (1) sequence-specifically (61–63,65–66,68) by site-directed cleavage of the regions adjacent to the binding sites, but without catalytic turnover; or (2) non-specifically at non-complementary regions, distant from the major RNA binding region, but with a high level of catalytic turnover (58–60,64). Precise sequence-specific recognition of the target, which is essential to avoid toxicity, opposes the ability of the conjugate to leave RNA after each cleavage event and achieve the catalytic turnover necessary for a potent silencing without reliance on variable cellular machinery. Sequence-specificity has been at the cost of potency, thus elevating potential dosages, side effects and costs. Combining these mutually-opposing extremes into a single molecule is the challenge addressed here.

We recently developed a series of bulge–loop inducing peptidyl-oligonucleotide conjugates bearing a single stretch of catalytic peptide acetyl-[LRLRG]_2_-CO_2_H attached at the central point of the recognition motif, which hybridized with target RNA (tRNA^Phe^) in a sequence-specific manner and cut it at multiple positions (69). A short region (2nt–5nt) in the middle of the recognition motif that was not complementary to the target RNA sequence served two purposes: (a) it forced the RNA to adopt a bulge–loop (B–L) structure upon hybridization and (b) it allowed the catalytic peptide to be juxtaposed against the induced RNA bulge–loop to catalyse cleavage of the exposed single-stranded region. Flexible attachment of the catalytic peptide *via* an internal abasic nucleotide, whether in α- or β-configuration, promoted effective cleavage of the target tRNA^Phe^ at multiple positions of the induced bulge–loop, and at closely-located folds, with up to 90% demolition of tRNA^Phe^ in 48 h (69).

**Figure 1.**
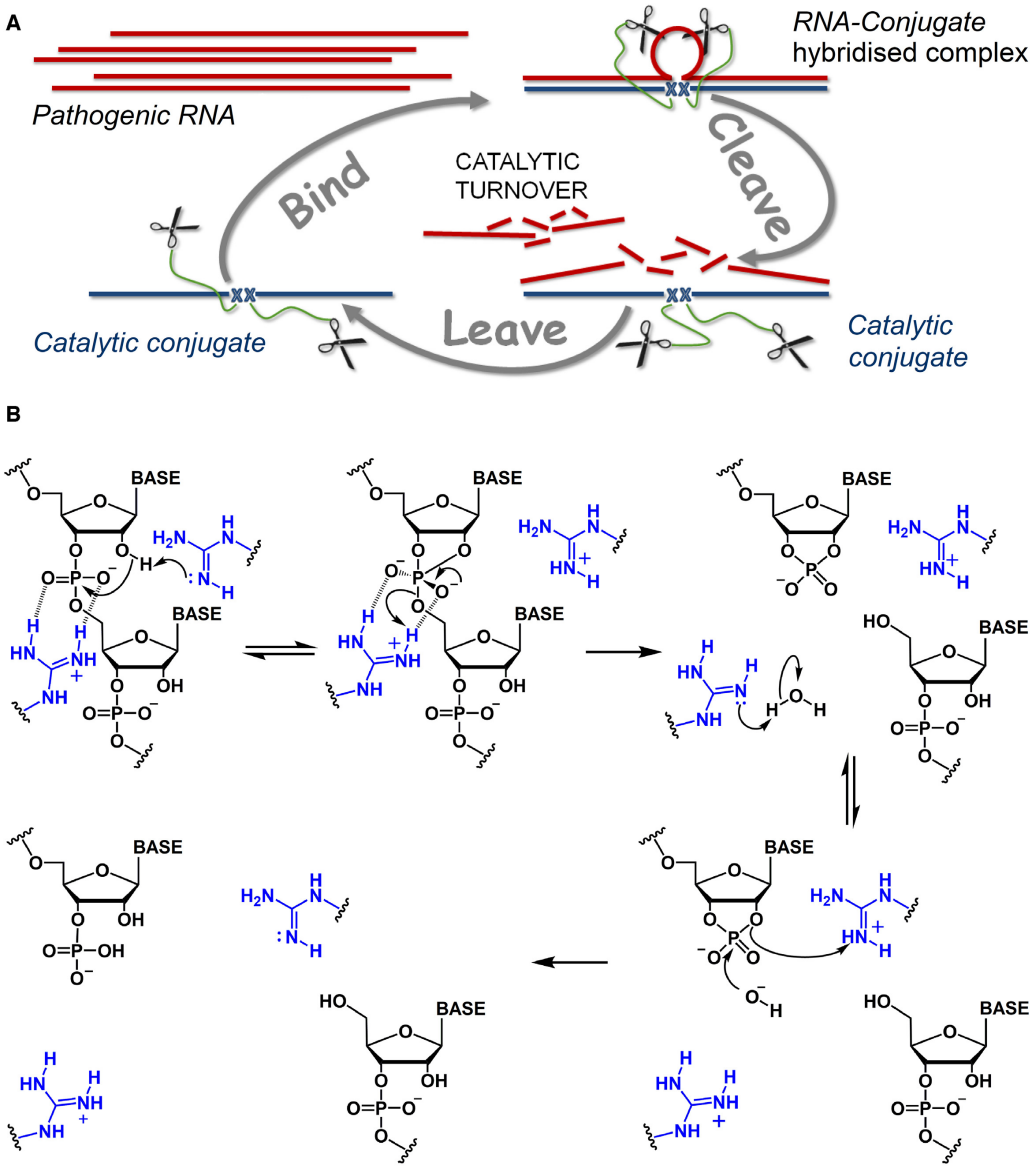
(**A**) Postulated cycle of ‘smart’ catalytic conjugate cleavage of multiple copies of the target RNA molecule. Multiple reaction turnover of RNA substrates requires a rapid release of the cleaved fragments after each cleavage event to liberate catalytic conjugates from the hybridized complexes and allow them to attack the next RNA target. (**B**) Hypothetical mechanism of phosphodiester bond cleavage catalysed by the arginine guanidinium groups (blue) of the amphiphilic peptide. The synchronized action of two guanidinium groups, through formation of a *guanidine-guanidinium* dyad, facilitates the proton transfer between the attacking 2′-OH, non-bridging phosphate oxygen and departing 5′-O group (adapted from (69,70)). A neutral guanidine group (acting as a general base) deprotonates the 2’-OH group of RNA ribose, leading to the formation of a di-anionic pentaoxyphosphorane intermediate, while the protonated (positively-charged) guanidinium moiety coordinates the negatively-charged phosphate group through electrostatic interaction, and acts as an electrophilic activator, thus facilitating the transesterification reaction.

Here, we achieve catalytic turnover in RNA cleavage through incorporation of several catalytic peptides into the same molecular scaffold (see Figure 1A). Earlier postulated RNA backbone transesterification can be catalysed by the synchronized catalytic action of two guanidinium groups present in the same molecular scaffold (70), through the formation of *guanidine-guanidinium* dyad (Figure 1B). By raising the number of catalytic arginine residues within the bulged region of single-stranded RNA, the probability of forming *guanidine-guanidinium* dyads catalysing multiple cuts in the target region was increased. The consequent reduction in size of the RNA cleavage products facilitated collapse of the *RNA-conjugate* complex and released the catalyst to become available for the next attack on target RNA. This allowed us to achieve the crucial catalytic ‘*bind, cleave and leave*’ cycle of a ribonuclease enzyme, but with precise sequence-specificity that natural ribonucleases lack.

We report a range of ‘*bis*’ and ‘*triple*’ bulge–loop inducing conjugates, which carry two or three catalytic peptides attached through the internal abasic sugar residues *via* anomeric C1’ carbon either in αα, ββ, βα, αβ or βββ-configuration. The ‘*bis*’ and ‘*triple*’ catalytic activities were compared with the ‘*single*’ conjugates reported earlier (69) both against larger-structured tRNA^Phe^ and short linear RNA targets, which allowed kinetic analysis to characterize how catalytic turnover is achieved in a sequence-specific manner.

## MATERIALS AND METHODS

### Chemicals, reagents, equipment and facilities

The peptide Ac-Leu-Arg-Leu-Arg-Gly-Leu-Arg-Leu-Arg-Gly-OH (Ac-[LRLRG]_2_-CO_2_H) was purchased from Biomatik (Cambridge, Ontario, Canada). Modified oligonucleotide sequences containing two or more internal abasic nucleotides, with aminohexyl linker attached at C1’ position in α or β-configuration were purchased from ATDBio Ltd (Southampton, UK). Alpha and beta aminohexyl phosphoramidite monomers were purchased from Link Technologies Ltd (Scotland, UK). Fluorescently-labelled, linear perfect-match target **F**-**Q**-RNA (^5’^GTCCTGTGTTCGA**T^F^**C**C_r_A_r_C_r_A_r_G_r_**AA**T^Q^**TCGCACCA^3’^, where **F** is fluorescein and **Q** is dabcyl quencher) was purchased from ATDBio Ltd (Southampton, UK). A scrambled conjugate (5’-luc-h-9/1: ^5’^CAAGTCTCGTATGTGTCAGCGAAAGCTGAC^3’^, with the underlined region representing the hairpin) was synthesized following the previously reported protocol (63) and was used here as a control for sequence-specificity of RNA cleavage. The unconjugated oligonucleotide for the scrambled conjugate was acquired from the Institute of Chemical Biology and Fundamental Medicine (Novosibirsk, Russia).

All chemicals and solvents were purchased from Sigma-Aldrich (UK) unless stated otherwise. Water was purified by a Milli-Q purification system (Millipore, USA). Reversed-phase HPLC purification of synthesized peptide and conjugates was carried out on PerkinElmer (Series 200, ACE 10 C4 250 × 21.2 mm, MA, USA) and Agilent 1100 (Agilent Technologies, Santa Clara, CA) HPLC systems equipped with diode array detector and Rheodyne (3725i) manual injector on a semi-preparative Phenomenex Luna C-18 column (5 μm, 10 × 250 mm, 100 A, Phenomenex; CA, USA). NAP disposable columns prepacked with Sephadex G-25 DNA grade (GE Healthcare Life Sciences) were used to desalt peptidyl-oligonucleotide conjugates. Oligonucleotide concentrations were calculated using extinction coefficients (see Supplementary Table S1) for absorbance at a wavelength of 260 nm, recorded on a Varian Cary 4000 dual beam UV-Vis spectrophotometer (Australia). ^1^H NMR spectra were recorded using Bruker Avance II+ spectrometers operating at proton frequencies of 400 MHz using BBI ^1^H/D-BB Z-GRD Z8202/0347 probe. Bruker Topspin 4.0.3 software was used for data processing. MALDI (matrix-assisted laser desorption ionization) mass spectra were collected on a Bruker Daltonics Ultraflex TOF/TOF mass spectrometer (MA, USA) at the Manchester Interdisciplinary Biocentre, University of Manchester.

The analytical data and full characterization of the generated peptides and peptidyl-oligonucleotide conjugates are given in the Supplementary Information (see Supplementary Figures S1–S9).

### Peptide synthesis, purification and characterization

Ac-Leu-Arg-Leu-Arg-Leu-Arg-Gly (Ac-[LR]_3_G) was synthesized manually using solid-phase peptide synthesis on Boc-Gly-PAM resin (211 mg, 0.148 mmol). The Boc protective group was removed by the addition (2 × 15 ml for 2 and 25 min) of 50% (v/v) trifluoroacetic acid in dichloromethane (1:1, TFA: DCM). The resin was subsequently washed with DCM (4 × 15 ml) and neutralized with 5% DIPEA in DCM. Before each coupling reaction, the resin was washed with DCM (3 × 15 ml) and DMF (1 × 15 ml). The amino acid coupling reactions were achieved by pre-activating either Boc-Arg(Tos)-OH (193 mg, 0.45 mmol) or Boc-Leu-OH (104 mg, 0.45 mmol) in DMF (10 ml) with DIPEA (174 mg, 1.35 mmol) and HBTU (171 mg, 0.45 mmol). The coupling reaction proceeded for 45 min at room temperature under mechanical shaking. Following synthesis, the N-terminus was acetylated through the addition of acetic anhydride (1.80 mmol) with DIPEA (1.8 mmol) in DMF (15 mL). The peptide was cleaved from the resin by treatment with HF in the presence of *p*-cresol (0.75 g) and thiocresol (0.25 g) scavenger mixture for 3 h in an ice bath under constant stirring. After removal of HF, the crude peptide was precipitated and washed with cold diethyl ether (2 × 30 ml). Finally, the peptide was extracted with 10% acetic acid and 80% acetonitrile/0.1% TFA and then freeze dried.

The synthesized crude peptide was dissolved in 30% (v/v) acetic acid and purified by reverse-phase (RP) HPLC (ACE 10 C4 250 × 21.2 mm, PerkinElmer Series 200, MA US) using 0.1% TFA in HPLC-grade water (eluent A) and 0.1% TFA in acetonitrile (eluent B) with a linear gradient (0–100%) over 45 min at a flow rate of 8 ml/min. Following purification, the peptide was characterized using ^1^H NMR spectroscopy (Bruker Avance II + NMR spectrometer, operating at proton frequencies of 400 MHz) and mass spectrometry (MALDI-ToF/ToF, spectra collected on Bruker Daltonics Ultraflex II mass spectrometer (MA, USA)). Analytical data for the Acetyl-[LRLRG]_2_-CO_2_H peptide has been previously reported in (69); MALDI-TOF and ^1^H NMR spectra can be seen in Supplementary Information (see Supplementary Figures S1 and S2).

#### Acetyl-[LR]_3_G-CO_2_H

MALDI-TOF-MS: *m/z* = 926 [M+H]^+^, (MW = 925 calcd. for [C_40_H_76_N_16_O_9_]) (Supplementary Figure S3). ^1^H NMR (Supplementary Figure S4) (400 MHz, D_2_O, 25°C, 0.35 mM TSP): δ 0.86–0.94 (m, 18H, Leu-H^δ^), 1.49–1.94 (m, 21H, 6 × Arg-H^β^, 6 × Arg-H^γ^, 6 × Leu-H^β^, 3 × Leu-H^γ^), 2.02 (s, 3H, CH_3_), 3.21 (m, 6H, Arg-H^δ^), 3.84–4.37 (m, 8H, 2 × Gly-H, 6× Leu/Arg-H^α^).

### Conjugate synthesis

The water-soluble lithium salt of the oligonucleotide containing two or three abasic nucleotides was converted to the cetyltrimethylammonium (CTAB) salt (50 nmol) soluble in DMSO by dropwise addition of 4% (w/v) CTAB as previously described (69). This earlier protocol was amended to accommodate the higher excess of peptide over oligonucleotide, where dried peptide dissolved in DMSO (100-fold excess, 5 μmol) was pre-activated using 4-dimethylaminopyridine (DMAP) and N, N'-dicyclohexylcarbodiimide (DCC) in 1.5-fold excess over peptide each (7.5 μmol each) at 60°C for 24 h (see Supplementary Figure S5 for synthetic route). The conjugate along with unreacted starting oligonucleotide were precipitated in 4% (w/v) LiClO_4_ in acetone (1.8 ml) for 36 h as described (69), then excess reagent was removed by size-exclusion chromatography. In instances where different amounts of the oligonucleotide were used, the concentration of the peptide, DMAP and DCC were re-scaled accordingly to maintain the same molar ratios.

### Conjugate purification and characterization

Crude conjugates were purified using RP-HPLC with a 0.05 M LiClO_4_ in water as eluent A and 0.05 M LiClO_4_ in acetonitrile as eluent B with 2.0 ml/min flow rate of 0% B for 3 min, and 0–100% B over 30 min with absorbance monitored at 260 nm. The identity and purity of conjugates were confirmed by RP-HPLC, ^1^H NMR spectroscopy and mass spectrometry.

#### BC5-L-ββ

RP-HPLC fraction at around 28 min was collected and lyophilized to give a white powder (17 nmol, 34%) (Supplementary Figure S6). Excess salt was removed by size-exclusion chromatography using NAP disposable columns. MALDI-MS: *m/z* = 11189 [M+Na]^+^ (MW = 11166 calcd. For [C_385_H_563_N_151_O_187_P_28_] (Supplementary Figure S7).^1^H NMR (Supplementary Figures S8 and S9) (D_2_O with TSP (0.01 mM), 400 MHz): δ 0.79–0.86 (m, 48H, Leu-H^δ^), 1.16–2.22 (m, 147H, 28× H2’ and 28× H2’’ sugar ring protons, 5× CH_3_ of 5× dT, 16× Arg-H^β^, 16× Arg-H^γ^, 16× Leu-H^β^, 8× Leu-H^γ^, 12× CH_2_ (two aminohexyl linker), 2× acetyl-CH_3_), 3.16 (m, 16H, 16 × Arg-H^δ^), 3.66–4.41 (m,108H, 84 × H4’/H5’H5’ sugar ring protons, 4 × Gly-CH_2_, 16 × Leu/Arg-H^α^), 4.99–6.27 (m, 33H, 28 × H1’ sugar ring protons, 5 × H5 of dC), 7.23–8.47 (m, 34H, 34 × Ar-H from dG(H8 × 8), dA(H8 × 8), dA(H2 × 8), dC(H6 × 5) and dT(H6 × 5)). H3’ sugar ring protons (3.6–4.4 ppm) were not analysed due to suppression of the residual water signal at 4.10 ppm.

#### BC5-L-αα

RP-HPLC fraction at around 28 min was collected and lyophilized to give a white powder (16 nmol, 32%) (Supplementary Figure S6). Excess salt was removed by size-exclusion chromatography using NAP disposable columns. MALDI-MS: *m/z* = 11190 [M+Na+H]^+^ (MW = 11166 calcd. For [C_385_H_563_N_151_O_187_P_28_]) (Supplementary Figure S7).^1^H NMR (Supplementary Figure S8) (D_2_O with TSP (0.01 mM), 400 MHz): δ 0.78–0.85 (m, 48H, Leu-H^δ^), 1.15–2.43 (m, 147H, 28 × H2’ and 28 × H2’’ sugar ring protons, 5× CH_3_ of 5× dT, 16× Arg-H^β^, 16× Arg-H^γ^, 16× Leu-H^β^, 8× Leu-H^γ^, 12× CH_2_ (two aminohexyl linker), 2× acetyl-CH_3_), 3.17 (m, 16H, 16 × Arg-H^δ^), 3.57–4.35 (m, 108H, 84× H4’/H5’H5’ sugar ring protons, 4× Gly-CH_2_, 16× Leu/Arg-H^α^), 4.96–6.25 (m, 33H, 28 × H1’ sugar ring protons, 5 × H5 of dC), 7.25–8.46 (m, 34H, 34 × Ar-H from dG(H8 × 8), dA(H8 × 8), dA(H2 × 8), dC(H6 × 5) and dT(H6 × 5)). H3’ sugar ring protons (3.6–4.4 ppm) were not analysed due to suppression of the residual water signal at 4.12 ppm.

#### BC5-L-αβ

RP-HPLC fraction at around 28 min was collected and lyophilized to give a white powder (14 nmol, 28%) (Supplementary Figure S6). Excess salt was removed by size-exclusion chromatography using NAP disposable columns. MALDI-MS: *m/z* = 11189 [M+Na]^+^ (MW = 11 166 calcd. For [C_385_H_563_N_151_O_187_P_28_]). ^1^H NMR (D_2_O with TSP (0.01 mM), 400 MHz): δ 0.79–0.86 (m, 48H, Leu- H^δ^), 1.13–2.38 (m, 147H, 28× H2’ and 28× H2’’ sugar ring protons, 5× CH_3_ of 5× dT, 16× Arg-H^β^, 16× Arg-H^γ^, 16× Leu-H^β^, 8× Leu-H^γ^, 12× CH_2_ (two aminohexyl linker), 2× acetyl-CH_3_), 3.17 (m, 16H, 16 × Arg-H^δ^), 3.51–4.45 (m,108H, 84× H4’/H5’H5’ sugar ring protons, 4× Gly-CH_2_, 16× Leu/Arg-H^α^), 4.95–6.32 (m, 33H, 28× H1’ sugar ring protons, 5× H5 of dC), 7.25–8.50 (m, 34H, 34× Ar-H from dG(H8 × 8), dA(H8 × 8), dA(H2 × 8), dC(H6 × 5) and dT(H6 × 5)). H3’ sugar ring protons (3.6–4.4 ppm) were not analysed due to the suppression of residual water signal at 4.10 ppm.

#### BC5-L-βα

RP-HPLC fraction at around 28 min was collected and lyophilized to give a white powder (19 nmol, 38%) (Supplementary Figure S6). Excess salt was removed by size-exclusion chromatography using NAP disposable columns. MALDI-MS: *m/z* = 11189 [M+Na]^+^ (MW = 11166 calcd. For [C_385_H_563_N_151_O_187_P_28_]) (Supplementary Figure S7). ^1^H NMR (Supplementary Figure S8) (D_2_O with TSP (0.01 mM), 400 MHz): δ 0.79–0.86 (m, 48H, Leu-H^δ^), 1.13–2.38 (m, 147H, 28 × H2’ and 28 × H2’’ sugar ring protons, 5× CH_3_ of 5× dT, 16× Arg-H^β^, 16× Arg-H^γ^, 16× Leu-H^β^, 8× Leu-H^γ^, 12× CH_2_ (two aminohexyl linker), 2× acetyl-CH_3_), 3.17 (m, 16H, 16 × Arg-H^δ^), 3.51–4.45 (m,108H, 84 × H4’/H5’H5’ sugar ring protons, 4 × Gly-CH_2_, 16 × Leu/Arg-H^α^), 4.95–6.32 (m, 33H, 28 × H1’ sugar ring protons, 5 × H5 of dC), 7.25–8.50 (m, 34H, 34 × Ar-H from dG(H8 × 8), dA(H8 × 8), dA(H2 × 8), dC(H6 × 5) and dT(H6 × 5)). H3’ sugar ring protons (3.6–4.4 ppm) were not analysed due to suppression of the residual water signal at 4.10 ppm.

#### BC5-L-βββ

RP-HPLC fraction at around 30 min was collected and lyophilized to give a white powder (16 nmol, 32%) (Supplementary Figure S6). Excess salt was removed by size-exclusion chromatography using NAP disposable columns. MALDI-MS: *m/z* = 11736 [M+Na]^+^ (MW = 11713 calcd. For [C_409_H_609_N_156_O_194_P_29_]) (Supplementary Figure S7).^1^H NMR (Supplementary Figures S8 and S9) (D_2_O with TSP (0.01 mM), 400 MHz): δ 0.78–0.88 (m, 54H, Leu-H^δ^), 1.91–2.39 (m, 181H, 29 × H2’ and 29 × H2’’ sugar ring protons, 5 × CH_3_ of 5 × dT, 18 × Arg-H^β^, 18 × Arg-H^γ^, 18 × Leu-H^β^, 9 × Leu-H^γ^, 18 × CH_2_ (three aminohexyl linker), 3 × acetyl-CH_3_), 3.17 (m, 18H, 18 × Arg-H^δ^), 3.62–4.33 (m, 111H, 87 × H4’/H5’H5’ sugar ring protons, 3 × Gly- CH_2_, 18 × Leu/Arg-H^α^), 5.03–6.35 (m, 34H, 29 × H1’ sugar ring protons, 5 × H5 of dC), 7.25–8.47 (m, 34H, 34 × Ar-H from dG(H8 × 8), dA(H8 × 8), dA(H2 × 8), dC(H6 × 5) and dT(H6 × 5)). H3’ sugar ring protons (3.6–4.4 ppm) were not analysed due to the suppression of residual water signal at 4.27 ppm.

Comparison of ^1^H NMR spectra of the ‘*single*’, ‘*bis*’ and ‘*triple*’ conjugates is given in Supplementary Figure S9 to demonstrate the expected stoichiometric ratios of the attached peptide moieties to the oligonucleotide component.

### Preparation of linearized plasmids and *in vitro* RNA transcripts

Linearized plasmid p67YF0 and *in vitro* transcript of yeast tRNA^Phe^ preparations were reported in detail earlier (61). Fluorescent labelling of RNA transcript with fluorescein isothiocyanate (FITC) at 3’-terminus followed our previously described protocol (61).

### Hybridization and cleavage of 3’-FITC tRNA^Phe^ with ‘*bis*’ and ‘*triple*’ conjugates

Hybridization assays and data analysis were carried out as previously reported (61), here 3’-FITC-tRNA^Phe^ (1 μM) was incubated with one of the ‘*bis*’ or ‘*triple*’ conjugates in excess (up to 20 μM).

Cleavage studies were also carried out according to the previously reported protocols (61). The reaction mixture contained 3’-FITC labelled tRNA^Phe^ (1 μM) and one of the ‘*bis*’ or ‘*triple*’ conjugates in variable excesses (5, 10 or 20 μM) of single turnover conditions in Tris Buffer (50 mM Tris–HCl pH 7.0 supplemented with 0.2 M KCl and 1 mM EDTA).

### Fluorescent ribonuclease activity assay

The reaction mixture (100 μl) contained fluorescently-labelled linear target RNA **F**-**Q**-RNA (1 μM) with fluorescein (**F**) and dabcyl quencher (**Q**) covalently attached to the internal deoxythymidine residues and one of the peptidyl-oligonucleotide conjugates (20 μM). Aliquots (10 μl) were taken at regular intervals (0, 24, 48, 72 and 96 h) during incubation of the reaction mixture at 37°C in 50 mM Tris−HCl pH 7.0 supplemented with 0.2 mM KCl and 1 mM EDTA. The combined effect of dilution (10-fold) of the collected aliquots with water and elevated temperature (80°C, 3 min) achieved complete dissociation of the cleaved RNA fragments from the conjugate. The extent of cleavage was evaluated by measuring the increase in fluorescence at λ_em_ of 522 nm (following excitation at 496 nm) at 37°C in microwell plates using a Tecan Safire plate reader operated under Magellan Data Analysis Software (V.7). The fluorescence of untreated **F**-**Q**-RNA was subtracted at each time-point to eliminate the background signal and normalized here to the complete (100%) cleavage of the same **F**-**Q**-RNA substrate achieved by RNase A (20 nM) in parallel control incubates. As controls, target was incubated both in the presence and absence of unconjugated oligonucleotide, and parallel measurements were conducted to rule out spontaneous cleavage. In addition, the target was incubated with a non-complementary scrambled conjugate (5’-luc-h-9/14) (63) to assess any possibility of non-sequence specific cleavage.

Multiple catalytic turnover experiments were carried out for selected ‘*single*’ conjugates (BC5-α or BC5-L-β) and one of the most active ‘*bis*’ conjugates (BC5-L-ββ) against linear fluorescently-labelled **F**-**Q**-RNA target present at 2-fold, 5-fold, 10-fold, 20-fold or 30-fold molar excess over conjugate. These reaction mixtures (100 μl) containing one of the 5-nt bulge–loop inducing conjugates (5 μM) and the corresponding molar excess of **F**-**Q**-RNA, which was present at 10 μM, 25 μM, 50 μM, 100 μM or 150 μM concentration, were incubated at 37°C over 96 h in 50 mM Tris buffer pH 7.0 supplemented with 0.2 mM KCl and 1 mM EDTA. Aliquots (10 μl) of each reaction mixture were taken after the same time intervals (0, 24, 48, 72 and 96 h) and similarly subjected to dilution (10-fold) in water and high temperature (80°C for 3 min) to dissociate reaction complexes. The extent of **F**-**Q**-RNA target cleavage by individual conjugates (BC5-α, BC5-L-β or BC5-L-ββ) was evaluated by measuring the increase in fluorescence intensity at each time point and normalizing it to the fluorescence of the fully-cleaved (100%) **F**-**Q**-RNA substrate by RNase A, as described in the previous section. These data were then used to estimate product concentration (or amount) to quantify the accumulation of the **F**-**Q**-RNA cleavage products. Kinetic parameters (*K*_m_, *V*_max_ and *K*_cat_) were estimated by analysis with a non-linear model to simulate change in initial reaction velocity (*V*_0_) with available initial substrate [S_0_] allowing for the formation of unreactive substrate and catalytic complexes (see Supplementary Information Section 21 for model details).

## RESULTS AND DISCUSSION

### Conjugate design and synthesis

**Figure 2.**
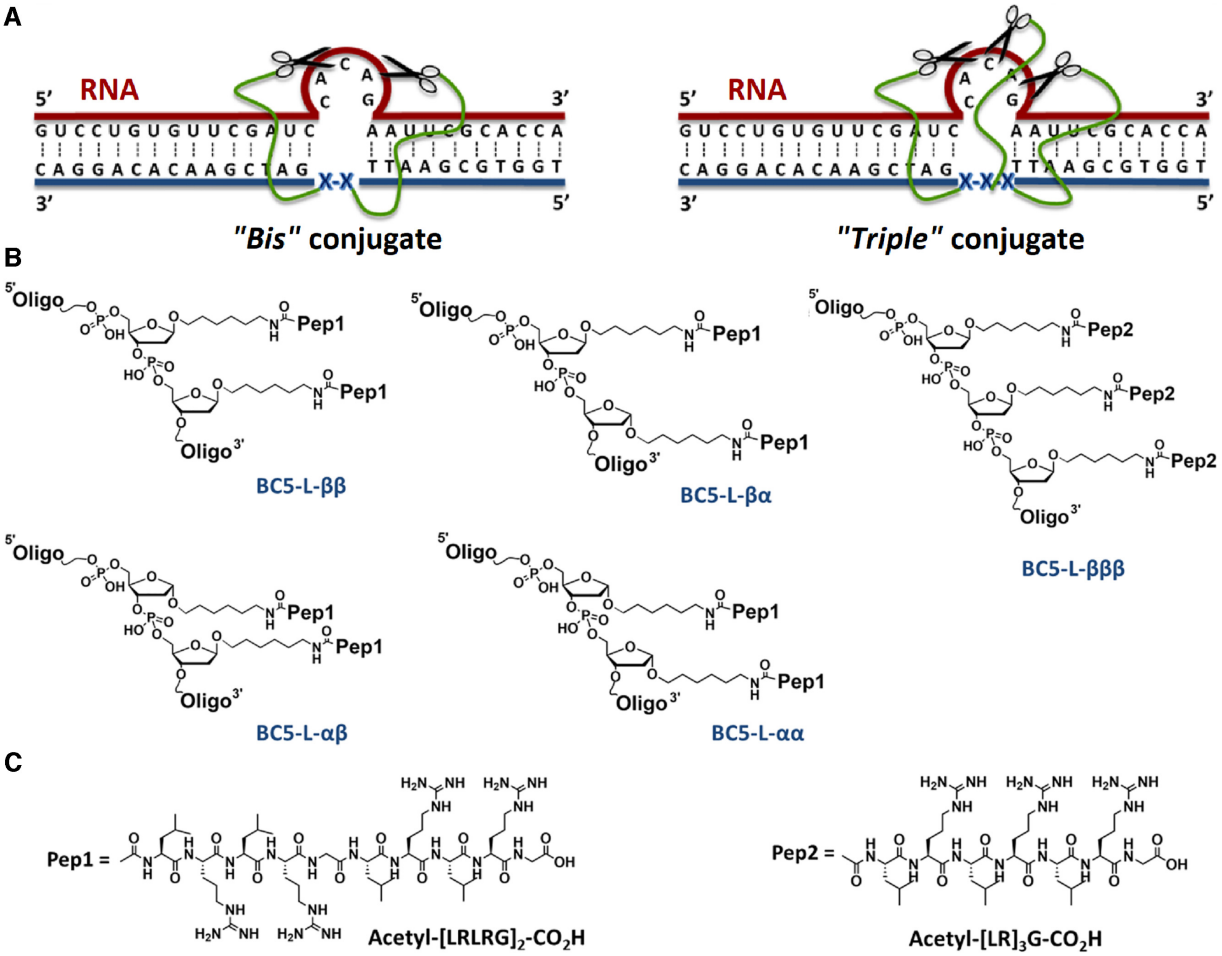
Design concept for the construction of ‘*bis*’ and ‘*triple*’ conjugates. (**A**) The bulge–loop inducing ‘*bis*’ (left) and ‘*triple*’ (right) conjugates (blue) bind to the target RNA region (red), corresponding to the 3’-acceptor stem, TΨC-arm and variable loop of tRNA^Phe^, given here as a primary structure. The anomeric nucleotides representing the point of the peptide attachment are shown here as **X**. The catalytic peptides are shown as scissors, and the aminohexyl linker between the peptide and oligonucleotide recognition motif is indicated as a flexible line in green. (**B**) Structural features of the peptide conjugation points for ‘*bis*’ (left and middle) and ‘*triple*’ (right) conjugates, where the catalytic peptide acetyl-[LRLRG]_2_-CO_2_H (Pep1) or Acetyl-[LR]_3_G-CO_2_H (Pep2) is conjugated to two or three internal abasic sugar residues *via* anomeric C1’-carbon through an aminohexyl linker attached in ββ, βα, αβ, αα or βββ-configuration. (**C**) Chemical structures of the catalytic peptides Pep1 and Pep2 used for ‘*bis*’ and ‘*triple*’ conjugation, respectively.

Multiple catalytic turnover in RNA cleavage requires each catalytic molecule (i.e. peptidyl-oligonucleotide conjugate) to cleave many copies of RNA substrate. This can only be achieved through rapid release of the cleaved RNA fragments after each cleavage event, to liberate the catalytic conjugate from the hybridized complex, to allow a new attack on the next RNA molecule (Figure 1) and achieve truly-catalytic destruction of multiple copies of specific RNA sequences. However, the high stability of *DNA–RNA* hybrids with long recognition motifs, essential to ensure absolute sequence-specificity, reduces the off-rate constant to non-catalytic levels, even after RNA cleavage. Herein, we address this challenge by reducing the binding affinity of the RNA cleavage fragments as compared to that of the intact, full-length RNA sequence. The peptidyl-oligonucleotide conjugates designed here induce a relatively large (5-nt) bulge–loop in the RNA upon *conjugate-RNA* hybridization, in order to provide several possible cleavage points in the exposed single-stranded RNA region. The opportunity to cut RNA at many positions is re-enforced further by incorporating several catalytic peptides into the same conjugate molecule to allow multiple attacks on the induced bulge–loop region (see Figure 2 for design). This design allows the number of accessible cleavage sites in the RNA to increase and raises the probability of forming *guanidine-guanidinium* dyads in their vicinity to boost cleavage frequency. Moreover, the shorter products of RNA cleavage with decreased binding affinity are expected to be readily displaced from the catalytic conjugate by intact RNA substrates *via* strand displacement cascades (71) to enable reaction turnover and realize the truly-catalytic ‘*bind, cleave and leave*’ turnover of RNA cleavage.

We engineered here a series of the ‘*bis*’ and ‘*triple*’ bulge–loop inducing conjugates that bind tRNA^Phe^ in a sequence-specific manner and form a single-stranded 5-nt bulge–loop ^61^C-^62^A-^63^C-^64^A-G^65^ in the TΨC-stem region, while maintaining Watson–Crick base pairing with the 3’-acceptor stem, TΨC-arm and variable loop (Supplementary Figure S10), as previously described for the analogous ‘*single*’ conjugates BC5-L-α and BC5-L-β (69). As a part of the design process, we screened the oligonucleotide recognition sequence of the conjugates against every possible 26-nt section within the 5’-part of the tRNA^Phe^ by ‘sliding’ it along this sequence with 1-nt incremental step to fully cover the sequence between 5’-terminal ^1^G residue and ^50^C nucleotide located in the beginning of the TΨC-arm (see Supplementary Figure S10). We calculated, using the DINAMelt Server (www.unafold.org), the thermodynamic parameters (Δ*G*, Δ*H* and Δ*S*) and *T*_m_ values for each potential complex between the recognition oligonucleotide and every 26-mer segment from the 5’-part of tRNA^Phe^ sequence. Some representative structures from this screening, along with the corresponding *T*_m_, Δ*G*, Δ*H* and Δ*S* values are shown in Supplementary Figure S10C–G. Not a single stable complex was discovered from this screening, which allowed us to eliminate the possibility of conjugate binding outside the target region. Either two or three peptide moieties were coupled to the oligonucleotide recognition motifs *via* aminohexyl linker attached to the anomeric C1’ carbon of the internal abasic sugar residues (**X**) to generate ‘*bis*’ or ‘*triple*’ conjugates, respectively, as illustrated in Figure 2.

Both the α- and β-configuration of peptide attachment was used in various combinations to produce a variety of stereospecific ‘*bis*’ conjugates (BC5-L-αα, BC5-L-ββ, BC5-L-αβ and BC5-L-βα) in αα, ββ, αβ or βα-configuration. This provided us with the opportunity to explore several possible conformational arrangements to enhance the probability of identifying orientations of the key players, vital for achieving potent RNA cleavage. A relatively long catalytic peptide Acetyl-[LRLRG]_2_-CO_2_H (Pep1) was chosen for ‘*bis*’ conjugation. This contained 10 amino acids, including four catalytically-important arginine residues scattered along the peptide chain (Figure 2C). The presence of the glycine residue in the middle of this peptide enhances the conformational flexibility of the RNA cleaving moiety, which, together with the extended peptide length, allows exploration of more energetically-favourable configurations for cleavage (72–75).

The ‘*triple*’ conjugate was designed to probe whether a more dense and regular arrangement of the catalytic arginine residues within the peptide scaffold offers any potential benefits for RNA cleavage. Accordingly, a shorter and less flexible heptapeptide Acetyl-[LR]_3_G-CO_2_H (Pep2) was chosen to generate a ‘*triple*’ conjugate *via* attachment to the three abasic sugar moieties located in the middle of the antisense recognition motif (see Figure 2). The overall number of catalytically-active arginine residues within this ‘*triple*’ conjugate was only marginally higher than those of the ‘*bis*’ conjugates (cf. 9 versus 8 arginines, respectively) to allow fair comparison of their catalytic performance against RNA. Given the slight superiority of the β-configuration in terms of RNA cleavage within the bulge–loops, previously observed for the ‘*single*’ conjugates (69), this configuration in peptide attachment was chosen for all three abasic sugar moieties, also to maintain the regular arrangement of the catalytic arginines within the ‘*triple*’ conjugate.

A shift in HPLC retention time from 17.5 min (average for starting oligonucleotides) to 28.5 min (average for ‘*bis*’ conjugates) and 30.3 min (‘*triple*’ conjugate) was observed, which was reproducible in all conjugation reactions (Supplementary Figure S6). The identities and purities of all ‘*bis*’ and ‘*triple*’ conjugates were confirmed by ^1^H NMR spectroscopy and MALDI-ToF/ToF spectrometry (see Supplementary Figures S7, S8 and S9, as well as Materials and Methods section for full characterization). The complete assignment of individual ^1^H NMR signals to the specific peptide and oligonucleotide protons is difficult due to the large number of overlapping peaks, especially in the peptide and oligonucleotide sugar ring region (0.5–4.7 ppm). However, careful signal integration, along with comparison between the NMR spectra of starting oligonucleotide and conjugates, allowed us to verify successful conjugation. For example, characteristic peptide signals, generated by Leu-H^δ^ and Arg-H^δ^ in resonance areas of 0.8–1.2 ppm and 3.1–3.4 ppm, respectively, are visible in the ^1^H NMR spectra of conjugates (see Supplementary Figure S8). Comparison of ^1^H NMR spectra of the ‘*single*’, ‘*bis*’ and ‘*triple*’ conjugates (see Supplementary Figure S9) further confirmed the expected stoichiometric ratios of the attached peptide moieties to the oligonucleotide component. For example, the presence of a distinguishable peak from Arg-H^δ^ protons at ∼3.2 ppm in all three spectra signifies the presence of the peptide component. Careful integration of this resonance signal against the oligonucleotide aromatic region (7.2–8.4 ppm), as well as H1’ sugar ring protons (5.4–6.4 ppm), confirmed 2:1 and 3:1 stoichiometric ratios of the peptide content to the oligonucleotide component in ‘*bis*’ and ‘*triple*’ conjugates, respectively.

### Hybridization and cleavage against tRNA^Phe^

The catalytic performance of ‘*bis*’ and ‘*triple*’ conjugates was assessed against fluorescently-labelled tRNA^Phe^ and compared with that of the analogous ‘*single*’ conjugates previously studied, against the same RNA target under identical experimental conditions (69). First, the ability of ‘*bis*’ and ‘*triple*’ conjugates to hybridize to tRNA^Phe^ was tested using the electrophoretic mobility shift assay (see Supplementary Figure S11), as the *RNA:conjugate* heteroduplex had a reduced electrophoretic mobility compared to unbound 3’-FITC-tRNA^Phe^. Association constants (*K*_a_) were estimated from the curve of the relative proportion of tRNA^Phe^ bound to conjugate as a function of conjugate concentration, and are given in Table 1, together with details of ‘*bis*’ and ‘*triple*’ conjugate nomenclature and composition.

**Table 1.** ‘*Bis*’ and ‘*triple*’ conjugate composition, nomenclature, and association constants (*K*_a_). *K*_a_ values were estimated using electrophoretic mobility shift assays carried out at 37°C

Conjugate	Sequence (5’→3’)	Peptide	*K*_a_ × 10^6^ M^–1^
**BC5-L-ββ**	TGGTGCGAATT-dR^β^-dR^β^-GATCGAACACAGGAC	Acetyl-[LRLRG]_2_	1.1 ± 0.9
**BC5-L-αα**	TGGTGCGAATT-dR^α^-dR^α^-GATCGAACACAGGAC	Acetyl-[LRLRG]_2_	3.5 ± 0.8
**BC5-L-αβ**	TGGTGCGAATT-dR^α^-dR^β^-GATCGAACACAGGAC	Acetyl-[LRLRG]_2_	4.3 ± 2.2
**BC5-L-βα**	TGGTGCGAATT-dR^β^-dR^α^-GATCGAACACAGGAC	Acetyl-[LRLRG]_2_	6.2 ± 2.3
**BC5-L-βββ**	TGGTGCGAATT-dR^β^-dR^β^-dR^β^-GATCGAACACAGGAC	Acetyl-[LR]_3_G	0.5 ± 0.1

Where ${K_a} = \frac{\alpha }{{{{[ {{\rm BC}} ]}_0}( {1 - \alpha } )\,( {1 - \alpha ( {\frac{{{{[{\rm{tRNA]}}}_0}}}{{{{[ {{\rm BC}} ]}_0}}}} )} )}}$, where α = fraction bound, [tRNA]_0_= total tRNA^Phe^ concentration and [BC]_0_= total conjugate concentration.

All conjugates were capable of binding to tRNA^Phe^ in a concentration-dependent manner (see Supplementary Figure S11). The use of relatively long recognition motifs (overall, 26 nt) compensated for the potential destabilization effects, which arise from the large RNA loop (5 nt) induced upon hybridization, and from the presence of two or even three abasic nucleotide residues in the middle of the oligonucleotide chain. Conjugates demonstrated saturation binding to tRNA^Phe^, where the plateau (with 100% binding) was reached at a conjugate concentration around or slightly above 5 μM for the ‘*bis*’ conjugates and above 10 μM for the ‘*triple*’ conjugate (Supplementary Figure S11). Comparisons of ${K_a}$ values (see Table 1) indicated that incorporation of the additional peptide in α-configuration favoured the binding affinity of ‘*bis*’ conjugates towards the RNA target. Indeed, the presence of at least one peptide in the α-configuration (i.e. BC5-L-αα, BC5-L-αβ and BC5-L-βα) enhanced their association constants in comparison to those of their ‘*single*’ counterparts (cf. BC5-L-α, *K*_a_ = 0.9 ± 0.6 × 10^6^ M^–1^ and BC5-L-β, *K*_a_ = 0.5 ± 0.2 × 10^6^ M^–1^) (69). The extra peptide chain seemed to stabilize the hybridized complex, presumably through additional electrostatic interactions between the negatively-charged RNA chain and the positively-charged peptide. Such a stabilizing effect was also observed for the previously-reported linear conjugates with the 5’-terminal attachment of the amphiphilic peptides (76). The difference in the association constants between BC5-L-αα, BC5-L-αβ and BC5-L-βα did not exceed statistical errors (Table 1). In contrast, incorporation of the additional peptide(s) in β-configuration to form BC5-L-ββ and BC5-L-βββ did not improve their binding affinities (within statistical errors) as compared to that of their ‘*single*’ counterpart BC5-L-β (*K*_a_ = 0.5 ± 0.2 × 10^6^ M^–1^) (69), but instead led to a statistically-significant reduction of their association constants, as compared to those seen for the other conjugates with multiple peptide attachment. Indeed, BC5-L-ββ and BC5-L-βββ conjugates showed the lowest binding constants (*K*_a_ = 1.1 ± 0.9 × 10^6^ M^–1^ and *K*_a_ = 0.5 ± 0.1 × 10^6^ M^–1^, respectively), thus suggesting that the attachment of the peptide(s) in the β-configuration may lead to less favourable binding conformations, which seem to annul the stabilizing effect arising from any additional interactions.

**Figure 3.**
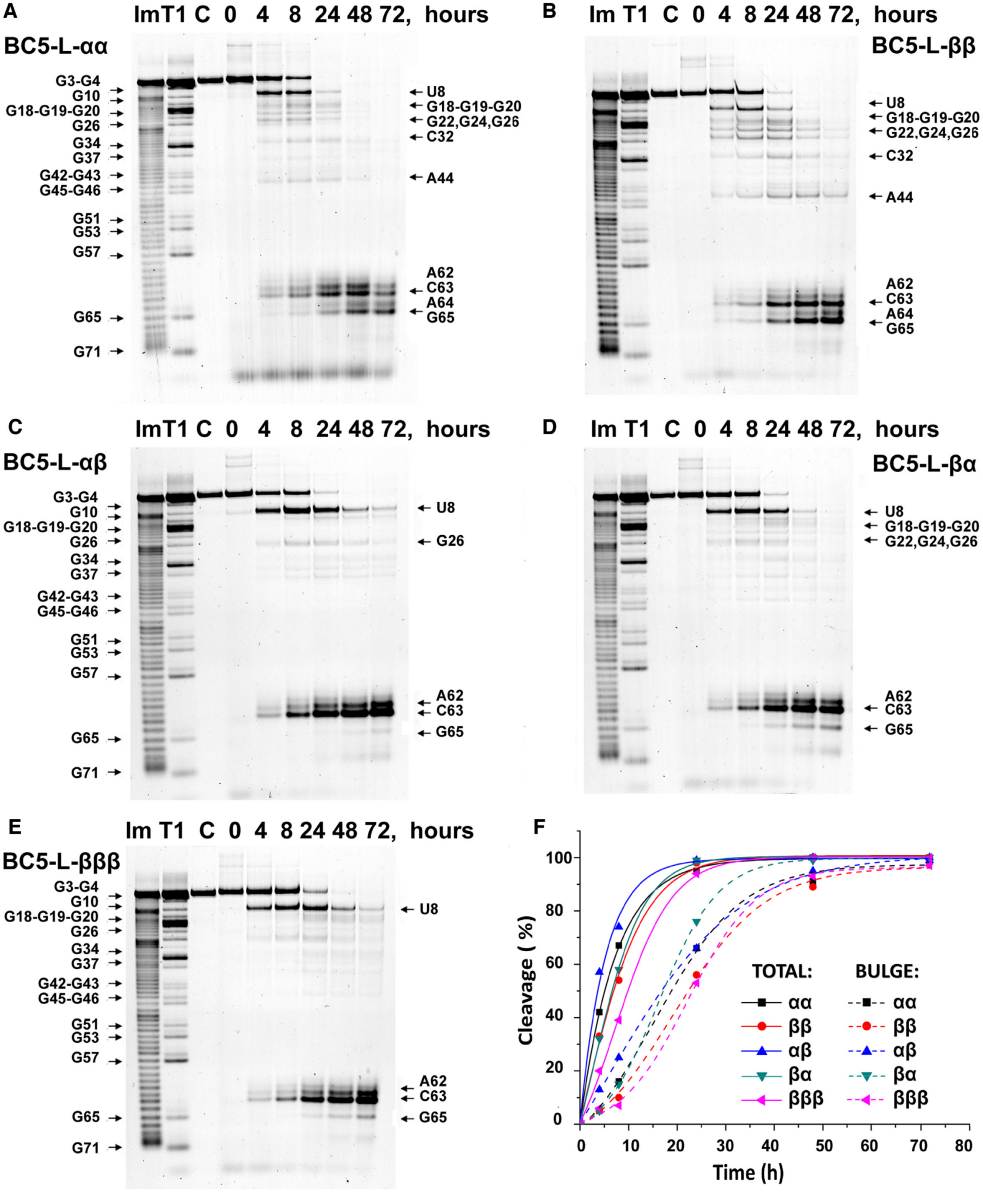
Cleavage of 3’-FITC-tRNA^Phe^ with ‘*bis*’ and ‘*triple*’ conjugates. (A–E) Representative images of 12% PAAM/8 M urea gel electrophoresis of cleavage products of 3’-FITC-tRNA^Phe^ with BC5-L-αα (**A**), BC5-L-ββ (**B**), BC5-L-αβ (**C**), BC5-L-βα (**D**) and BC5-L-βββ (**E**) with incubation time. Digestion ladders of 2 M imidazole (lane Im) and RNase T1 (lane T1) were used for identification of cleaved fragments. Lane C was 3’- FITC-tRNA^Phe^ incubated in the absence of conjugate for 72 h. 3’-FITC-tRNA^Phe^ (1 μM) was incubated with different ‘*bis*’ and ‘*triple*’ conjugates in excess (each 20 μM) at 37°C. (**F**) Total extent of cleavage (%, solid lines) and extent of cleavage in bulge–loops (%, dashed lines) of ‘*bis*’ and ‘*triple*’ conjugates (computed from density scans of gels A–E).

Cleavage activities of ‘*bis*’ and ‘*triple*’ conjugates were first assessed against 3’-FITC-tRNA^Phe^ under physiological conditions (pH 7.4, 37°C) over 4, 8, 24, 48 and 72 h and analysed by 12% PAGE electrophoresis under denaturing conditions. A large 20-fold excess of conjugates (20 μM) over tRNA^Phe^ (1 μM) ensured that even the conjugates with the lowest binding affinity (e.g. BC5-L-ββ and BC5-L-βββ) reached full hybridization. The cleavage sites were identified through comparison with RNase T1 and 2 M imidazole tRNA^Phe^ hydrolysis ladders (see Figure 3A–E). Extent of RNA cleavage over time (Figure 3F) was analysed both in terms of ‘*total*’ degradation of tRNA^Phe^ at all detected cleavage sites (plotted against time as solid lines; ‘TOTAL’), and in terms of cleavage measured exclusively at bulge–loop regions (plotted as dashed lines; ‘BULGE’). No spontaneous cleavage was seen in the absence of conjugate (lane C in Figure 3A–E). Attachment of multiple catalytic peptides into the structure of ‘*bis*’ and ‘*triple*’ conjugates led to a significant enhancement in their ribonuclease activity against tRNA^Phe^, with almost quantitative (93–100%) degradation of the target RNA in 24 h (see Figure 3F, solid lines), whereas their ‘*single*’ counterparts BC5-L-α and BC5-L-β studied previously (69) were able to reach, in total, only 73% and 83% RNA cleavage after much longer incubation period (72 h), under exactly the same conditions. The ‘*triple*’ conjugate exhibited slightly slower cleavage kinetics as compared to ‘*bis*’ conjugates, informing us that more dense, rigid, and regular arrangement of the catalytic residues offered no advantage for cleavage, even with an extra guanidinium group present in the structure of BC5-L-βββ. The lesser cleavage activity of the ‘*triple*’ conjugate can be attributed to shortening the peptide length and removal of the central glycine residue, which reduced the structural flexibility that facilitates cleavage (62,69).

Most importantly, ‘*bis*’ and ‘*triple*’ conjugates showed considerably increased targeted cleavage of tRNA^Phe^ at the induced bulge–loop region ^61^C-^62^A-^63^C-^64^A-^65^G (Figure 3F, dashed lines), compared to their analogous ‘*single*’ conjugates (69). All conjugates with multiple catalytic peptides achieved a complete (100%) destruction of the induced bulge–loops within 72 h of incubation, whereas their ‘*single*’ counterparts BC5-L-α and BC5-L-β were able to reach only 30% and 54% of cleavage at the bulge–loop region under identical conditions (69). ‘*Bis*’ conjugate BC5-L-βα took only 48 h to achieve 100% cleavage at the bulge–loops, followed closely by BC5-L-αβ and BC5-L-αα.

**Figure 4.**
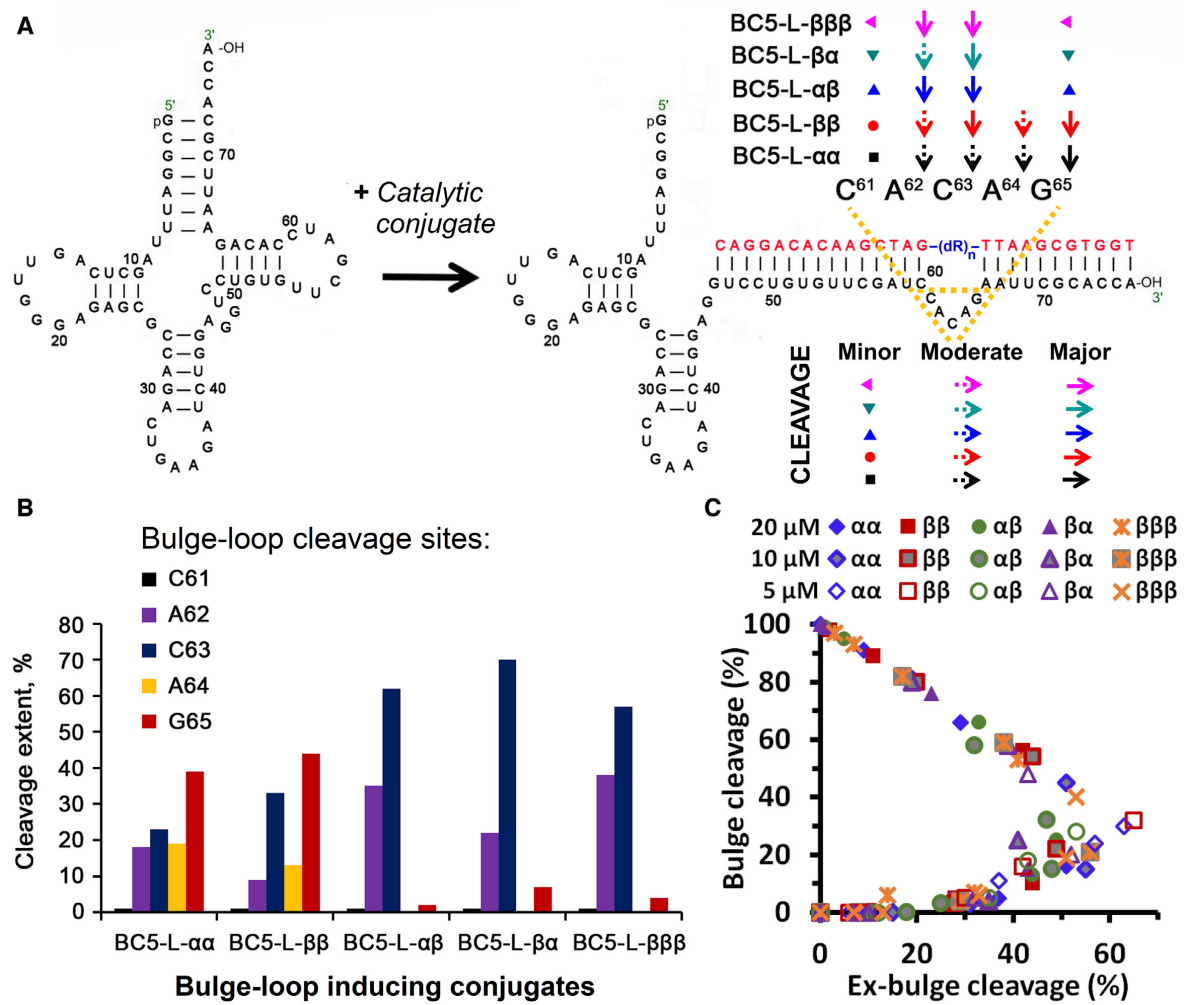
(**A**) Hybridization of ‘*bis*’ and ‘*triple*’ conjugates (red) induces a 5-nt long bulge–loop around the ^61^C-A-C-A-G^65^ region of the tRNA^Phe^ (black), thus increasing its susceptibility to cleavage. Major and minor cleavage sites within this region are colour coded for each individual conjugate and indicated by arrows and dots. (**B**) Extent of cleavage (%) at individual positions of the induced bulge–loop (^61^C-A-C-A-G^65^) after 72 h exposure of the target tRNA^Phe^ to the catalytic ‘*bis*’ or ‘*triple*’ conjugates. (**C**) Common pathway of tRNA^Phe^ cleavage by all conjugates at different concentrations (5, 10 and 20 μM), with a threshold of 30–40% cleavage in nearby folds before bulge–loop cleavage took over (data taken from Figure 3F and Supplementary Figures S12 and S13).

Two distinctive cleavage patterns were observed amongst ‘*bis*’ and ‘*triple*’ conjugates in the bulge–loop region (Figure 4). BC5-L-αα and BC5-L-ββ ‘*bis*’ conjugates with two catalytic peptides in the same configuration (either αα or ββ) were able to attack the entire loop and cleave it at five different positions, with a predominance of the ^65^G-^66^A site, followed by the ^63^C-^64^A > ^64^A-^65^G > ^62^A-^63^C >> ^61^C-^62^A positions. Overall, BC5-L-αα and BC5-L-ββ conjugates seemed to be more versatile, both in terms of their ability to produce cuts at multiple sites and to catalyse cleavage of a variety of phosphodiester linkages, including G-X, C-A, A-C and even A–G. In contrast, the attachment of two catalytic peptides in opposite configurations (either αβ or βα) in BC5-L-αβ and BC5-L-βα ‘*bis*’ conjugates led to a relatively narrow, but sharp cleavage with only two major cuts in the target region ^61^C-^62^A-^63^C-^64^A-^65^G. It is evident from Figures 3 and 4 that the ^63^C-^64^A position became the most vulnerable when tRNA^Phe^ was attacked by either the BC5-L-αβ or BC5-L-βα conjugate, whereas the ^62^A-^63^C site was less favourable. The other two sites (^65^G-^66^A and especially ^61^C-^62^A) showed only minor cleavage contributions, not exceeding 8% even when taken together. Cleavage at A–C and especially at C-A phosphodiester bonds showed strong predominance over that at G-X linkages.

Such peculiar difference in cleavage profiles between these two categories of ‘*bis*’ conjugates suggested that the spatial orientation of the two catalytic peptides against each other seems to control whether they act independently or jointly in the catalysis. RNA cleavage requires the coordinated attack of two guanidinium groups from the opposite sides of the RNA backbone (see Figure 1B and Figure 8 in (69)), one acting as a general base to deprotonate the 2’-OH group of RNA ribose and triggering the formation of a pentaoxyphosphorane intermediate; while the other (protonated guanidinium) coordinates the negatively-charged phosphate group, and acts as an electrophilic activator (70). In the case of ‘*bis*’ and ‘*triple*’ conjugates, such guanidinium groups can be supplied either by the same peptide chain to allow two catalytic peptides to act independently from each other, or by two different peptide moieties, which requires them to approach the RNA backbone from opposite sides in a highly-synchronized manner. The first mode of cleavage is less conformationally demanding in terms of mutual orientations of the peptide moieties and is expected to produce more cuts at multiple locations, which was observed here for the BC5-L-αα and BC5-L-ββ conjugates (Figures 3 and 4). Presumably, the identical configuration in the attachment of the peptides restricted their ability to approach the RNA backbone from opposite sides, necessary for the synchronized catalytic action, and forced them to act mainly independently. Indeed, the analogous ‘*single*’ conjugates BC5-L-α and BC5-L-β (69) showed similar ability to cleave the whole bulge–loop region at several positions. Given that such ‘*single*’ conjugates provide guanidinium groups from the same peptide and therefore can only realize the first catalytic mode, their similarity in cleavage patterns with those of BC5-L-αα and BC5-L-ββ supports this hypothesis. In contrast, the synchronized engagement of two separate peptides in the catalysis requires a high level of precision, both in their action and in spatial orientation, and therefore is expected to be more selective in terms of cleavage sites and base specificity. In the case of BC5-L-αβ or BC5-L-βα conjugates, the opposite configuration in attachment of the peptide seems to allow them to approach the RNA backbone from opposite directions, and engage in a coordinated action to produce fewer, but more intense cuts, without compromising overall cleavage efficiency.

Interestingly, the ‘*triple*’ conjugate BC5-L-βββ, with all three peptide moieties attached in the same configuration showed similar cleavage patterns to those seen for BC5-L-αβ and BC5-L-βα ‘*bis*’ conjugates, thus suggesting possible joint action of the separate peptide moieties in the catalysis. Presumably, the shorter and less flexible Acetyl-(LR)_3_G-CO_2_H peptide restricts the possibility for each chain to form a *guanidine-guanidinium* dyad for independent catalytic action. The attachment of the peptide moieties at the three different abasic nucleotides seems to separate them sufficiently in space, which is further reinforced by a helical turn of the *RNA-DNA* hybrid duplex. This seems to allow these short peptides to approach the RNA backbone from opposite directions and supply arginine guanidinium groups for the synchronized catalytic action.

The greater potency of ‘*bis*’ and ‘*triple*’ conjugates was also reflected by full cleavage of 3’-FITC-tRNA^Phe^ (up to 100%) at lower conjugate concentrations (10 μM and 5 μM) with lower molar excess of the conjugate over the target (with *conjugate:RNA* molar ratio of 10:1 and 5:1; Supplementary Figures S12 and S13), albeit lower conjugate concentrations showed correspondingly slower cleavage kinetics.

Due to the flexible attachment and overall length of the catalytic constructs, the ‘*bis*’ and ‘*triple*’ conjugates were also able to reach and attack other structural elements of tRNA^Phe^, folded nearby the site of their sequence-specific hybridization (Figures 3A–E), where their overall behavior remained similar to that seen towards the hybridized target region ^61^C-^62^A-^63^C-^64^A-^65^G. Indeed, BC5-L-αβ, BC5-L-βα and BC5-L-βββ produced fewer cuts in nearby folding, mainly limited to the ^8^U-^9^A position, with the minor cleavage contributions from two G-rich regions of the D-arm (^18^G-^20^G and ^22^G-^26^G). As expected, BC5-L-αα and BC5-L-ββ were able to produce more cuts at nearby folded locations in addition to those seen for the BC5-L-αβ, BC5-L-βα and BC5-L-βββ conjugates, which included two extra cuts in the anticodon and variable loops (^32^C-^33^U and ^44^A-^45^G, respectively). In agreement with the available X-ray structure of yeast tRNA^Phe^ (77), all identified cleavage sites were within reach (≤45 Å) of the longer acetyl-(LRLRG)_2_-CO_2_H peptide in its unfolded conformation (45.1 Å) or became conformationally accessible to the catalytic guanidinium groups (61,62,69). The shorter acetyl-(LR)_3_G-CO_2_H peptide (34.2 Å) incorporated into the BC5-L-βββ structure was mainly able to reach the D-arm upon hybridization with tRNA^Phe^, but not the less proximal regions of the anticodon loop. Remarkably, these minor products of the nearby cleavage were accumulated during the first few hours of the cleavage reaction, and started to diminish rapidly after 8 h, followed by their complete disappearance at around 48 h (for BC5-L-βα) or 72 h (for BC5-L-αβ, BC5-L-αα, BC5-L-ββ and BC5-L-βββ). This was accompanied by faster accumulation of the cleavage products at the induced bulge–loop ^61^C-^62^A-^63^C-^64^A-^65^G (Figure 3A–E) in a co-operative manner (reflected by the sigmoidal-like dotted curves on Figure 3F), ultimately reaching 97–100% cleavage. These results suggest that the ^8^U-^9^A junction and G-rich regions located in the D-arm were more vulnerable for initial cleavage, or more favourable for formation of the catalytically-competent *guanidine-guanidinium* dyad for the initial cuts, which can trigger conformational rearrangements of the RNA tertiary structure thus making it more relaxed and flexible for attack in the induced bulge–loop ^61^C-^62^A-^63^C-^64^A-^65^G. Initial cleavage products remaining bound to the conjugates may be attacked again at their induced bulge–loops to generate much shorter cleavage products. Some representative examples of 3’-FITC-tRNA^Phe^ cleavage products upon treatment with ‘*bis*’ or ‘*triple*’ conjugates, detected by electrophoretic analysis (Figure 3), are shown in Supplementary Figure S14. To summarize, the ‘*bis*’ and ‘*triple*’ bulge–loop inducing conjugates engineered here produced diverse and complex RNA cleavage profiles due to a higher degree of conformational freedom of the peptides flexibly anchored to the abasic sugar within the oligonucleotide recognition motif. Such artificial ribonucleases may show considerable cleaving activity outside the RNA region directly engaged in hybridization with the conjugate recognition motif, which might be advantageous in achieving effective catalytic destruction of a long RNA sequence with complex secondary and tertiary structural elements (*e.g*. long non-coding RNA or viral genomic RNA).

The sigmoidal character of the cleavage in bulge–loops (see Figure 3F and Supplementary Figures S12B and S13B, dotted curves) suggests that cleavage at this region starts slowly within the intact folded tRNA^Phe^ structure, but rapidly accelerates as the structure becomes more relaxed and flexible. As clearly illustrated by Figure 4C, when the extent of bulge–loop cleavage was plotted against nearby cleavage elsewhere (‘*ex-bulge*’ cleavage), the cleavage behavior of tRNA^Phe^ by all conjugates at different concentrations (5 μM, 10 μM and 20 μM) followed the same distinctive path. Remarkably, up to 30–40% of nearby cleavage outside the bulge appeared necessary, in all cases, before reaching the point where bulge cleavage emerged and then progressively took over. The same cleavage path was followed at all concentrations (see Supplementary Figure S15), and only the kinetics (velocity and cleavage extent) changed with different concentrations of conjugates. This emphasizes the dominant role of the folded structure of tRNA^Phe^ in the cleavage pathway. At 20-fold molar excess of conjugates, the initial velocities of total cleavage approached or exceeded 100 nM/h, but declined steeply as the target tRNA^Phe^ was rapidly consumed, which remained the dominant feature of the kinetics at 10:1 molar excess with initial velocities approaching 50 nM/h (Supplementary Figure S16). However, at 5:1 molar excess, differences in the velocity of bulge–loop cleavage could be clearly seen for individual conjugates. More specifically, the initial velocities of ‘*bis*’ conjugates with identical peptide configurations (BC5-L-αα and BC5-L-ββ) were retained, whereas more ‘conformationally demanding’ conjugates (BC5-L-αβ, BC5-L-βα and BC5-L-βββ) had low initial velocities but, with few cuts outside the bulge–loop, appeared to show an increase in velocity over time, despite the decline in the concentration of tRNA^Phe^ substrate (Supplementary Figure S16D). The above data suggest that long RNA fragments derived from initial cleavage of nearby folds remain hybridized with the conjugates, until they diminish in size by subsequent cleavage at bulge–loops (shown by Supplementary Figure S14).

As evident from Figures 3F and Supplementary Figures S12B and S13B, the kinetics of RNA cleavage is strongly affected by conjugate concentrations. For example, after 24 h of treatment of tRNA^Phe^ (1 μM) with BC5-L-βα, the overall cleavage extent reached 100%, 66% or 37% when the conjugate concentration was 20, 10 or 5 μM, respectively, under otherwise identical experimental conditions. A similar trend was seen for the other four bulge–loop inducing conjugates BC5-L-αα, BC5-L-ββ, BC5-L-αβ and BC5-L-βββ, as well as for the previously studied ‘*linear*’ (61) and ‘*dual*’ (62) peptidyl-oligonucleotide conjugates. Such conjugate concentration-dependence of catalytic efficiency of RNA cleavage cannot be fully explained by a greater proportion of hybridized complex. As seen from the gel-shift analysis of hybridization between ‘*bis*’ and ‘*triple*’ conjugates and 3’-FITC-tRNA^Phe^ (Supplementary Figure S11), at 5 μM conjugate concentrations all ‘*bis*’ conjugates were fully bound (93–100%) to the RNA target, although the ‘*triple*’ conjugate achieved this level of binding at slightly higher (10 μM) concentration. One possible explanation of this phenomenon is that in aqueous solutions such supramolecular conjugates co-exist in multiple conformational forms, with only some being ‘catalytically active’ against RNA (62). A higher conjugate concentration is therefore needed to increase the number of conjugate molecules existing in their ‘catalytically-active’ forms, capable of RNA cleavage at the detectable level. Another hypothesis might be self-assembly of several conjugate molecules into ‘productive’ multiplexes with greater cleavage capability (62,63,66,78). Certainly, the empirical observation here was that the excess conjugate concentrations increased the cleavage kinetics in nearby folds allowing greater cleavage in the induced bulge–loops.

The incorporation of multiple arginine-rich amphiphilic peptide residues into the structure of ‘*bis*’ and ‘*triple*’ conjugates led to a greater level of RNA demolition, especially at the induced bulge–loop regions, when quantitative (100%) cleavage was achieved. An enhanced probability to form the catalytic *guanidine-guanidinium* dyads, essential for catalysis, combined with the high degree of conformational freedom of the cleaving constructs flexibly linked to the RNA-recognizing motifs, seem to be crucial for efficient destruction of long RNA sequences with complex secondary and tertiary structural elements. However, the complexity of biological RNA substrates makes it difficult to assess the possibility of the catalytic turnover reaction, especially when they form supramolecular complexes with the peptidyl-oligonucleotide conjugates. Indeed, multiple-turnover catalysis usually requires a significant excess of RNA substrates (up to 30-fold) over the catalytic molecules, thus resulting in a high concentration of RNA sequences in solution. This may trigger some level of substrate aggregation, which can be further facilitated by formation of multiple peptide bridges between different *RNA-conjugate* hybrids, stabilized by electrostatic interactions between the positively-charged peptides and the negatively-charged sugar-phosphate backbones of nucleic acids (76). Consequently, in order to study reaction kinetics in a comparable manner to enzymes, we devised a simple, linear fluorescently-labelled RNA lacking such secondary and tertiary structure, so that supramolecular RNA structure did not dominate the kinetics.

### Assessment of ribonuclease activity by fluorescent assay

**Figure 5.**
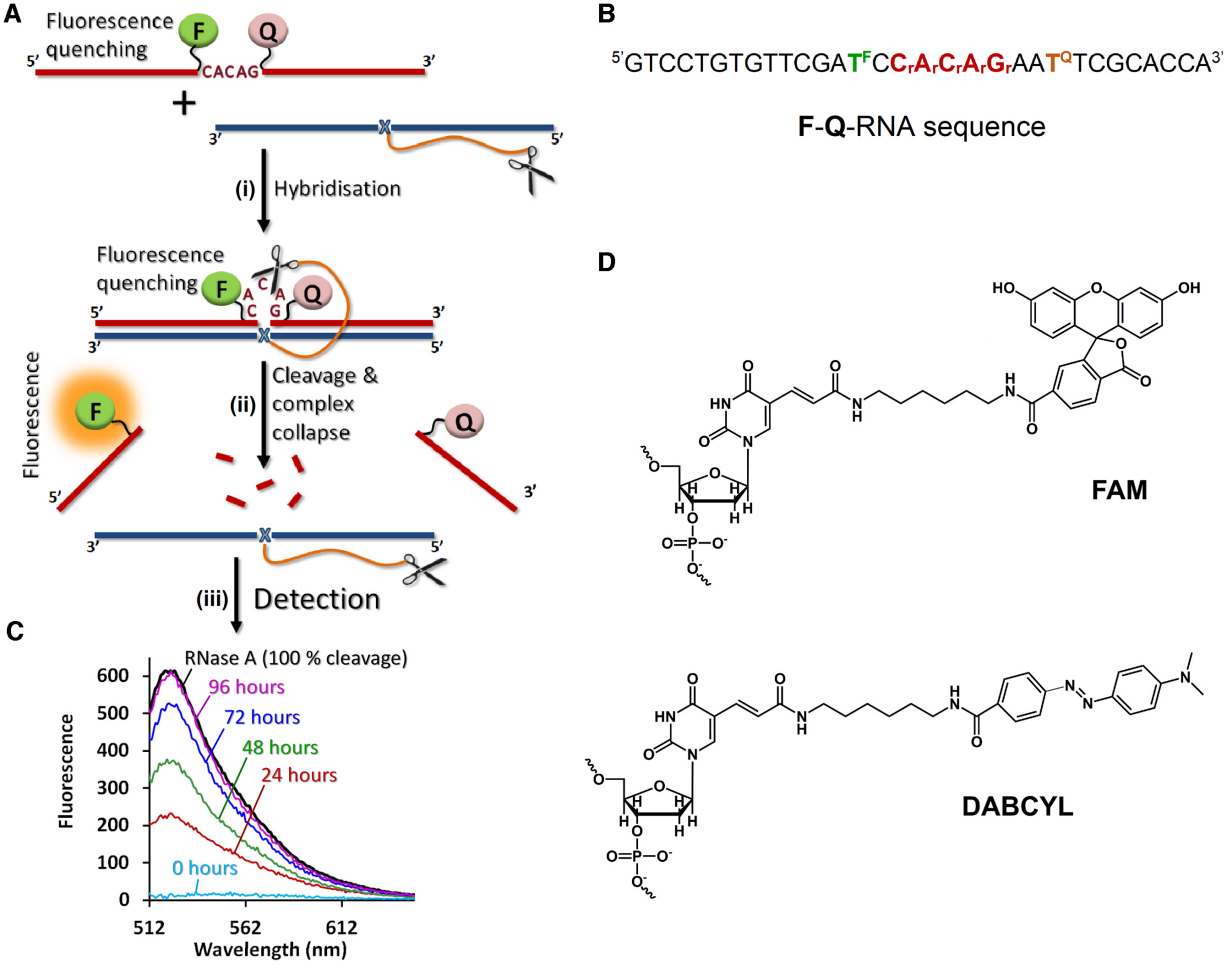
Fluorescent assay of RNA bulge–loop cleavage and dissociation. (**A**) Schematic illustration of (i) binding of conjugate (blue) to the labelled **F**-**Q**-RNA target (red), leading to (ii) formation of the bulge–loop (**-C_r_A_r_C_r_A_r_G_r_-**), which brings the two reporter groups (**F** and **Q**) into close proximity, quenching fluorescence. Cleavage of the target at the bulge–loop regions (iii) allows the cleaved target to dissociate, when fluorescence increases as fluorophore and quencher separate. (**B**) Linear model target **F**-**Q**-RNA was constructed here as a *RNA-DNA* hybrid sequence, incorporating an internal fluorescein (**F**, in green) and dabcyl quencher (**Q**, in dusky pink) covalently attached to the deoxythymidine residues, which flanked the bulge–loop region and were positioned 8-nt apart from one another. The sequence corresponding to the bulge–loop region (shown in red) is made of ribonucleotides. (**C**) Progressive increase in fluorescence over time is quantified against maximum fluorescence of the fully cleaved (100%) **F**-**Q**-RNA target by RNase A (black curve). (**D**) Chemical structures of incorporated deoxythymidine residues with covalently-attached fluorescein (**F**) and dabcyl (**Q**).

We developed a fluorescent assay to study the kinetics of cleavage in a linear RNA substrate, lacking the supramolecular complexities of the tRNA^Phe^ substrate, which otherwise obfuscated the observation of differences between the kinetic behaviour of individual conjugates. The linear model RNA substrate also allowed us to benefit from the advantages of fluorescence resonance energy transfer (FRET, see Figure 5). The model target (**F**-**Q**-RNA) had fluorophore (**F**) and quencher (**Q**) at the positions flanking the bulge–loop region (**-C_r_A_r_C_r_A_r_G_r_-**) composed of the ribonucleotide residues, while the rest of the **F**-**Q**-RNA sequence comprised deoxyribonucleotide residues, which are resistant to cleavage by ribonucleases, including the chemical (arginine-mediated) catalysts studied here. This design allowed us to monitor cleavage exclusively in bulge–loops by eliminating cuts outside this region. The nucleotide sequence of this 31-mer model target matched precisely the region corresponding to the 3’-acceptor stem, TΨC-arm and variable loop of tRNA^Phe^, in order to allow comparisons with our tRNA^Phe^ studies. The bulge–loop inducing conjugate (Figure 5A, in blue) hybridized with the **F**-**Q**-RNA target (in red) in a sequence-specific manner, thus forcing formation of the bulge–loop region in the middle of the sequence. The size of the induced loop could be varied by the length of the non-complementary sequence in the oligonucleotide recognition motifs, so that either the entire RNA region (**C_r_A_r_C_r_A_r_G_r_**) was vulnerable for cleavage or lesser parts (<5-nt) could be exposed to catalytic attack (Figure 5B). The intact RNA was fully quenched and fluorescently silenced (Figure 5C, cyan curve) by the proximity of **F** and **Q** (Figure 5A, steps (i) and (ii)). Cleavage of **F**-**Q**-RNA at the exposed bulge–loop region triggered dissociation and separation of **F** and **Q** (Figure 5A, step (iii)), thus leading to de-quenching and increased fluorescence (Figure 5C, red, green, blue and purple curves) to allow quantitative monitoring of cleavage over lengthy periods.

Selected ‘*bis*’ (BC5-L-αα and BC5-L-ββ) and ‘*triple*’ (BC5-L-βββ) conjugates were compared with previously studied (69) ‘*single*’ conjugates with the catalytic peptide attached in α or β anomeric configuration, capable of inducing bulge–loops of varying sizes of 2-nt (BC2-α and BC2-β), 3-nt (BC3-α and BC3-β), 4-nt (BC4-α and BC4-β) or 5-nt (BC5-α, BC5-β and BC5-L-β). The number in the nomenclature of ‘*single*’ conjugates indicated the size of the induced bulge. Since ‘*single*’ conjugates were also designed to target the 3’-acceptor stem, TΨC-arm and/ variable loop of tRNA^Phe^ (69), the complete sequence similarity between the target regions of the linear **F**-**Q**-RNA and tRNA^Phe^ enabled these ‘*single*’ conjugates to form bulge–loops around the **C_r_A_r_C_r_A_r_G_r_** sequence upon hybridization.

Hybridization of conjugates to the fluorescently-labelled **F**-**Q**-RNA target (1 μM) was confirmed by a sharp decline in fluorescence, which reached a plateau of full binding at around 10 μM conjugate concentration and had association kinetics similar to that of *unconjugated* oligonucleotide (see Supplementary Figure S17). Formation of a bulge–loop structure in the target sequence upon hybridization seemed to position the fluorophore and quencher even closer to each other. The closely-located aromatic bases of the hybridized conjugate, as well as the covalently-attached peptide moieties may also contribute to the observed quenching, which was slightly smaller when the related *unconjugated* oligonucleotides were bound to the **F**-**Q**-RNA target.

**Figure 6.**
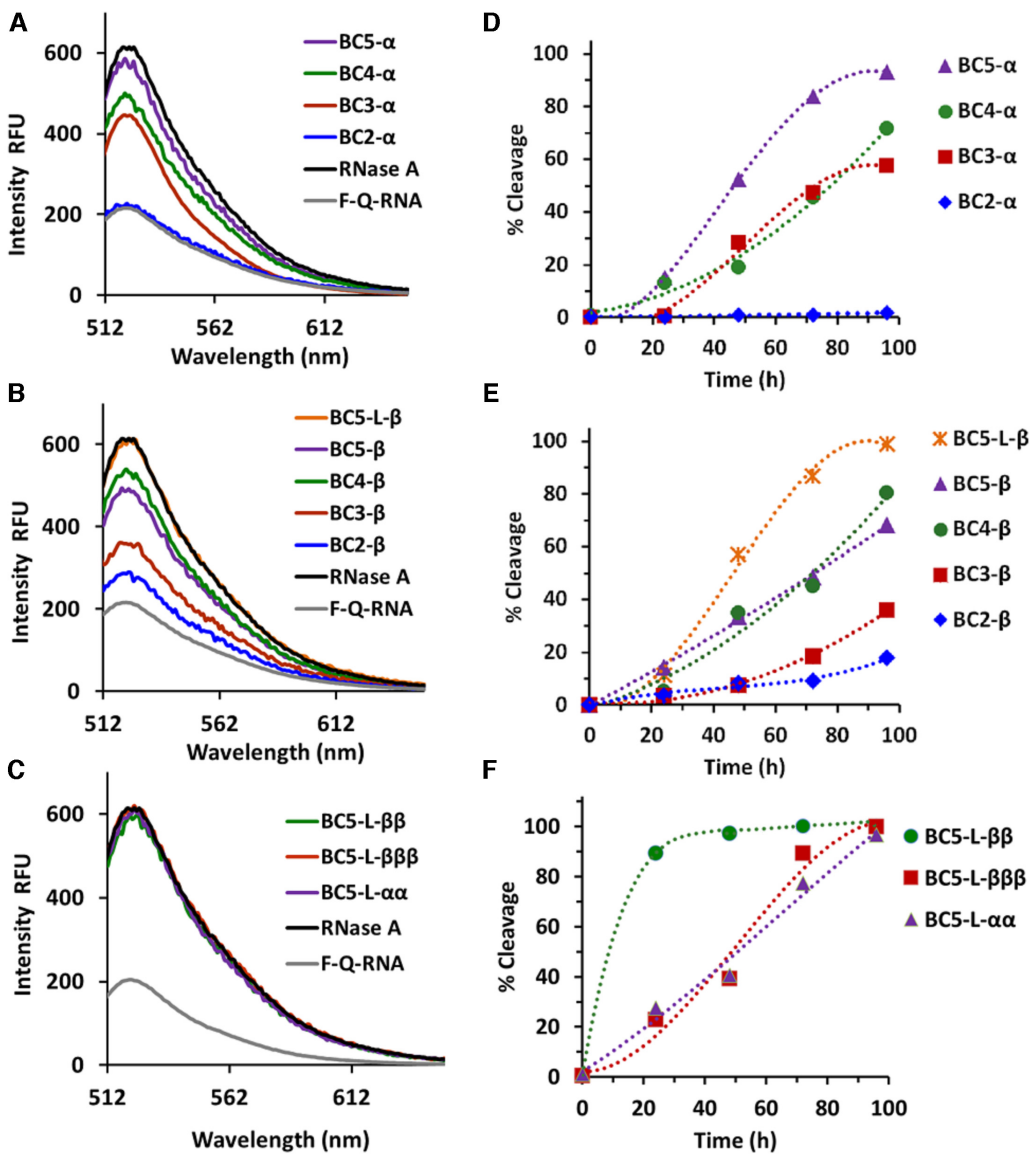
Left column: End-point fluorescence detection of **F**-**Q**-RNA cleavage by various ‘*single*’ α-conjugates (**A**) and β-conjugates (**B**), as well as by ‘*bis*’ (BC5-L-αα and BC5-L-ββ) and ‘*triple*’ (BC5-L-βββ) conjugates (**C**). The target **F**-**Q**-RNA (1 μM) was treated with one of the conjugates (20 μM) over 96 h, and fluorescence was measured following excitation at 496 nm and compared with that of the fully cleaved **F**-**Q**-RNA by RNase A (20 nM; black curve). The background fluorescence of untreated **F**-**Q**-RNA target is also shown (grey curve). Right column: Kinetics of **F**-**Q**-RNA cleavage by ‘*single*’ α-conjugates (**D**) and β-conjugates (**E**), and by ‘*bis*’ and ‘*triple*’ conjugates (**F**). Relative cleavage (%) at each time point (0–96 h) was calculated by measuring fluorescence emission at 522 nm upon 496 nm excitation, followed by subtracting background fluorescence of untreated **F**-**Q**-RNA and scaling to 100% cleavage produced by RNase A of the same **F**-**Q**-RNA substrate (see **‘**Materials and Methods**’** for further details).

Except for BC2-α showing negligible cleavage of the smallest (2 nt) bulge–loop, all ‘*single*’ conjugates catalysed cleavage within the bulge–loop of **F**-**Q**-RNA, and their cleavage efficiency increased with the size of the bulge–loop induced (Figure 6D and 6E). For example, after 72 h of incubation, the extent of the F-Q-RNA cleavage was increased from the negligible level (1.0%) seen for BC2-α in the smallest bulge–loop (2 nt), through the medium level seen for BC3-α and BC4-α (47.3% and 45.6%, respectively), to much higher level (83.9%) in the largest loop (5-nt) induced by BC5-α (see Figure 6D). A similar trend was seen for the β-category of the ‘*single*’ conjugates, with the extent of cleavage gradually increasing from 9.1% for BC2-β to 18.4% for BC3-β, 45.1% for BC4-β, 48.4% for BC5-β and 86.7% for BC5-L-β (see Figure 6E). Similar increase in cleavage with the size of the induced bulge–loop was also seen for the tRNA^Phe^ target (69), where the expansion of the bulge region led to an increase in the number of cuts within the analogous target region ^61^C-^62^A-^63^C-^64^A-^65^G. However, the extent of cleavage seen here for the linear **F**-**Q**-RNA target was roughly doubled for each ‘*single*’ conjugate as compared to that observed for tRNA^Phe^ earlier (69). This indicates the desired greater accessibility of the bulge–loop region in the simpler linear **F**-**Q**-RNA target compared to the folded tRNA^Phe^ target. The 5-nt bulge–loop inducing conjugates exhibited the highest overall cleavage (93.2% for BC5-α and 99.0% for BC5-L-β after 96 h, Figure 6). From the *back-to-back* comparisons of the related BCn-α and BCn-β conjugates inducing bulge–loops of the same size, the catalytic performance of α-series ‘*single*’ conjugates seemed slightly superior to those from the β-series, except for BC2-α, which was inactive. No spontaneous cleavage was seen when non-complementary (scrambled, 5’-luc-h-9/14) conjugate was used against **F**-**Q**-RNA, nor from unconjugated complementary oligonucleotide, verifying the absence of non-specific cleavage (Supplementary Figure S18).

Incorporation of multiple catalytic groups into the conjugate structure had greatest impact, particularly attachment of two catalytic peptides in β-configuration, which led to considerable enhancement (see BC5-L-ββ in Figure 6F), rapidly reaching 100% cleavage. Although this conjugate was also very active against tRNA^Phe^ (Figure 3B and F), the cleavage kinetics observed for BC5-L-ββ against the short linear **F**-**Q**-RNA target were much quicker than those seen for the complex biological tRNA^Phe^. Indeed, nearly 90% of **F**-**Q**-RNA was degraded within the first 24 h, with more than 97% of cleavage achieved in 48 h. Surprisingly, **F**-**Q**-RNA cleavage by BC5-L-αα and BC5-L-βββ were similar to their ‘*single*’ counterparts (cf. 96.7% for BC5-L-αα versus 93.2% for BC5-α, 100% for BC5-L-βββ versus 99% for BC5-L-β, Figure 6). However, they showed much higher ribonuclease activity towards the biological tRNA^Phe^ target (see Figure 3), when the cleavage within the bulge–loops roughly doubled after attachment of the additional two or three peptide chains (cf. with 30% for BC5-L-α, and with 54% for BC5-L-β (69)), thus re-enforcing the overall advantage of multiple catalytic groups for catalysis.

### Multiple turnover catalysis of RNA cleavage

**Figure 7.**
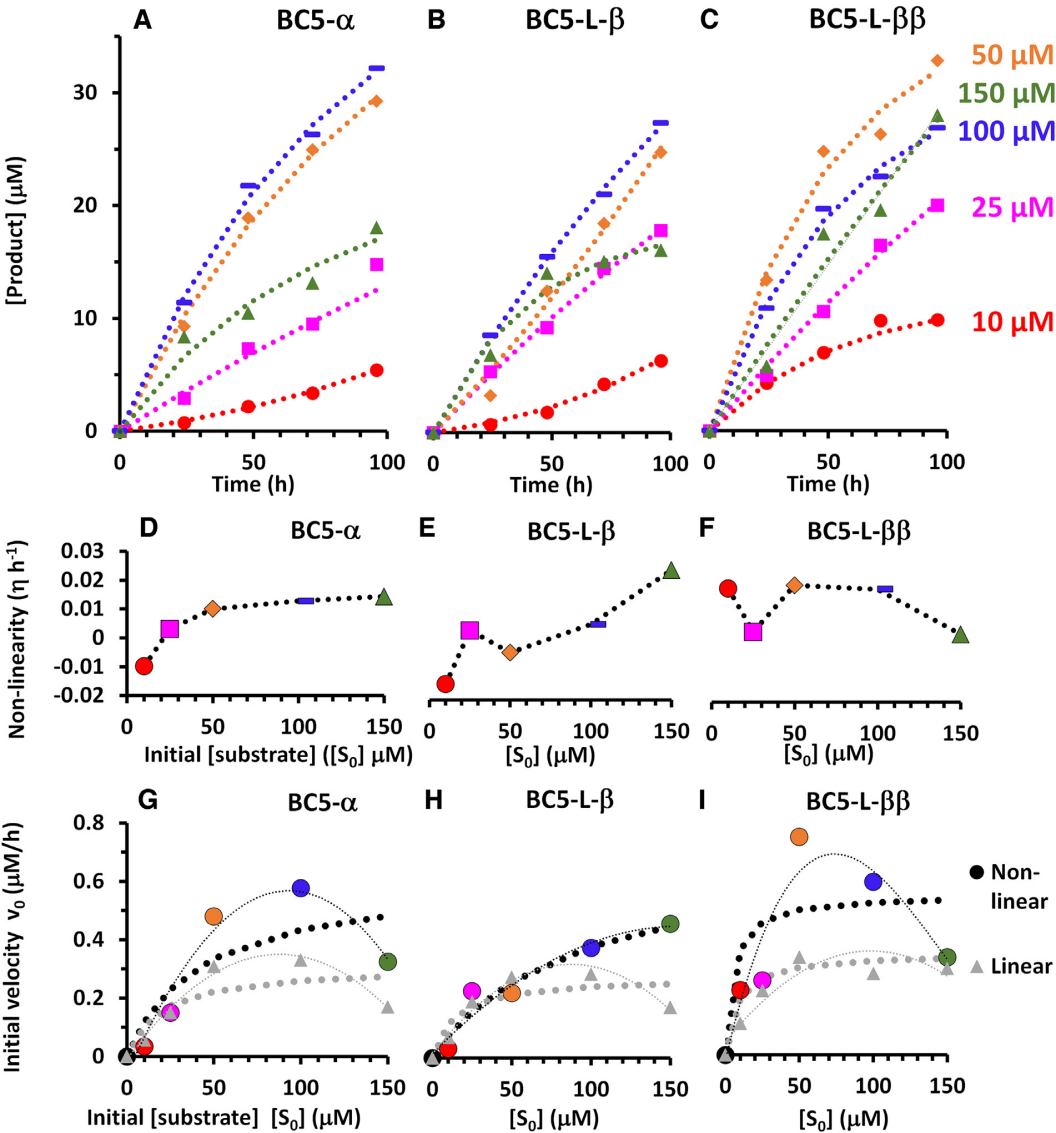
Atypical kinetics of RNA cleavage by catalytic conjugates under multiple turnover conditions. Each conjugate (5 μM) was incubated with up to 30-fold excess of the **F**-**Q**-RNA substrate present at 10 μM (red), 25 μM (pink), 50 μM (orange), 100 μM (purple) or 150 μM (green) concentration. Top row (**A–C**): Progress curves of cleavage product concentration [P], measured from increase in fluorescence and normalized against full cleavage of each substrate concentration ${[ S ]_0}$ by RNase A. Middle row (**D–F**): Non-linearity factor ($\eta$) of cleavage kinetics as a function of substrate concentration. Bottom row (**G–I**): Initial velocities$\;( {{v_0}} )\;$of **F**-**Q**-RNA cleavage plotted against initial substrate concentrations. Non-linear curve fitting of $[ P ] = \frac{{{v_0}}}{\eta }\;( {1 - {e^{ - \eta t}}} )$ (59,60) estimated non-linearities $\eta$ and initial velocities ${v_0}\;$and revealed atypical parabolic kinetics for both non-linear (corresponding colour-filled circles) and linear (grey-filled triangles) estimates of initial velocities ${v_0}$, rather than hyperbolic kinetics of Michaelis–Menten (dotted traces: black for non-linear and grey for linear estimation of initial velocities).

Multiple turnover kinetics of the most active ‘*bis*’ conjugate (BC5-L-ββ) was investigated using the same fluorescent assay, but with up to 30-fold excess of the linear target **F**-**Q**-RNA over the conjugate, and was compared with that of the most active representatives of ‘*single*’ conjugates with the shortest (BC5-α) and longest (BC5-L-β) antisense recognition motifs. Each conjugate (5 μM) was incubated with 2-, 5-, 10-, 20- or 30-fold excess of the **F**-**Q**-RNA substrate present at 10, 25, 50, 100 or 150 μM concentration, respectively, when cleavage kinetics was followed over 96 h (see Figure 7A–C), as described in **‘MATERIALS AND METHODS’**. Quantitative estimation of **F**-**Q-**RNA cleavage by ‘*single*’ and ‘*bis*’ conjugates under multiple-turnover conditions is given in Table 2. It is seen that each conjugate was able to cleave multiple RNA copies, with the highest number of cleaved **F**-**Q-**RNA molecules of 6.6, 6.4 and 5.6 copies per conjugate achieved by BC5-L-ββ, BC5-α and BC5-L-β, respectively. The increase in *RNA:conjugate* molar ratio (up to 10- and/or 20-fold) raised the number of cleaved **F**-**Q-**RNA molecules initially, which later declined with the further increase in substrate concentration. Surprisingly, the highest excess of the RNA substrate (20-fold and/or 30-fold) appeared to be detrimental for cleavage, showing lower than expected number of cleaved RNA substrates (Table 2). This correlated with the slower cleavage kinetics seen for all three conjugates at the highest **F**-**Q-**RNA concentrations (100 and/or 150 μM), when the molar excess of the RNA substrate exceeded 20- or 30-fold (see Figure 7).

**Table 2.** Quantification of **F**-**Q-**RNA cleavage by ‘*single*’ and ‘*bis*’ conjugates under multiple-turnover conditions with up to 30-fold excess of RNA target. Each conjugate (0.5 nmol) was incubated with 2-, 5-, 10-, 20- or 30-fold excess of the **F**-**Q**-RNA substrate in 100 μl Tris buffer at 37°C, over 96 h^a^

		BC5-L-ββ		BC5-L-β		BC5-α	
Initial F-Q- RNA substrate		Cleaved F-Q- RNA		Cleaved F-Q RNA		Cleaved F-Q RNA	
Concentration (μM)	Amount (nmol)	Amount (nmol)	Number of molecules	Amount (nmol)	Number of molecules	Amount (nmol)	Number of molecules
10	1.0	1.0	***2.0***	0.6	***1.2***	0.6	***1.2***
25	2.5	2.0	***4.0***	1.8	***3.6***	1.5	***3.0***
50	5.0	3.3	***6.6***	2.5	***5.0***	2.9	***5.8***
100	10.0	2.7	***5.4***	2.8	***5.6***	3.2	***6.4***
150	15.0	2.8	***5.6***	1.6	***3.2***	1.8	***3.6***

^a^Experimental details *are* given in **‘**Materials and Methods**’**.

The observed initial velocities of the conjugate-catalysed cleavage of excess substrate showed consistent behaviour that could not be explained by simple Michaelis Menten kinetics, in three main regions (see Figure 7G–I). (i) When substrate was in small excess (2- and 5-fold), initial velocities were lower than expected and then increased with substantial depletion of substrate into product. (ii) With substrate in larger excesses (10- and 20-fold), initial velocities were higher than could be contiguous with the above velocities, and then declined with substrate conversion to product. (iii) At higher substrate excesses (from 20- to 30-fold), initial velocities were much lower than could be contiguous with the above velocities and declined as substrate was converted to product. To understand better this atypical kinetics of RNA cleavage by catalytic conjugates under the multiple turnover conditions, we developed a simulation model of the complex dynamic nature of interactions between the catalytic molecules and RNA substrates (see also Supplementary Section 21).

Given the non-linear, often sigmoidal nature of reaction progress curves, non-linear estimation of initial reaction velocities (${v_0}$) was used throughout (79,80). Progress curves approached linearity only for the 5-fold molar excess of substrate (Figure 7A–C), where a consistently small non-linearity $\eta \sim 0$ held for all BC5 conjugates (Figure 7, pink-filled squares in D–F, respectively). However, at 2-fold molar excesses, small increases in velocity with negative non-linearity ($ - \eta$) were evident for the single peptide conjugates (Figure 7, red-filled circles in D–E), suggestive of activation during the reaction progress at low substrate concentration, not apparent for the ‘*bis*’ conjugate BC5-L-ββ. Otherwise, velocities decreased during reaction progress with positive non-linearity (+$\eta$), suggestive of the decline of the substrate and/or increase in inhibition by the product.

Strikingly, the most active conjugates (BC5-α and especially BC5-L-ββ) suffered from lower velocities at larger substrate excesses (20-fold and 30-fold), resulting in the atypical parabolic kinetic profiles (Figure 7G and I). However, the least-active elongated ‘*single*’ conjugate (BC5-L-β) approximated to a more hyperbolic profile of initial velocities reminiscent of Michaelis–Menten kinetics, but again with deviations at lower substrate excesses and the strongest decline in velocities with reaction progress at 30-fold substrate excess (Figure 7B, E and H).

**Figure 8.**
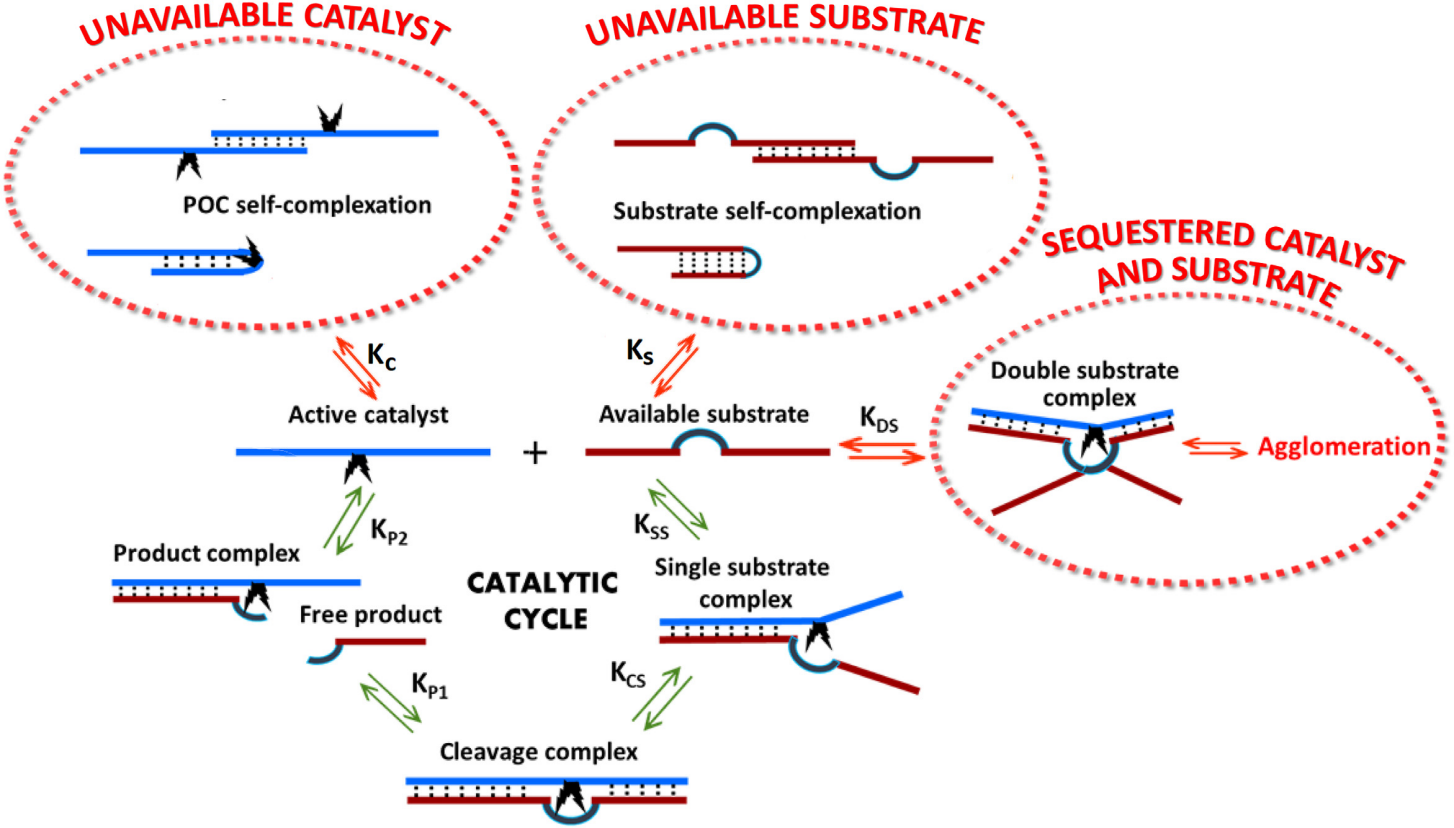
Model of possible dominant interactions of catalyst, its substrate and products. Additional to the catalytic cycle (green arrows), where active catalyst binds available substrate and forms cleavage products from a cleavable substrate complex, weaker self-associations are possible from sequence homologies within each component. Such side interactions sequester available substrate and inactivate the catalyst into complexes (red arrows), which do not then participate in the catalytic cycle. A dynamic numerical model of the equilibria, with equilibrium dissociation constants (*K*) characterized the interactions, under different conditions and during the catalytic reaction (Supplementary Section 21).

An explanation of this discrepancy was found in the likelihood of self-association of the target substrate and conjugates into hairpin and duplex structures. Hairpin structures involving intramolecular folding are particularly stable and can be formed rapidly (see Supplementary Figure S19). When dominant self-association is considered, both the un-complexed substrate available for reaction and its decline during reaction progress are much reduced, as complexed substrate can replace or buffer the available substrate consumed (see Supplementary section 21 for further details, and Supplementary Figures S20 and S21). Similar self-association affecting the availability of conjugate may also result in the observed activation and inactivation of catalytic conjugate. The developed numerical model (see Supplementary Section 21) combines the main such interactions and is schematically summarized in Figure 8, as a first possible explanation to simulate the atypical kinetic data observed.

The RNA substrate, which is expected to be complexed ${[ S ]_c}$, was calculated from its equilibrium dissociation ${K_s}{\rm{\;}}$ and the total substrate ${[ S ]_0}$, when the expected available substrate ${[ S ]_a}$ was obtained by difference. The complexed conjugate and its activation in competition with the initially-available substrate ${[ S ]_a}$ were calculated from its approximated dissociation ${K_c}{\rm{\;}}$ to provide the activated concentration of conjugate ${[ C ]_a}$ available dynamically during reaction progress. Initial approximated values were refined within the non-linear numerical fitting of the model to observed data (Supplementary Section 21).

The expected weaker association of cleaved product and similar partial binding of substrate to the catalytic conjugate, in competition with the full binding of substrate (Figure 8) were calculated from their equilibrium dissociation constants ${K_P},$${K_{SS}}$ and ${K_{CS}}$ (respectively). Intact substrate will dominate the occupancy of the conjugate initially but, as substrate is cleaved, product occupancy rose to <10% in this model. Products of cleaved substrate were assumed to have similar affinity to that of partial binding of substrate in this model, in order to approximate the occupancy of product (Supplementary Figure S22). At large excess of substrate, some of the conjugate fraction will be ‘doubly occupied’ by a pair of partially-bound substrate molecules (${K_{ds}}$) (Figure 8), which will decline in competition with cleaved products partially occupying one half or the other of the binding site. Given strongly-affected reaction velocities at higher substrate concentrations, the rate of decline in this ‘double-substrate’ occupancy of the conjugate will be greater at lower than at higher substrate concentrations, when ‘double occupancy’ would be retained for longer periods of reaction. It will also remain elevated at the higher substrate concentrations, where the conjugate was also prone to inactivation (Supplementary Figure S22). The two dangling ends of partially-bound substrate molecules and the two or more dangling positively-charged peptides are likely to promote agglomeration, which is expected to inactivate the catalytic conjugate. Inactivation will thereby increase with the greater extent and longer duration of ‘double-substrate’ occupancy at higher substrate concentrations (Figure 8). The lesser rate of decline of ‘double-substrate’ occupancy of the conjugate with increasing substrate concentrations (Supplementary Figure S22) was considered as the key determinant of inactivation in this first model, in addition to the other inactivation routes (*e.g*. inhibition of the conjugate by the generated product). The activation route of the self-associated (and thus *unavailable*) conjugate (Supplementary Figure S21) proceeds *via* binding with the available substrate, which can unfold the hairpin structures or disrupt imperfect *conjugate-conjugate* self-associations by forming much stronger *conjugate-substrate* complexes stabilized by a larger number of perfect-match Watson-Crick base-pairing.

**Figure 9.**
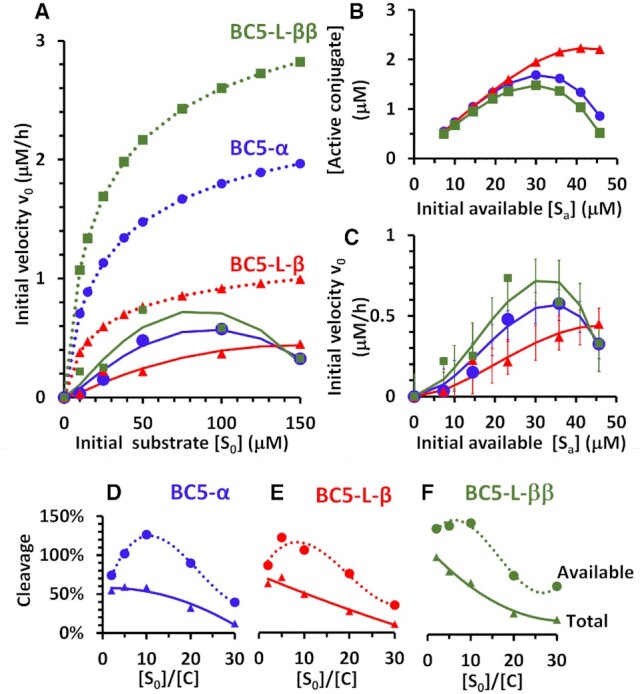
Active conjugate kinetics. (**A**) Michaelis–Menten parameters (${K_m},{k_{cat}}$) indicated kinetics expected for fully-activated conjugates (dotted traces) for the fit to observed velocities for active conjugate (continuous traces), estimated from (**B**) the numerical model of dynamic change in active conjugate for the available substrate. (**C**) Initial velocities expected from the numerical model for available substrate after non-linear fitting to observed velocities with error bars representing standard error at 95% confidence limits. (**D–F**) Extent of cleavage of initially-available substrate (dotted traces of circles) and total substrate (continuous traces of triangles) during multiple turnover kinetics for different molar ratios of total substrate to conjugate.

Michaelis–Menten kinetics for initial velocities (${v_0}$) and initial *available* substrate ${[ S ]_{a0}}$, including the active conjugate ${[ C ]_a}$ (in the ${V_{max}} = {[ C ]_a}{\rm{\;}} \times {k_{cat}}$ term) were used to estimate ${k_{cat}}$, ${K_m}$ and to refine approximated equilibrium dissociation characteristics (${K_c}$,${\rm{\;}}{K_s},{\rm{\;}}{K_{ss}}$). Of the total conjugate, only a minor fraction (∼10%) was initially available at small excesses of substrate. This model of activation by available substrate, and inactivation in larger excess of substrate, allowed Michaelis–Menten kinetics to approach the observed velocities (Figure 9 and Supplementary Figure S23).

The conjugate with two catalytic peptides (BC5-L-ββ, ${k_{{\rm cat}{\rm{\;}}}}0.8{\rm{\;}}{{\rm h}^{ - 1}}$) showed the greatest ${V_{max}}$ (Figure 9 (A) and Supplementary Table S2), but was approached by the conjugate with a single peptide from the α anomer (BC5-α, ${k_{{\rm cat}}}{\rm{\;}}0.6{\rm{\;}}{{\rm h}^{ - 1}})$, whereas attaching the single peptide from the β anomer with an elongated recognition motif cut activity by half (BC5-L-β,${\rm{\;}}{k_{{\rm cat}}}{\rm{\;}}0.29{\rm{\;}}{{\rm h}^{ - 1}}$). However, reflective of identical sequence complementarity, similar Michaelis–Menten constants ${K_m}{\rm{\;}}$were estimated in the range $20\hbox{--}24{\rm{\;}}\mu {\rm M}$. The dynamic changes in the activation of conjugates were similar, as was the profile of inactivation of ‘*single*’ α and ‘*bis*’ ββ conjugates, whereas the single elongated β conjugate was least inactivated at higher substrate excess (see Figure 9B and Figure S23 for details).

Importantly, in all three cases, the extent of cleavage approached 100% or greater of the *initially- available* substrate for total substrate excess up to 5 -10-fold. The conjugates with a single catalytic peptide showed similar extents of cleavage, whereas the conjugate with a pair of catalytic peptides had a greater extent of cleavage, presumably, through rapidly consuming much of the self-complexed substrate too (expressed as cleavage of initially-available substrate greater than 100%). However, beyond 10-fold substrate excess, the extent of cleavage declined, presumably from conjugate inactivation (Figure 9D–F), and because the proportion of the expected product-occupied conjugate increased towards 10% (Supplementary Figure S22). Product inhibition presented as uncompetitive with *K_i_* of $18\hbox{--}21{\rm{\;}}\mu {\rm M}{\rm{\;}}$(Supplementary Table S2). Velocities expected from Michaelis–Menten kinetics with uncompetitive product inhibition approached observed velocities for the upper substrate excesses, where available substrate remained buffered and steady during reaction progress (Supplementary Figure S24).

Full cleavage of target substrate at 10–20-fold molar excess offers considerable potency where a single catalyst molecule destroys 5–10 targets, even with a partially-active conjugate (<30% in Figure 9D). Simulations of activated conjugate revealed that under physiological charge screening and in the presence of emerging product fragments, leading to reduced self-association and therefore decreased conjugate inactivation, and at substrate excesses with little inactivation of conjugate (∼10 [S]/[C], Figure 9), each conjugate may be capable of destroying over 30 target molecules. Such catalytic potency could allow a few active molecules to knock down the typical range of copy numbers of pathogenic RNA in each cell and to continue to do so for many days.

Activation of catalysis can be enhanced even further through rigorous conjugate design, for example, by distancing the attachment points of the positively-charged catalytic peptides in the oligonucleotide chain. This may promote an *intra*molecular repulsion between 5’- and 3’-terminal segments of the conjugate and minimize its folding into stable hairpin structures, detrimental for catalysis. By manipulating the network of anticipated atomic interactions within the structural components, it is possible to control the secondary structures of such conjugates and avoid their inactivation. Recently developed toehold-mediated strand displacement and toehold exchange mechanisms (71,81) through either three-way branch migration (71,81–83) or hidden thermodynamic drive (71,84) can also be used, both to reduce self-associations of conjugates and RNA substrates into inactive complexes, and to accelerate rapid release of catalysts from hybridized complexes. This may allow them to operate continuously and increase substrate turnover even further.

Indeed, these relatively small artificial ribonucleases attacked and unfolded the higher order structure of tRNA, probably by sequence-specific displacement, initiated from the toehold provided by the 3’ single-stranded overhang, combined with the extended reach of peptides to cleave nearby folds. Generally however, accessibility remains a challenge shared with other RNA-silencing approaches, where RNA target sites can reside within complex secondary and tertiary structures that many biological RNA molecules adopt, which may lead to variable success biologically. Reliable identification of accessible regions within target RNA sequences is even more important when RNA silencing is reliant upon access by bulkier enzyme complexes. Computational approaches remain helpful for prediction of possible tertiary RNA structures (85–87), but actual accessibility can be probed by various chemical and enzymatic techniques, for example: hybridization of RNA with a random pool of complementary DNA fragments (88) or chemically-synthesized chimeric oligodeoxynucleotides (89), followed by digestion with RNase H and fragment identification by gel electrophoresis (88) or sequencing using primer extension analysis (89); self-quenching oligonucleotide probes with internal loop-stem structures (90); RNA target binding to combinatorial arrays of oligonucleotides (91,92); RNA backbone exposure mapping through hydroxyl radical cleavage (93), with RNA footprinting analysis (94,95) including massive parallel-sequencing as the readout for hydroxyl radical footprinting (96), and simultaneous analysis of multiple RNA sequences by combining random priming of reverse transcription with barcoding (97).

The field of RNA targeting by oligonucleotide-based agents is advancing rapidly. However, translational development of these catalytic conjugates and their future applications in cells, tissues and *in vivo* require them to demonstrate sufficient serum stability against intracellular and extracellular nucleases, which is our next challenge. Improved resistance to enzymatic degradation will be achieved through replacement of the ‘naked’ oligonucleotide recognition motifs with nuclease-resistant, chemically-modified analogues. Recently developed methanesulfonyl (mesyl) phosphoramidate analogues (98,99), triazole-LNA (100,101) and carbamate-LNA modified oligonucleotides (102) show a considerable improvement in their biological stability. Full or partial replacement of natural nucleotides with such modifications will protect the catalytic conjugates from enzymatic degradation, without compromising their hybridization properties towards RNA (98–102). The incorporation of multiple catalytic peptides into conjugate structures will provide additional protection from nucleases in the vicinity of their attachment points to the oligonucleotide (63,66), as will the nuclease-resistant α-anomers. Other factors that will become important in the future include the ability of such conjugates to cross biological barriers and reach their target RNA in eukaryotic cells. Arginine-rich peptides have been shown to improve cellular uptake of oligonucleotides (103), which may facilitate transport of our catalytic conjugates across biological barriers, as the first step in addressing the other, more difficult challenges including their tissue-specific delivery and intracellular trafficking. This can be achieved by similar synthetic strategies to incorporate transport functionalities into conjugate structures and to enable their selective transport across membrane barriers, both to maintain blood circulation and to traverse them into the RNA domains of target tissues.

## CONCLUSIONS

Sequence-specific chemical ribonucleases have been developed with truly-catalytic activity, improving their potential in biological knock down of pathogenic RNA.

- Bulge-loop inducing oligonucleotide conjugates with two or three catalytic peptides attached in different anomeric configuration (αα, ββ, βα, αβ or βββ) were successfully synthesized and purified by RP HPLC in good yields (30–40%) and characterized by NMR and MS, confirming the desired structures. All conjugates showed sequence-specific saturation binding, when hybridizing with the biological tRNA target, which was not affected by multiple catalytic peptides incorporated into the recognition motif.
- Multiple peptide attachment considerably increased catalytic cleavage with 90–100% degradation in 24 h by ‘*bis*’ and ‘*triple*’ conjugates compared to 70–80% by ‘*single*’ conjugates in 72 h. More dense and rigid arrangement of shorter ‘*triple*’ peptide offered no advantage over the longer more flexible ‘*bis*’ peptides. Peptides in the same configuration offered a broader attack from the same side of the RNA backbone and cut tRNA^Phe^ at five positions, whereas peptides in opposing configurations offered narrower attack from opposite sides of the RNA backbone, coinciding at only 2 positions.
- In addition to the cleavage at the induced bulge–loops, flexibly anchored catalytic peptides within the ‘*bis*’ and ‘*triple*’ conjugates also showed considerable cleaving activity outside the region directly engaged in hybridization between the tRNA^Phe^ and conjugate recognition motif, which may in the future provide potential advantage for effective demolition of long non-coding RNA or viral genomic RNA with the complex secondary and tertiary structural elements.
- RNA secondary and tertiary structure dominated reaction kinetics, with initial velocities up to 100 nM/h, with a common pathway of first cutting folds nearby the bulge loop to a threshold level of cleavage (30–40% in tRNA^Phe^) to relax this structure before cleavage within the bulge emerged and took over.
- Multiple turnover ‘*bind, cleave and leave*’ catalysis was clearly demonstrated by development of a fluorescent ribonuclease assay using a linear RNA target allowing cleavage only in the induced bulge loop, and reaction progress to be followed quantitatively with excess target.
- Complex parabolic behaviour of the cleavage kinetics was observed. A simulation model was developed to reveal the dynamic nature of available substrate and active conjugate, after complexation into unavailable and inactive complexes, particularly at large excesses of target. The underlying Michaelis-Menton kinetics then emerged, with K_m_ values of different conjugates ranging narrowly (20–24 μM), but with turnover number increasing with the number and configuration of catalytic peptides, reaching ${k_{{\rm cat}}}{\rm{\;}}0.8{\rm{\;}}{\rm h}{{\rm{\;}}^{ - 1}}$.
- Full cleavage of 10–20-fold excesses of available target demonstrated that each catalyst molecule destroyed 5–10 targets, even with only <30% of the conjugates in the active state.

When more fully activated by physiological conditions, each conjugate molecule may destroy over 30 target molecules, when a few conjugate molecules could knockdown RNA copy numbers typical biologically. Further improvement in the activation of such conjugates can be achieved *via* thorough structural design guided by the discoveries of this work.

## Supplementary Material

gkab1273_Supplemental_File
