# Supplementary material for: “Bind, cleave and leave”: multiple turnover catalysis of RNA cleavage by bulge–loop inducing supramolecular conjugates
Source: Nucleic Acids Res. 2021 Dec 30;50(2):651–73. doi: 10.1093/nar/gkab1273 (PMC8789077; doi:10.1093/nar/gkab1273)
Supplement: gkab1273_Supplemental_File [file gkab1273_supplemental_file.pdf]

"*Bind, cleave and leave*": multiple turnover catalysis of RNA cleavage *by bulge-loop inducing supramolecular conjugates*

Bahareh Amirloo, [1‡] Yaroslav Staroseletz, [2‡] Sameen Yousaf, [1] David J. Clarke,[1] Tom Brown,[3] Harmesh Aojula,[1] Marina A. Zenkova[2] and Elena V. Bichenkova[1,] *

[1] School of Health Sciences, Faculty of Biology, Medicine and Health, University of Manchester, Oxford Road, Manchester, M13 9PT, UK

[2] Institute of Chemical Biology and Fundamental Medicine SB RAS, 8 Laurentiev Avenue, 630090, Novosibirsk, RF

[3] Department of Chemistry, Chemistry Research Laboratory, University of Oxford, 12 Mansfield Road, Oxford, OX1 3TA, UK

* To whom correspondence should be addressed. Tel: + 44 161 275 8359; Fax: + 44 161 275 2481; Email: elena.v.bichenkova@manchester.ac.uk

SUPPORTING INFORMATION

Table of contents

1. **Table S1. Sequences of the unconjugated oligonucleotides and peptides** 3
2. **Figure S1. MALDI-ToF mass spectra Acetyl-[LRLRG]$_2$-CO$_2$H** 3
3. **Figure S2. ^{1}H-NMR spectra of the Acetyl-[LRLRG]$_2$-CO$_2$H peptide** 4
4. **Figure S3. MALDI-ToF mass spectra Acetyl-[LR]$_3$G-CO$_2$H** 4
5. **Figure S4. ^{1}H-NMR spectra of the Acetyl-[LR]$_3$G-CO$_2$H peptide** 5
6. **Figure S5. General synthetic route of *"bis"* and *"triple"* conjugation** 5
7. **Figure S6. Shift in RP-HPLC purification chromatogram for *"bis"* and *"triple"* conjugates** 6
8. **Figure S7. MALDI-ToF mass spectra of *"bis"* and *"triple"* peptidyl-oligonucleotide conjugates** 6
9. **Figure S8. ^{1}H NMR spectra of the bulge-loop inducing *"bis"* and *"triple"* conjugates** 7
10. **Figure S9. ^{1}H NMR comparison of *"single"*, *"bis"* and *"triple"* conjugates** 8
11. **Figure S10. Lack of stable interactions of conjugates with tRNA^{Phe} outside the target region** 9
12. **Figure S11. Gel-shift analysis of hybridisation of *"bis"* and *"triple"* conjugates with 3'-FITC-tRNA^{Phe}** 10
13. **Figure S12. Cleavage of 3'-FITC-tRNA^{Phe} at 10-fold excess of *"bis"* and *"triple"* conjugates over target** 11

‡ These authors contributed equally to this work.

14. Figure S13. Cleavage of 3'-FITC-tRNA^{Phe} at 5-fold excess of *"bis"* and *"triple"* conjugates over target **12**

15. Figure S14. Representative examples of cleavage products of 3'-FITC-tRNA^{Phe} *in vitro* transcript **13**

16. Figure S15. Cleavage from within and outside the bulge region by excess conjugate **14**

17. Figure S16. Progress of target decline and reaction velocities within and outside the bulge **15**

18. Figure S17. Representative examples of hybridisation to labelled linear target by conjugates **16**

19. Figure S18. Absence of significant non-specific cleavage of labelled linear F-Q-RNA target **16**

20. Figure S19. Examples of possible hairpin secondary structures of conjugates and labelled linear target F-Q-RNA **17**

21. Model of target and catalytic conjugate interactions to explain atypical kinetic observations **18**

21.1. Model **19**

21.2. Calculation of fractions expected from the model **20**

21.3. Conjugate activation and inactivation model **23**

21.4. Competitive and uncompetitive inhibition **23**

22. References for the Supporting Information **25**

1. Sequences of the unconjugated oligonucleotides and peptides

Table S1. Sequences and millimolar extinction coefficients values for peptides, unconjugated oligonucleotide and labelled linear target used for this study.

Name	Sequence (5' to 3')	E_{260} ($mM^{-1}\cdot cm^{-1}$)[a]
**Ac-[LR]$_3$G-CO_2H**	Ac-Leu-Arg-Leu-Arg-Leu-Arg-Gly	n/a
**Ac-[LRLRG]$_2$-CO_2H**	Ac-Leu-Arg-Leu-Arg-Gly-Leu-Arg-Leu-Arg-Gly	n/a
**BC5-L-ββ**	TGGTGCGAATT- **dR^{β}-dR^{β}** -GATCGAACACAGGAC[b]	269.1
**BC5-L-αα**	TGGTGCGAATT- **dR^{α}-dR^{α}**-GATCGAACACAGGAC[b]	269.1
**BC5-L-αβ**	TGGTGCGAATT- **dR^{α}-dR^{β}** –GATCGAACACAGGAC[b]	269.1
**BC5-L-βα**	TGGTGCGAATT- **dR^{β}-dR^{α}** –GATCGAACACAGGAC[b]	269.1
**BC5-L-βββ**	TGGTGCGAATT- **dR^{β}-dR^{β}-dR^{β}** GATCGAACACAGGAC[b]	269.1
**Fluor-labelled linear target**	GTCCTGTGTTCGA**T^F**C**$C_r$$A_r$$C_r$$A_r$$G_r$**AA**$T^Q$**TCGCACCA	337.5

a) **E_{260} denotes the millimolar extinction coefficient**

b) **dR denotes abasic nucleotide incorporated in oligonucleotide sequence**

2. Figure S1. MALDI-ToF mass spectra Acetyl-[LRLRG]$_2$-CO_2H

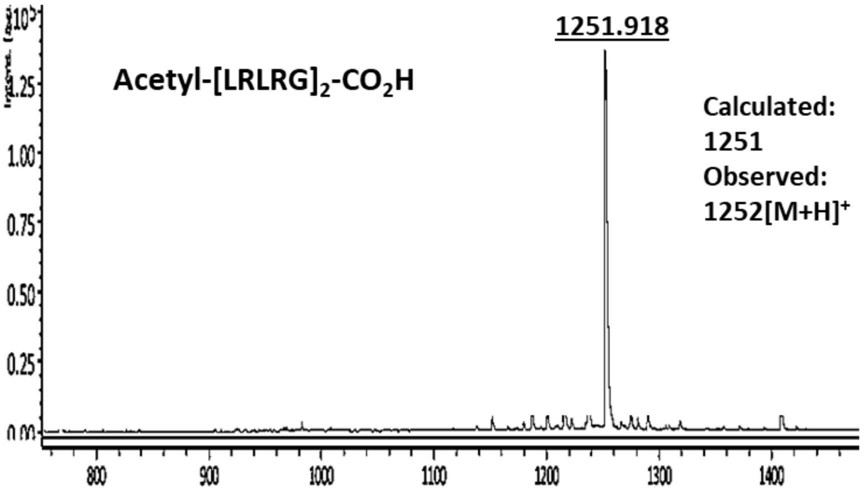

Supplementary Figure S1. MALDI-TOF spectra of peptide Acetyl-[LRLRG]$_2$-CO_2H. Spectra were recorded using a Bruker Daltonics Ultraflex TOF/TOF mass spectrometer.

3. Figure S2. ^{1}H-NMR spectra of the Acetyl-[LRLRG]$_2$-CO_2H peptide

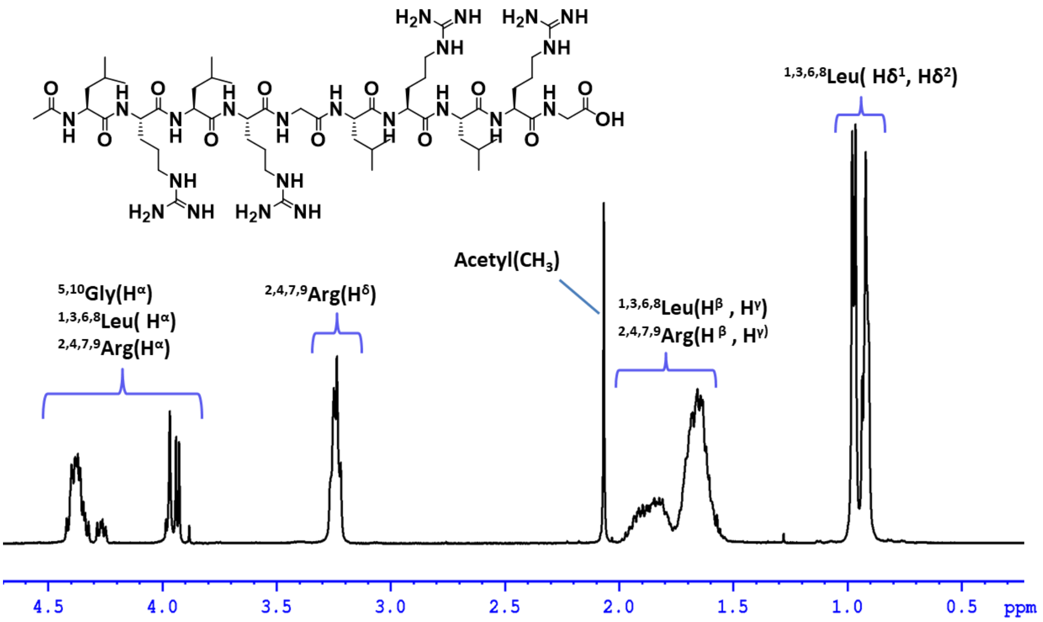

Supplementary Figure S2. ^{1}H NMR spectrum for Acetyl-[LRLRG]$_2$-CO_2H peptide. The spectrum was recorded in D_2O at 25 °C using 400 MHz NMR spectrometer (Bruker Avance II+ 400).

4. Figure S3. MALDI-ToF mass spectra Acetyl-[LR]$_3$G-CO_2H

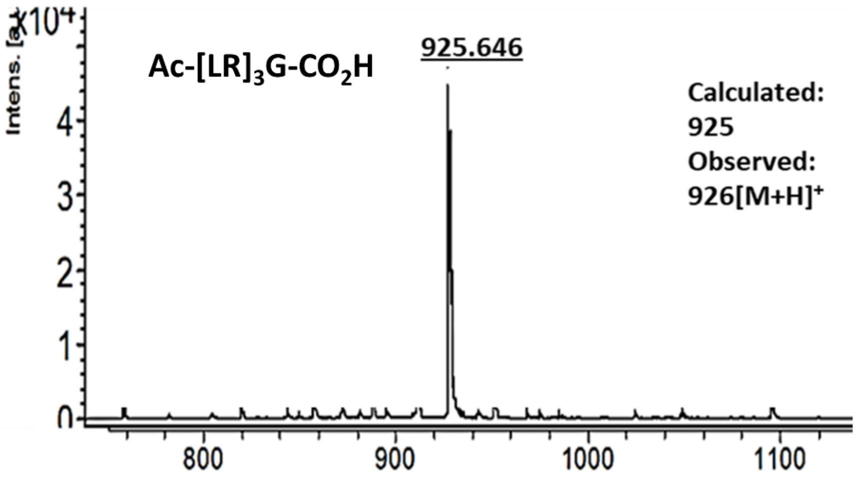

Supplementary Figure S3. MALDI-TOF spectra of peptide Acetyl-[LR]$_3$G-CO_2H Spectra were recorded using a Bruker Daltonics Ultraflex TOF/TOF mass spectrometer.

5. Figure S4. ^{1}H-NMR spectra of the Acetyl-[LR]$_3$G-CO$_2$H peptide

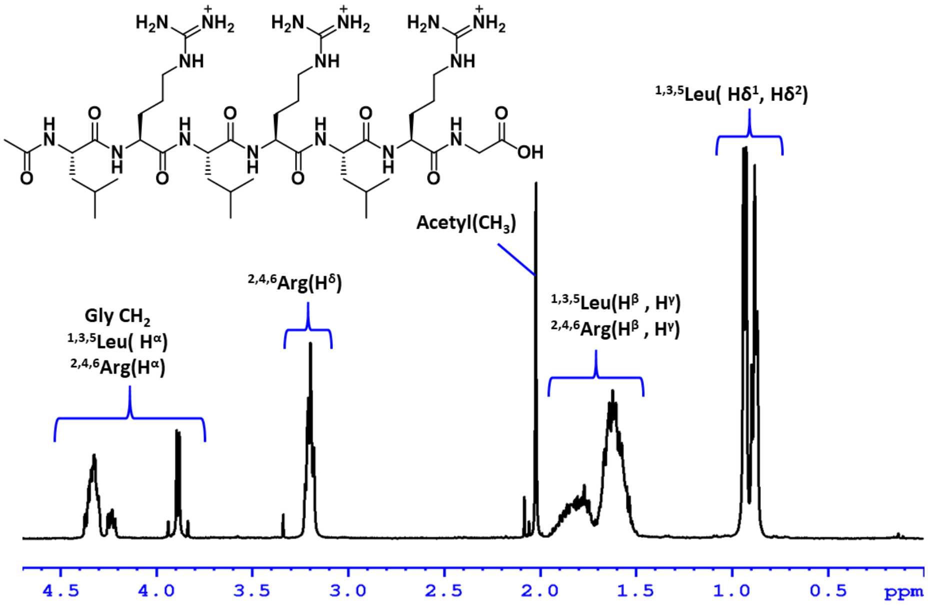

Supplementary Figure S4. ^{1}H NMR spectrum for Acetyl-[LR]$_3$G-CO$_2$H peptide recorded in D_2O at 400 MHz.

6. Figure S5. General synthetic route of *"bis"* and *"triple"* conjugation

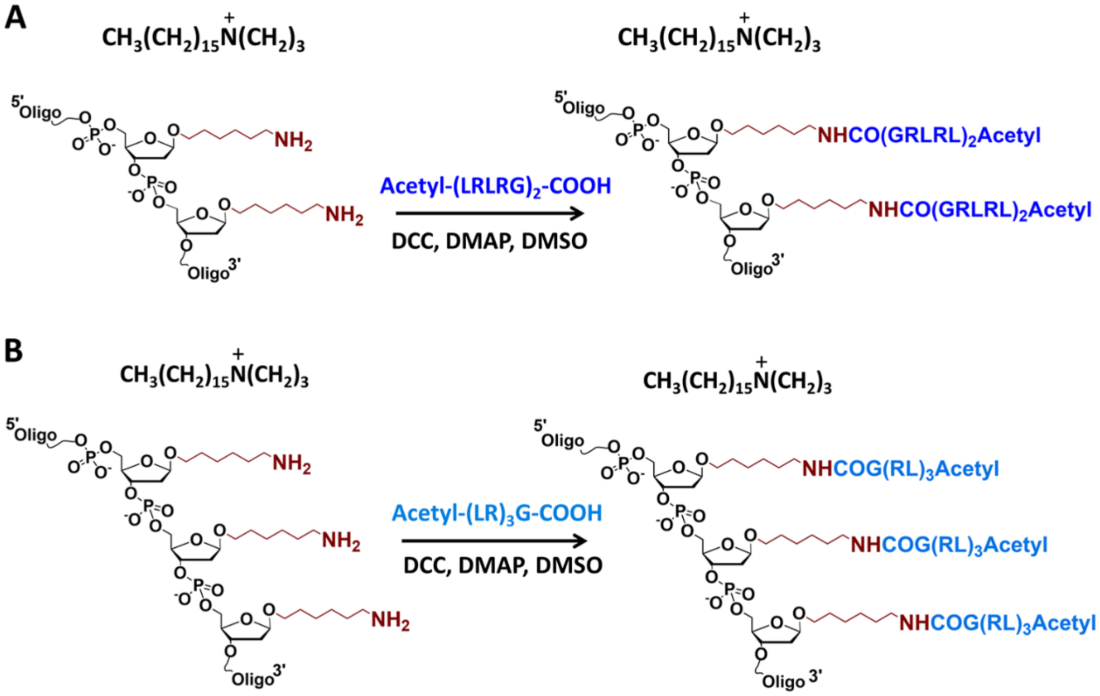

Supplementary Figure S5. General synthetic route of conjugation. (**A**) Synthetic route for the production of *"bis"* conjugates, demonstrated by the example of BC5-L-ββ conjugate synthesis, when the β configuration was used for aminohexyl linker attachment at C1' position of abasic nucleotides. A similar synthetic route was used for the synthesis of BC5-L-αα, BC5-L-βα and BC5-L-αβ conjugates, when the α configuration was also used for aminohexyl linker attachment at the C1' position of abasic nucleotides. (**B**) Synthetic route for the *"triple"* conjugate BC5-L-βββ. Antisense oligonucleotide containing two or three internal abasic nucleotide either in α or β configuration was conjugated to catalytic peptide *via* aminohexyl linker. To avoid peptide self-conjugation and cyclisation during the amide coupling reaction, peptide was acetylated at the N-terminus.

7. Figure S6. Shift in RP-HPLC elution of *“bis”* and *“triple”* conjugates

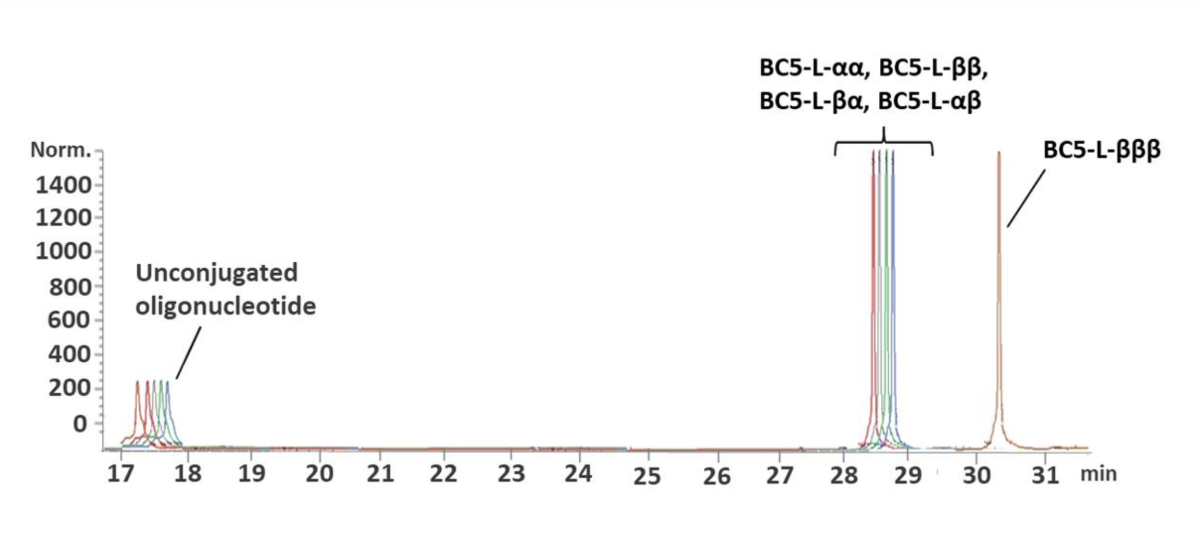

Supplementary Figure S6. Overlay of the RP-HPLC purification chromatogram of BC5-L-αα (red), BC5-L-ββ (grey), BC5-L-βα (green), BC5-L-αβ (blue) and BC5-L-βββ (orange), showing the shift in retention time from unconjugated oligonucleotide to *“bis”* and *“triple”* conjugates.

8. Figure S7. MALDI-ToF mass spectra of *“bis”* and *“triple”* peptidyl-oligonucleotide conjugates

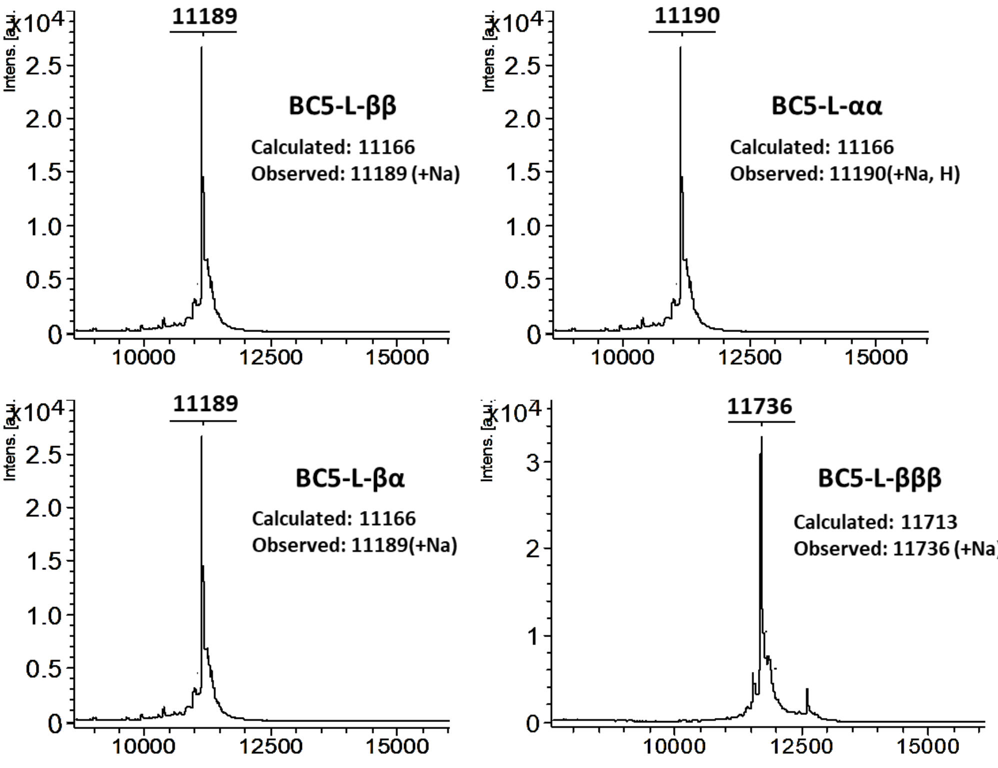

Supplementary Figure S7. MALDI-TOF spectra of *“bis”* and *“triple”* peptidyl-oligonucleotide conjugates. Spectra were recorded using 0.7 M 3-hydroxy picolinic acid matrix (97 mg/mL, with 0.07 M ammonium citrate, 16 mg/mL in 50:50 ACN:H_2O) on a Bruker Daltonics Ultraflex ToF/ToF mass spectrometer.

9. Figure S8. ^{1}H NMR spectra of the bulge-loop inducing *“bis”* and *“triple”* conjugates

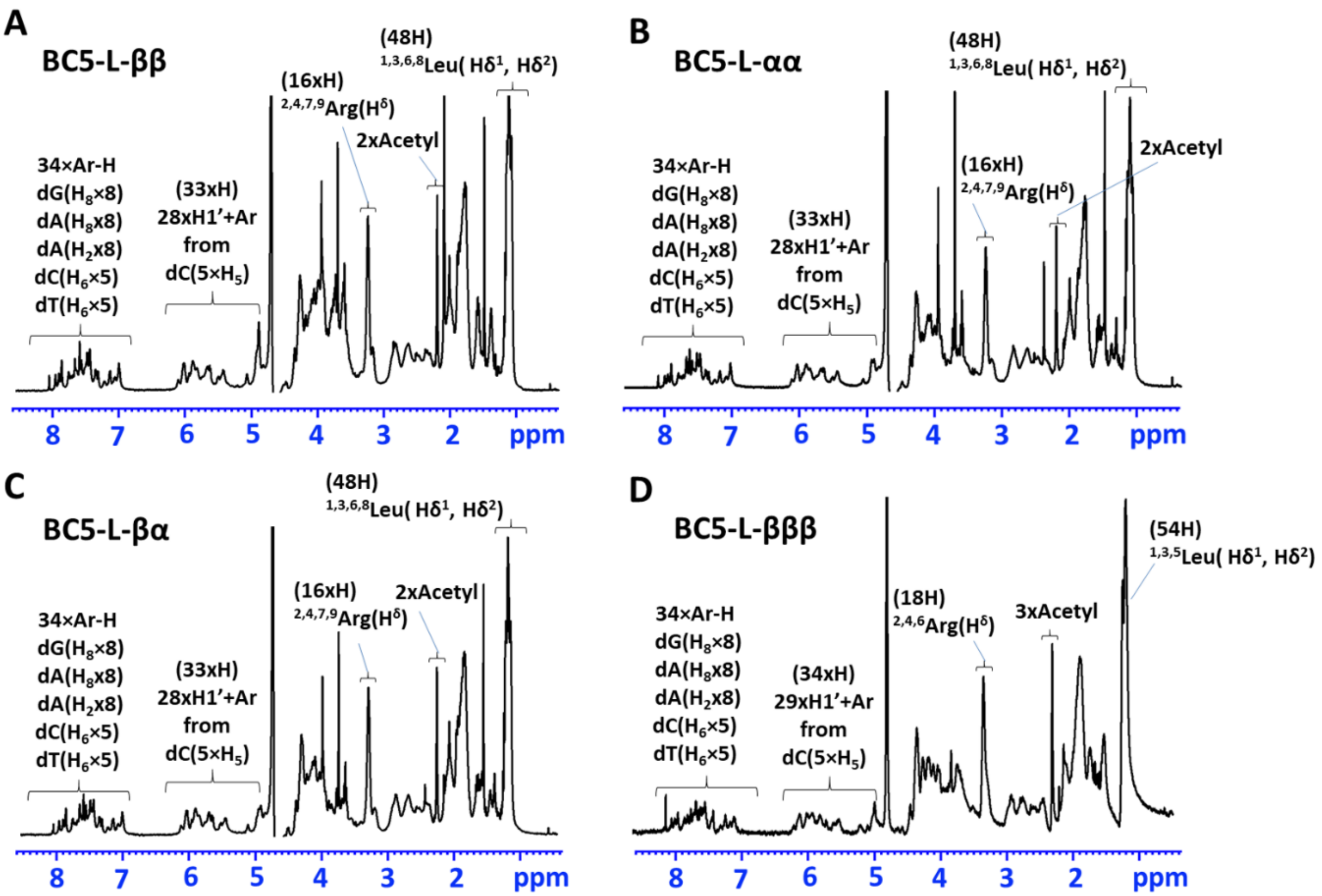

Supplementary Figure S8. ^{1}H NMR spectra (400 MHz, Bruker Avance IIþ 400) of *“bis”* and *“triple”* conjugates indicating the prominent chemical shift of protons from oligonucleotides, peptide, aminohexyl linker and acetyl protecting group. In each spectrum, the breakdown of proton assignment for each region as well as integral intensity has been indicated. H3’ region has not been assigned because water suppression prohibits full assignment.

10. Figure S9. ^{1}H NMR comparison of *"single"*, *"bis"* and *"triple"* conjugates

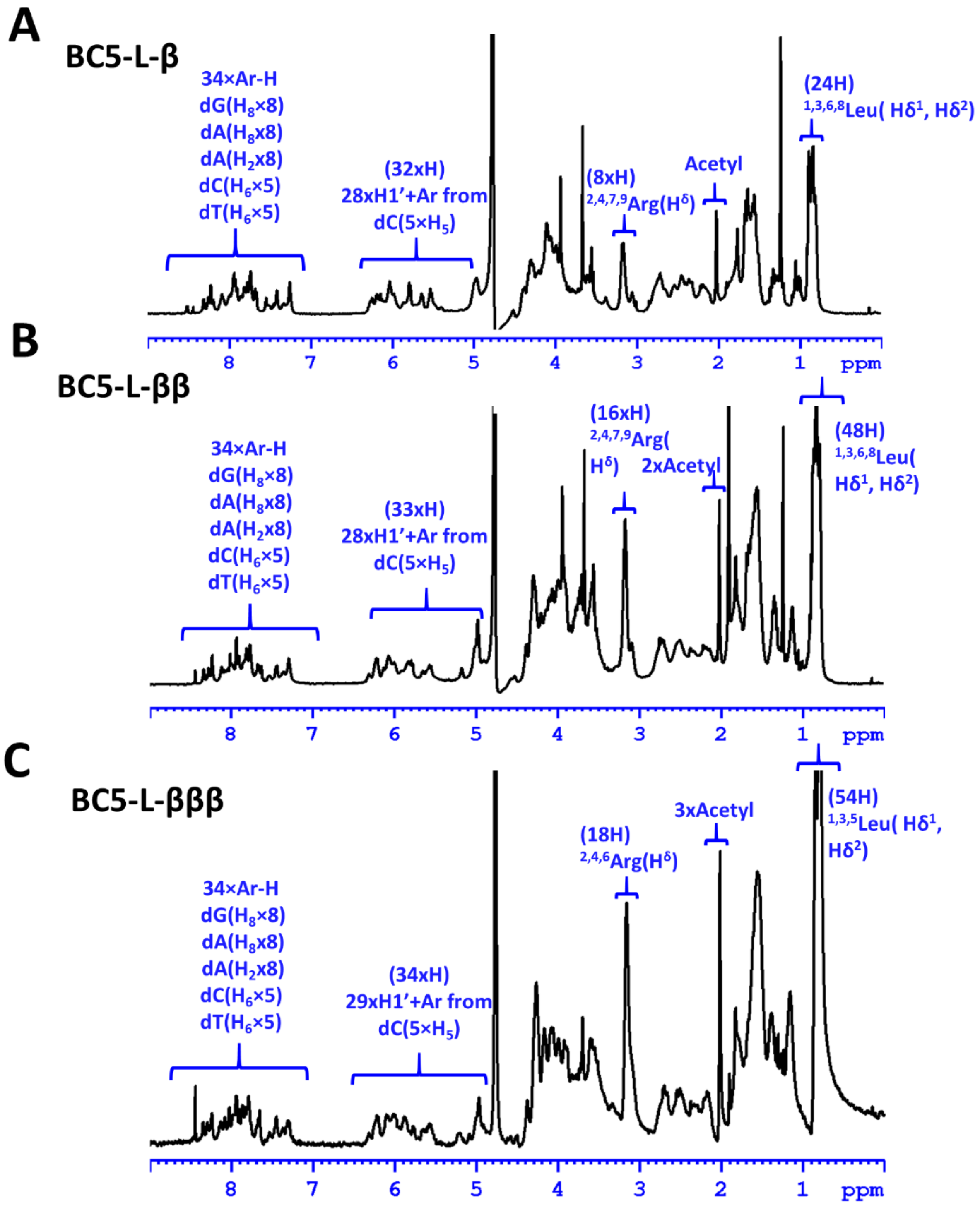

Supplementary Figure S9. ^{1}H NMR spectra (400 MHz, Bruker Avance IIþ 400) of BC5-L-β *"single"* conjugate **(A)** BC5-L-ββ *"bis"* conjugate **(B)** and BC5-L- βββ *"triple"* conjugate **(C)**. The prominent chemical shifts corresponding to oligonucleotides, peptide, aminohexyl linker and acetyl protecting group can be clearly seen. Careful integration of the oligonucleotide aromatic region as well as H1' sugar ring protons confirmed 1:1, 2:1 and 3:1 stoichiometric ratio of peptide to oligonucleotide in *"single"*, *"bis"* and *"triple"* conjugates.

11. Figure S10. Lack of stable interactions of conjugates with tRNA^{Phe} outside the target region

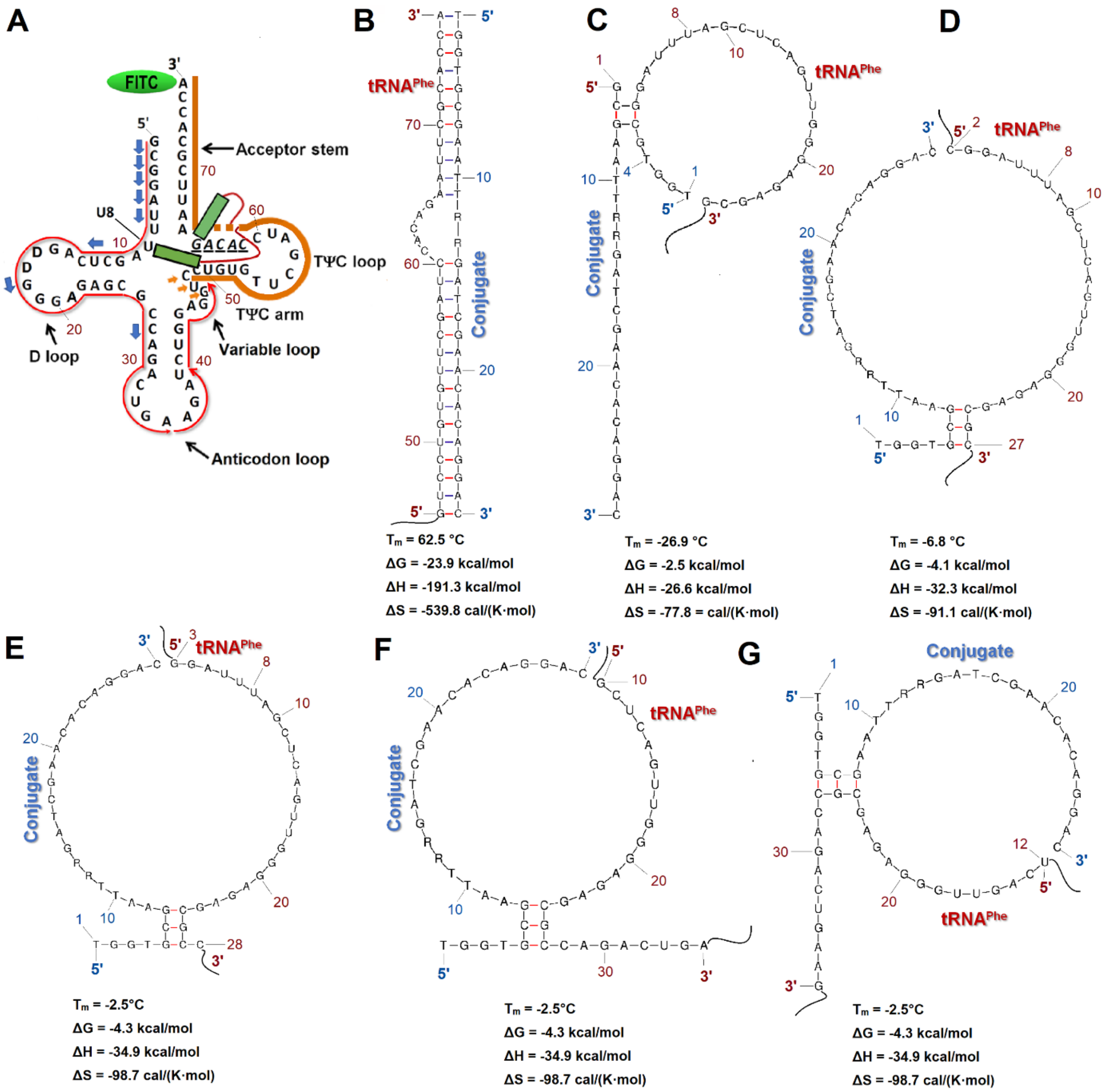

Supplementary Figure S10. **Lack of stable interactions of conjugates with tRNA^{Phe} outside the target region.** (**A**) Secondary structure of 3'-FITC-tRNA^{Phe} in vitro transcript. The recognition motifs of the bulge-loop inducing conjugate (shown here by orange line and arrows) is hybridised with the acceptor stem, TΨC arm and variable loop of 3'-FITC-tRNA^{Phe}. Dotted orange lines show variable regions containing abasic nucleotides bearing the catalytic peptides (green). The bulge-loop forming target site is underlined in italics. (**B**) Predicted complex between the conjugate recognition motif and the target tRNA^{Phe} region involving 3'-acceptor stem, TΨC-arm and variable loop, which are located in the 3'-part of the of tRNA^{Phe}. (**C-G**) Representative examples of the predicted complexes between the conjugate recognition motif and various 26-nt RNA regions from 5'-part of the tRNA^{Phe}. Secondary structures and corresponding thermodynamics parameters (ΔG, ΔH, ΔS and Tm) were calculated using DINAMelt Server (www.unafold.org). The conjugate recognition sequences (TGGTGCGAATT-dR-dR-GATCGAACACAGGAC and TGGTGCGAATT-dR-dR-dR-GATC-GAACACAGGAC) were screened against every possible 26-nt section of the 5'-part of tRNA^{Phe} by "sliding" it along this sequence with 1-nt incremental step to fully cover the region between 5'-terminal ^{1}G residue and ^{50}C nucleotide located in the beginning of TΨC-arm, as indicated by red trace and blue arrows. Labelling is shown in red for RNA and in blue for conjugate.

12. **Figure S11. Gel-shift analysis of hybridisation of *"bis"* and *"triple"* conjugates with 3'-FITC-tRNA^{Phe}**

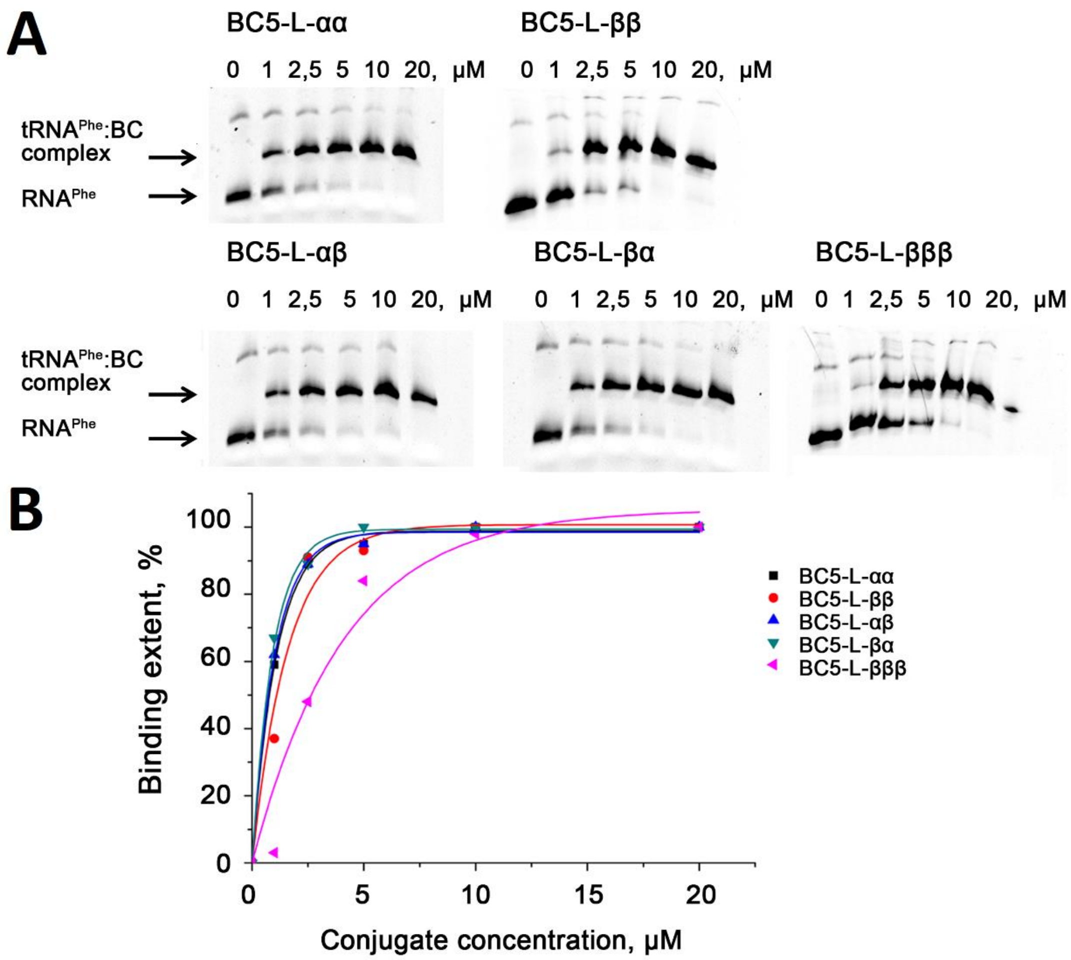

Supplementary Figure S11. Gel-shift analysis of hybridisation of *"bis"* and *"triple"* bulge-loop inducing conjugates with 3'-FITC-tRNA^{Phe} (1 μM). (**A**) Representative images of PAGE showing binding of conjugates to target tRNA^{Phe}. The type of conjugate and concentration (0-20 μM) are indicated at the top of each image. (**B**) Secondary plot of data shown in (**A**).

13. **Figure S12. Cleavage of 3'-FITC-tRNA^{Phe} at 10-fold excess of *"bis"* and *"triple"* conjugates over target**

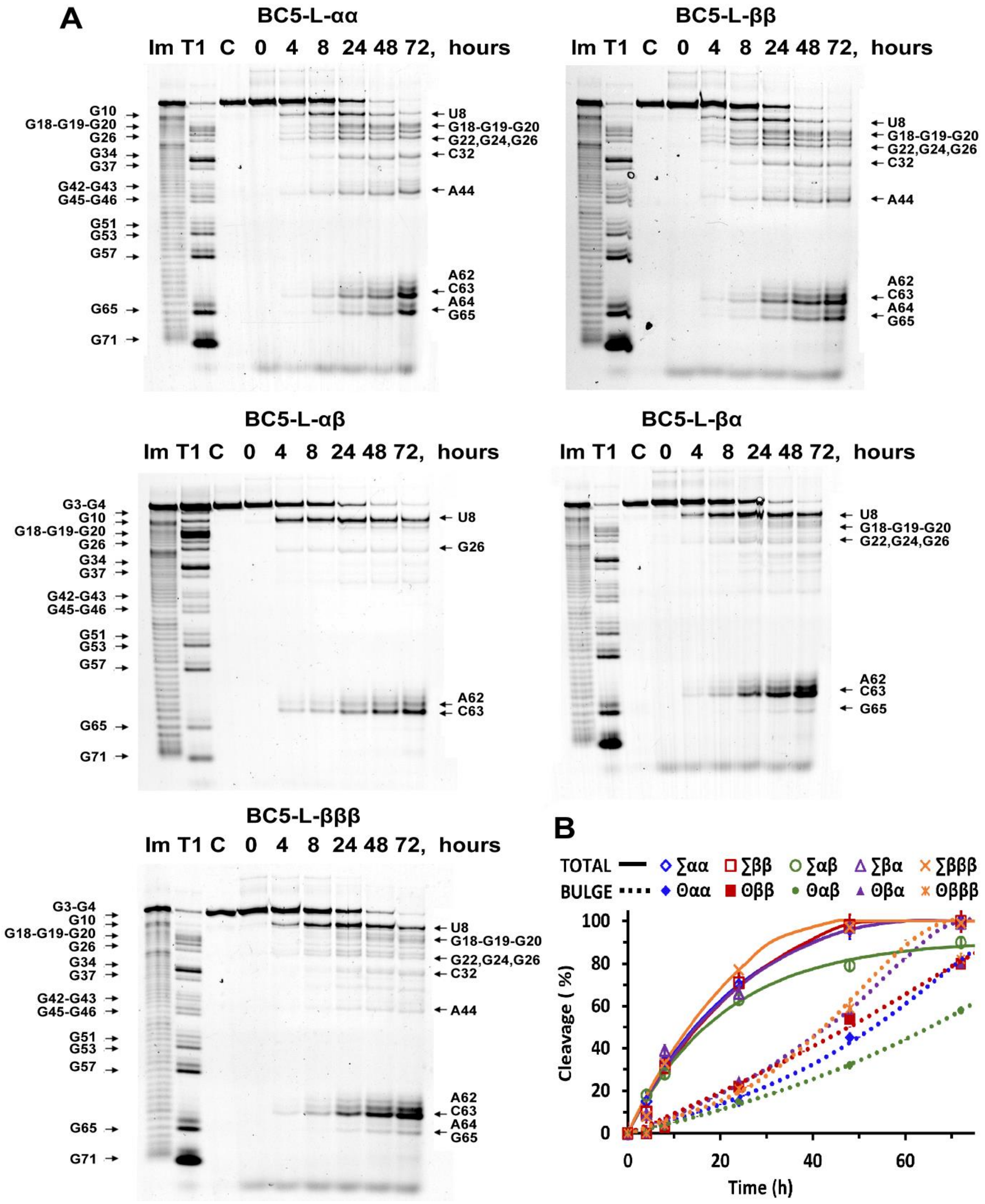

Supplementary Figure S12. Cleavage of 3'-FITC-tRNA^{Phe} with *"bis"* and *"triple"* conjugates at 10-fold excess of conjugate over target. **(A)** Representative images of 12% PAAM/8M urea gel after electrophoresis of 3'-FITC-tRNA^{Phe} (1 µM) cleavage products with *"bis"* and *"triple"* conjugates (10 µM) as a function of incubation time. Lanes T1 and Im represent partial 3'-FITC- tRNA^{Phe} digestion with RNase T1 and 2M imidazole, respectively. In lane C, 3'-FITC- tRNA^{Phe} was incubated without conjugates for 72 hours. Positions of RNA cleavage by RNase T1 and conjugates are shown on left and right respectively. **(B)** Progress of total (continuous curve) and bulge (dotted curve) cleavage (%) taken from **(A)**.

14. **Figure S13. Cleavage of 3'-FITC-tRNA^{Phe} at 5-fold excess of *"bis"* and *"triple"* conjugates over target**

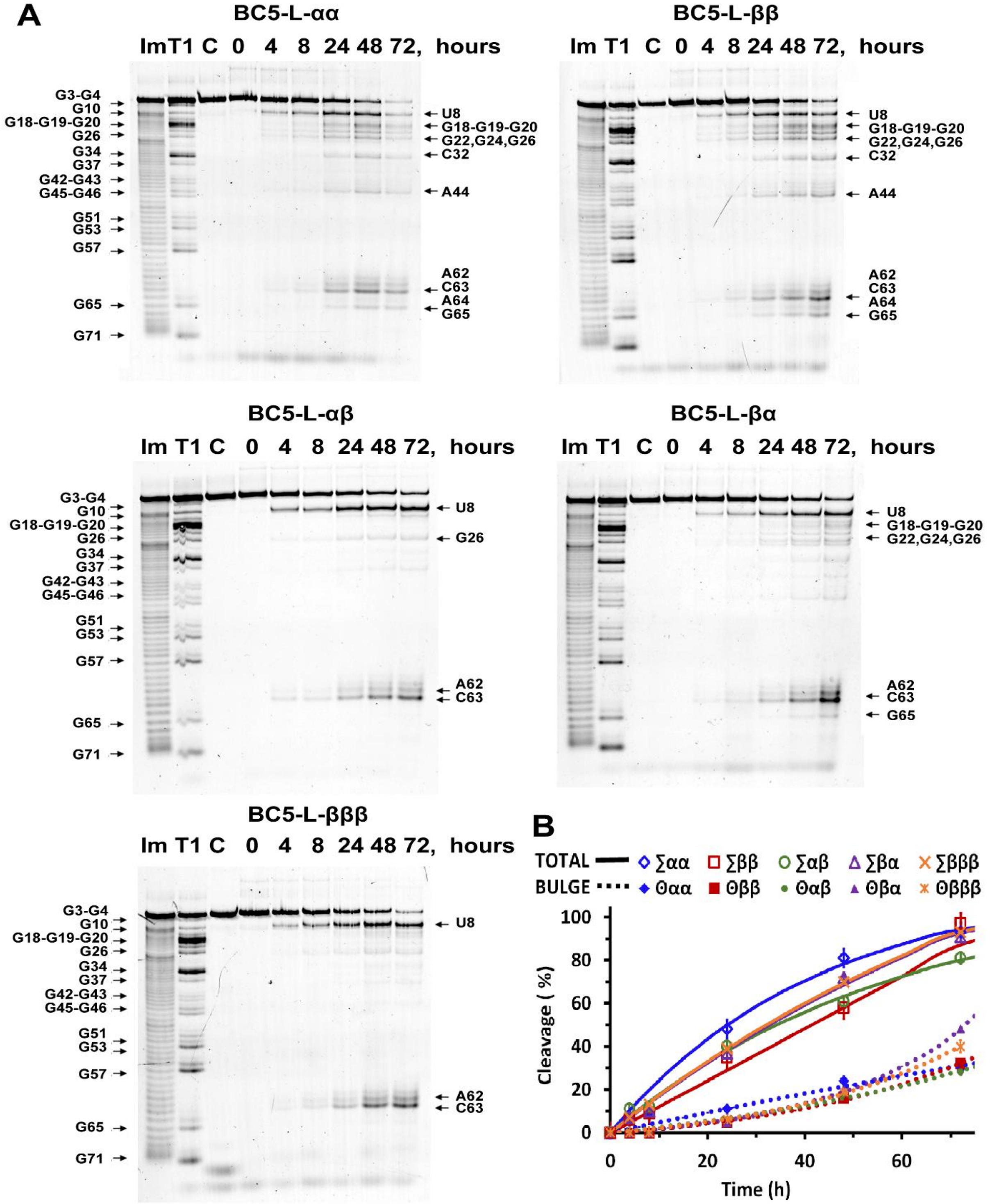

Supplementary Figure S13. Cleavage of 3'-FITC-tRNA^{Phe} with *"bis"* and *"triple"* conjugates at 5-fold excess of conjugate over target. **(A)** Representative images of 12% PAAM/8M urea gel after electrophoresis of 3'-FITC-tRNA^{Phe} (1 μM) cleavage products with *"bis"* and *"triple"* conjugates (5 μM) as a function of incubation time. Lanes T1 and Im represent partial 3'-FITC- tRNA^{Phe} digestion with RNase T1 and 2M imidazole, respectively. In lane C, 3'-FITC- tRNA^{Phe} was incubated without conjugates for 72 hours. Positions of RNA cleavage by RNase T1 and conjugates are shown on left and right respectively. **(B)** Progress of total (continuous curves) and bulge (dotted curves) cleavage (%) taken from **(A)**.

15. Figure S14. Representative examples of cleavage products of 3’-FITC-tRNA^{Phe} *in vitro* transcript

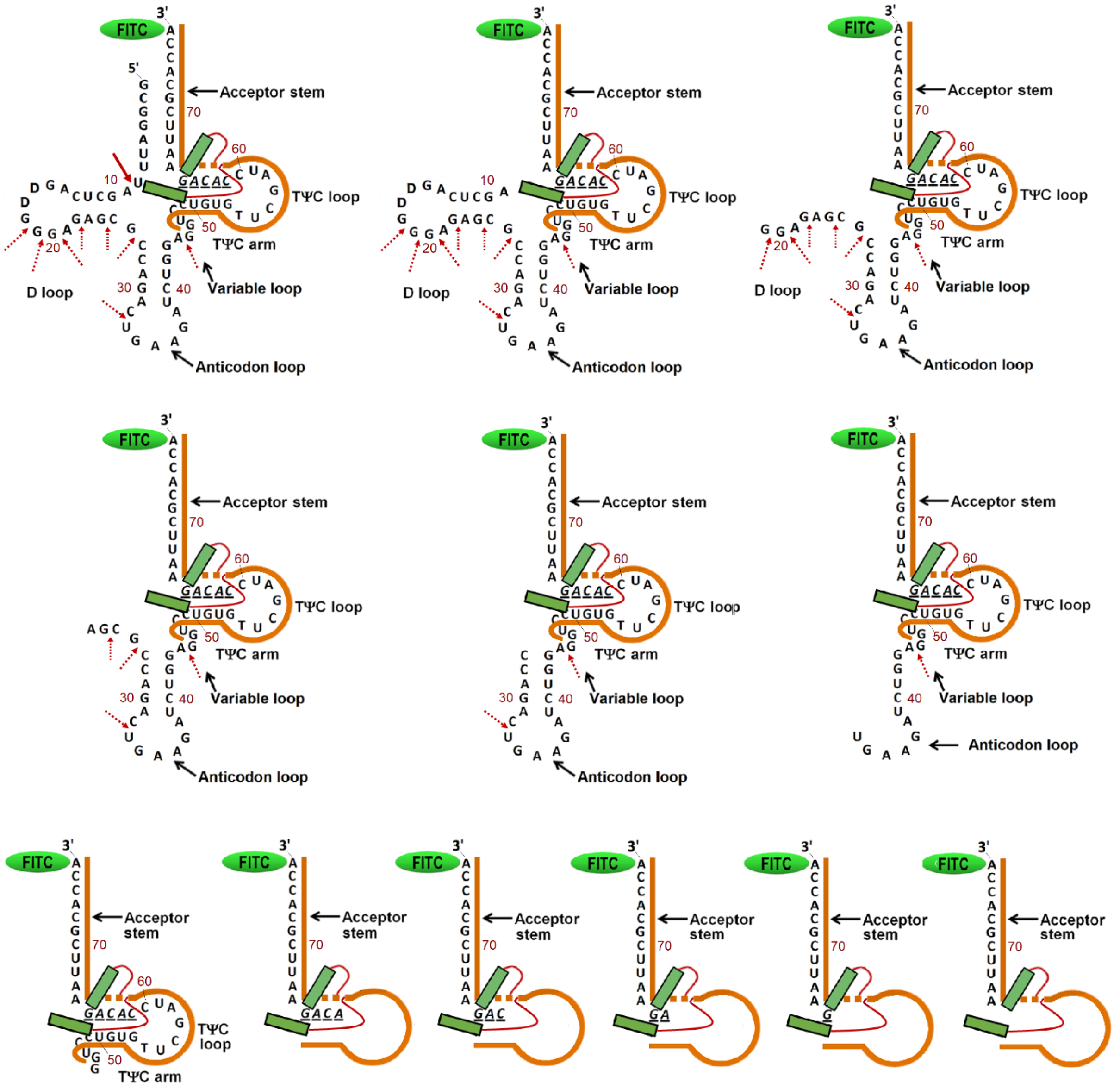

Supplementary Figure S14. Secondary structure of 3’-FITC-tRNA^{Phe} *in vitro* transcript (top left image), along with representative examples of its cleavage products upon treatment with *“bis”* or *“triple”* conjugates detected by electrophoretic analysis (see **Figure 3**). The recognition motifs of the bulge-loop inducing conjugate (shown here by orange line) is hybridised with the acceptor stem, TΨC arm and variable loop of 3’-FITC-tRNA^{Phe}. Dotted orange lines show variable regions containing abasic nucleotides bearing the catalytic peptide (green). The bulge-loop forming target site is underlined in italics.

16. Figure S15. Cleavage from within and outside the bulge region by excess conjugate

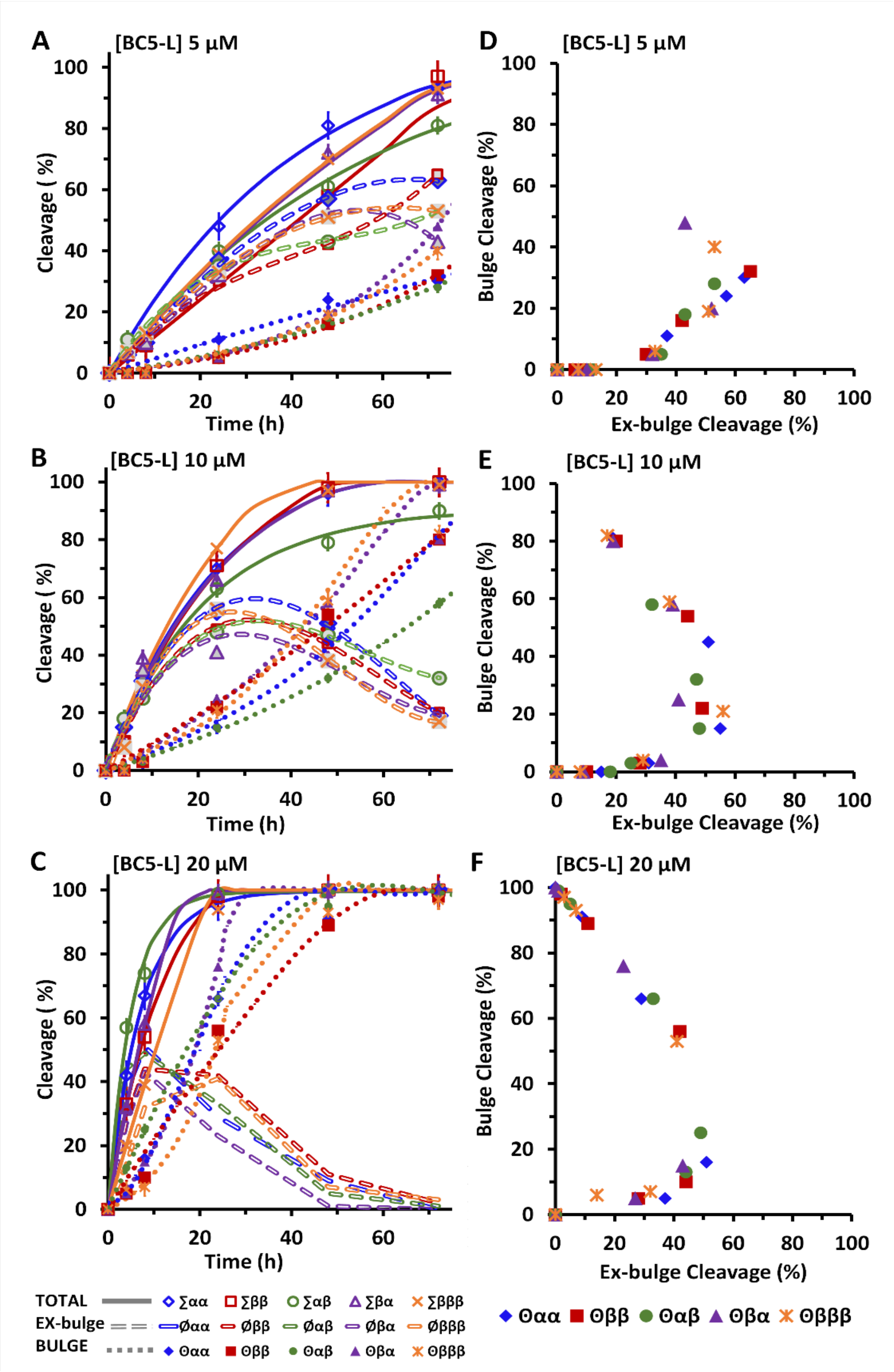

Supplementary Figure S15. Cleavage from within and outside the bulge region by excess conjugate. **Left column:** Contributions to cleavage (TOTAL (∑) continuous curves) from cleavage within the bulge region (BULGE (Θ), dotted curves) and outside the bulge region (EX-bulge (Ø), dashed curves) at (**A**) 5-fold, (**B**) 10-fold and (**C**) 20-fold molar excesses of conjugate over 3'-FITC-tRNA^{Phe} (1 µM). **Right column:** Progress of the common pathway of cleavage with a 30-40% threshold of cleavage outside the bulge before cleavage within the bulge emerged and took over with increasing conjugate excesses (**D**) 5-fold, (**E**) 10-fold and (**F**) 20-fold. Secondary plots taken from data in **Figures 3, S12** and **S13**.

17. Figure S16. Progress of target decline and reaction velocities within and outside the bulge

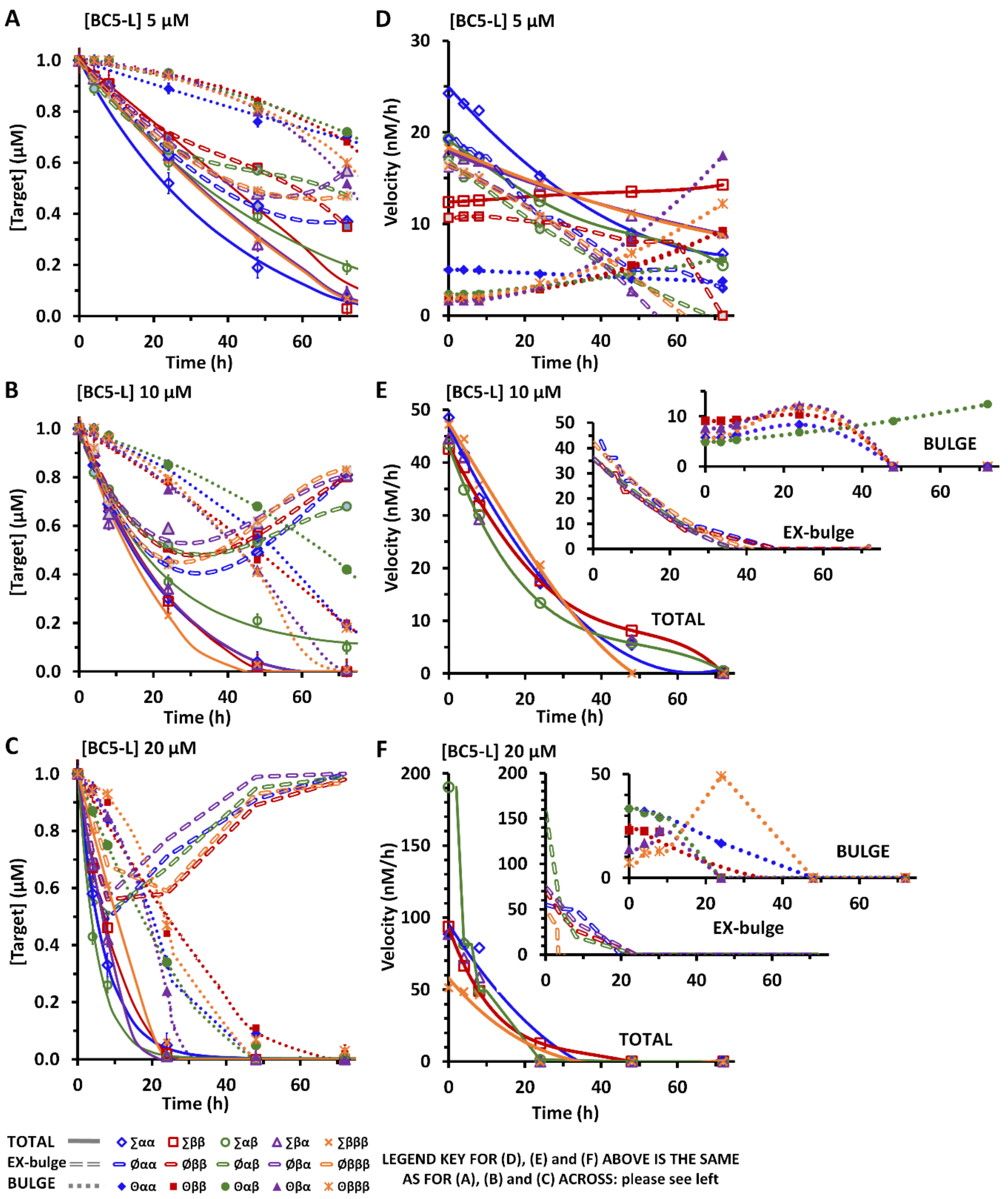

Supplementary Figure S16. Progress of target decline and change in reaction velocities within and outside the bulge region**. Left column:** Decline in total target concentration (TOTAL (∑), continuous curves) and the contributions from cleavage within the bulge region (Bulge (Ѳ), dotted curves) and from outside the bulge region (EX-bulge (Ø), dashed curves) at (**A**) 5-fold, (**B**) 10-fold and (**C**) 20-fold molar excesses of conjugate over 3'-FITC-tRNA^{Phe} (1 µM) target. **Right column:** Change in cleavage reaction velocities (TOTAL (∑) = Ex-bulge (Ø) + Bulge (Ѳ)) for the same molar excesses of conjugate (**D**) 5-fold, (**E**) 10-fold and (**F**) 20-fold. Non-linear curve fitting of $[Target] = \frac{v_0}{\eta}(1 - e^{-\eta t})$ (1, 2) estimated non-linearities η and initial velocities v_0 for reaction time (t), which allowed estimation of reaction velocities as $v = \frac{d[Target]}{dt} = v_0 - \eta[Target]$ (1). Secondary plots taken from data in **Figures 3, S12 and S13**.

18. Figure S17. Representative examples of hybridisation to labelled linear target by conjugates

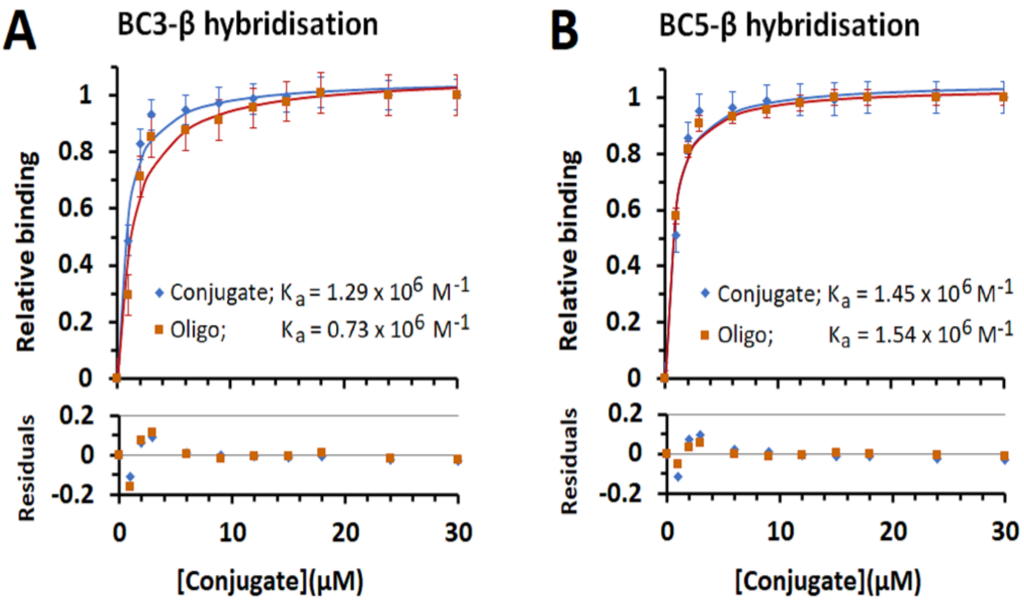

Supplementary Figure S17. Representative examples of hybridisation to labelled linear target by conjugates: (**A**) BC3-β and (**B**) BC5-β. Fluorescently-labelled **F**-**Q**-RNA target (1μM) in Tris-buffer (50 mM Tris–HCl pH 7.0, 0.2 mM KCl, and 1 mM EDTA) was incubated for 30 minutes either with one of the conjugates (here, with BC3-β or BC5-β, blue diamond symbols) or with the corresponding unconjugated oligonucleotide (orange square symbols) at concentrations ranging from 0 to 30 μM. Relative quenching of fluorescence (at λ_{em} = 522 nm following excitation at λ_{ex} = 496 nm) is expressed as relative binding (hybridisation) of the conjugate or the corresponding unconjugated oligonucleotide to the **F**-**Q**-RNA target. Association constant K_a values are reciprocals of dissociation constant K_d values estimated by non-linear numerical fitting of the hyperbolic curves (continuous traces) by minimising the sums of squares of residual difference between observed and fitted data sets, where error bars represent the standard errors of their regression analysis at the 95% confidence limit.

19. Figure S18. Absence of significant non-specific cleavage of labelled linear F-Q-RNA target

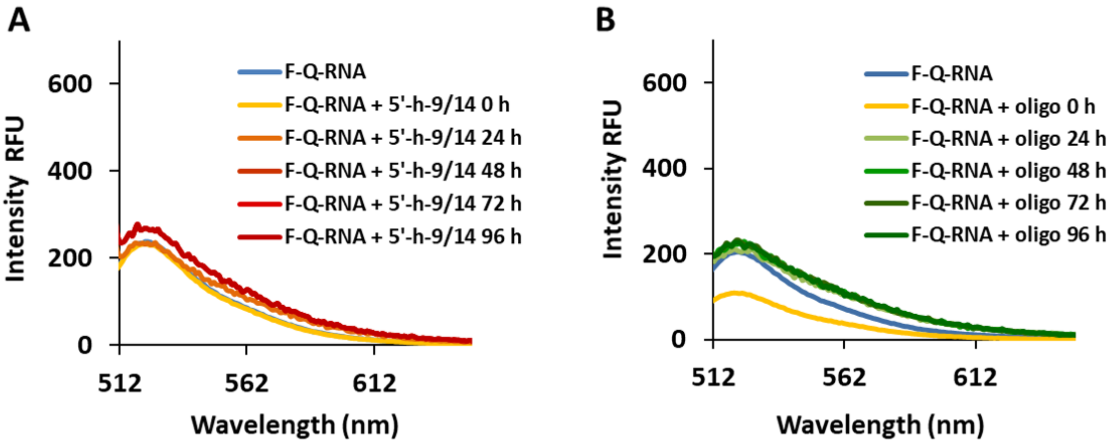

Supplementary Figure S18. Fluorescence spectra of **F**-**Q**-RNA (1 μM) incubated with a scrambled peptidyl-oligonucleotide 5'-h-9/14 (20 μM), lacking complementarity with the oligonucleotide sequence of the RNA target (**A**), and with an unconjugated oligonucleotide sequence (20 μM) (**B**) at 37°C. Lack in fluorescence increase over 96 hours in both cases indicated absence of spontaneous cleavage.

20. Figure S19. Examples of possible hairpin secondary structures of conjugates and labelled linear target F-Q-RNA

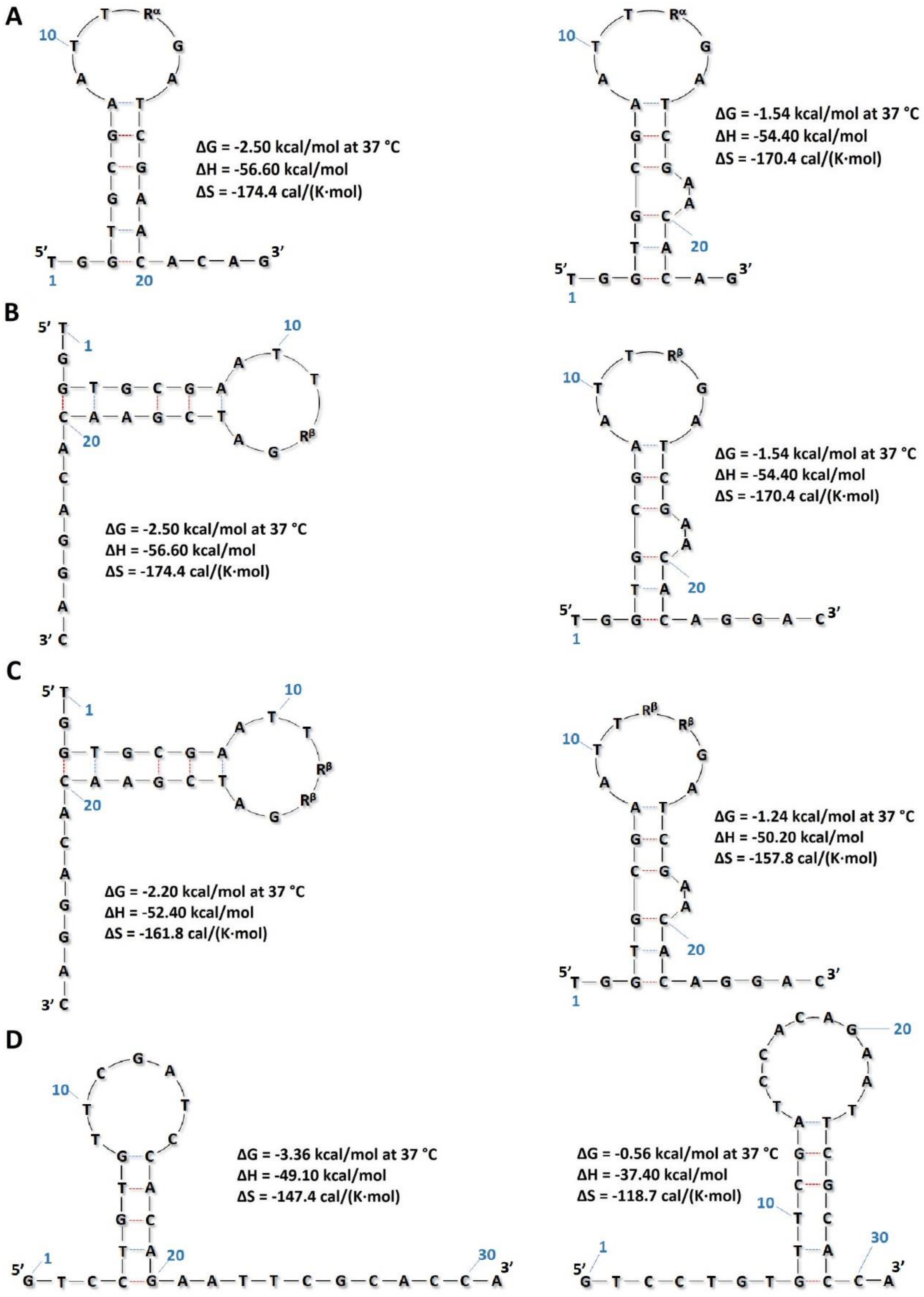

Supplementary Figure S19. Examples of *intra*molecular hairpin secondary structures of conjugates and labelled linear target F-Q-RNA. The most stable folded structures of BC5-α (**A**), BC5-L-β (**B**) and BC5-L-ββ (**C**) conjugates and **F**-**Q**-RNA target (**D**), alone with the thermodynamic parameters of folding, calculated using UNAFold web server (www.unafold.org).

21. Model of target and catalytic conjugate interactions to explain atypical kinetics observed

Atypical kinetics of **F-Q**-RNA substrate cleavage by catalytic conjugates BC5-L-α, BC5-L-β and BC5-L-ββ observed under multiple turnover conditions (see **Figure 7** and **Table 2**) could not be explained by Michaelis-Menten enzyme catalysis. The plotted correlation between the measured initial reaction velocity against target substrate concentration showed significant deviation from any hyperbolic curve consistent with Michaelis-Menten reaction model. New kinetic principles might need to be elucidated with a novel catalyst. We first consider whether side interactions of the substrate, catalyst and product can explain these behaviours within an otherwise Michaelis-Menten model, where the catalytic complexation of available **F-Q**-RNA substrate to the conjugate is considered to be in competition with weaker self-associations (**Supplementary Figure S19**), which both inactivate the catalytic conjugate and sequester substrate into unavailable complexes.

Indeed, when substrate depletion was considered, by subtraction of the observed increase in product, the decline in substrate appeared to be considerable during the progress of the reactions, at least for substrate excesses greater than 2-fold (**Supplementary Figure 20** (**A-C**)). Such an extent of substrate depletion would be expected to exert a greater effect on the decline in velocity during the course of the reactions, which was not apparent in the progress curves in their relatively small curvatures (**Figure 7** (**A-C**)) and non-linearity factors (**Figure 7** (**D-F**)).

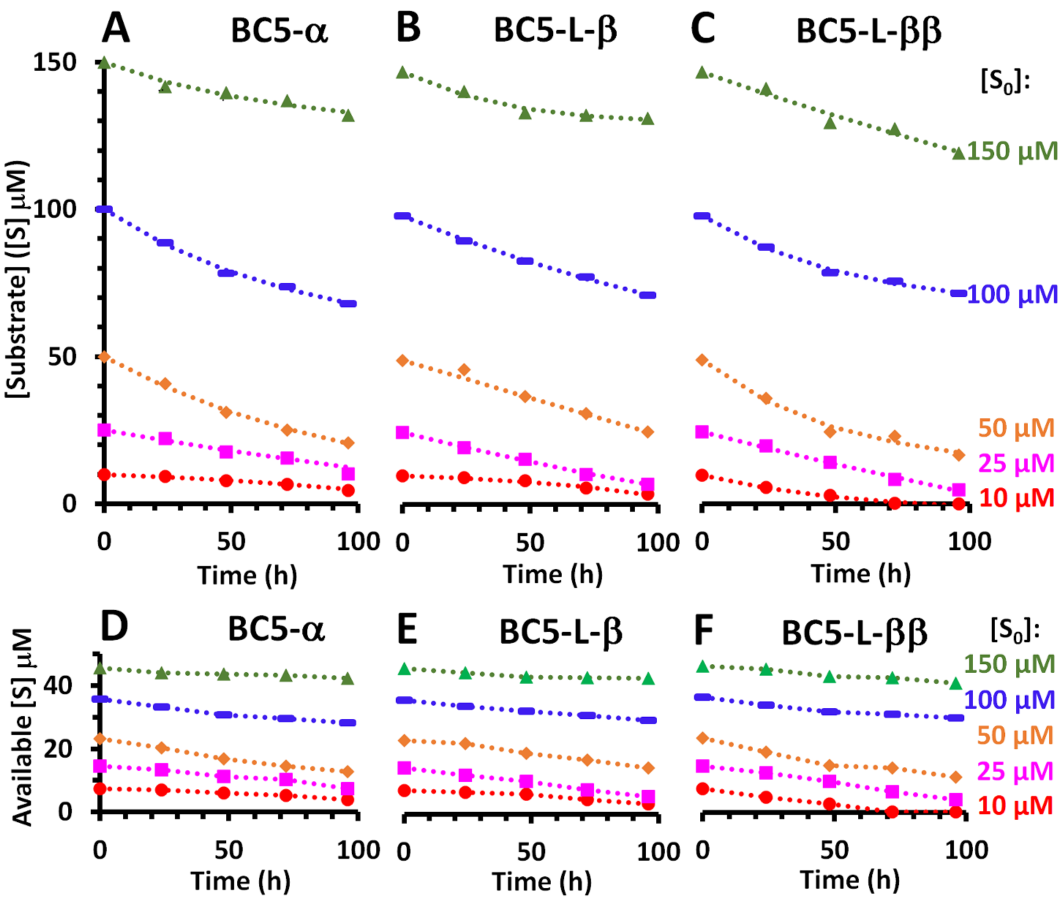

Supplementary Figure 20. Substrate depletion and buffering. **Top row** (**A-C**): Progress curves of the expected depletion of substrate concentration [S] calculated by subtraction of product concentration [P] (obtained from **Figure 7**). **Bottom row** (**D-F**): The progress curve of available substrate [S_a] after consideration of substrate self-association.

This observation was explained by the possibility of self-association of the target substrate and conjugates into stable hairpin structures via intramolecular folding (**Supplementary Figure S19**). Such an explanation considers below the equilibrium dissociation constants (K) of each dominant interaction.

21.1 Model

The binding of free peptidyl-oligonucleotide catalytic conjugate (C) to free target substrate (S) and the formation of a cleavable *substrate-catalyst* complex (CS) may be considered, as a first approximation, in terms of a single-site competition, characterised by their respective self-association (see **Supplementary Figure S19**) and dissociation equilibria, which are dynamic as catalysis reactions progress. In this model (see **Figure 8** in paper), the target substrate associates into a complex (S_c), unavailable for binding to the catalyst, and the catalyst associates into inactive complex (C_c), unable to attack the target substrate.

In order to determine whether such a model, operating alongside Michaelis-Menten kinetics, can simulate the observed velocities, we first considered the fractions (denoted as [concentrations] in square brackets) involved in each self-interaction separately, in isolation from each other, and then in competition according to well-established principles described, for example, in (2, 3).

Some of the total catalyst (C_t) is complexed (C_c), leaving available catalyst (C_a):

$[C]_t = [C]_a + [C]_c$ when $[C]_a = [C]_t - [C]_c$ with equilibrium dissociation $K_c = \frac{[C]_a^2}{[C]_c}$ (1)

substituting into $K_c = \frac{([C]_t - [C]_c)^2}{[C]_c}$ and rearranging $[C]_C = \frac{K_c + 2[C]_t - \sqrt{[K_c + 2[C]_t]^2 - 4 \cdot [C]_t^2}}{2}$ (2)

and, similarly, the total substrate (S_t) and available substrate (S_a):

$[S]_t = [S]_a + [S]_c$ when $[S]_a = [S]_t - [S]_c$ with equilibrium dissociation $K_s = \frac{[S]_a^2}{[S]_c}$ (3)

substituting into $K_s = \frac{([S]_t - [S]_c)^2}{[S]_c}$ and rearranging $[S]_C = \frac{K_s + 2[S]_t - \sqrt{[K_s + 2[S]_t]^2 - 4 \cdot [S]_t^2}}{2}$ (4)

When the available conjugate associates with available substrate into the complex (CS):

$[C]_t = [C] + [CS] + [C]_c$ when from (1) available $[C]_a = [C]_t - [C]_c = [C] + [CS]$ (5)

$[S]_t = [S] + [CS] + [S]_c$ when from (2) available $[S]_a = [S]_t - [S]_c = [S] + [CS]$ (6)

whose equilibrium dissociation $K_{cs} = \frac{[C] \times [S]}{[CS]}$ (7)

Substituting into (7) from (5) $[C] = [C]_a - [CS]$ and from (6) $[S] = [S]_a - [CS]$

$$K_{cs} = \frac{([C]_a - [CS]) \times ([S]_a - [CS])}{[CS]}$$

provides estimate of the expected conjugate complex $[CS]$ at equilibrium with initial substrate, from the observed estimates of equilibrium dissociation K_{cs} and the available substrate $[S]_a$and conjugate $[C]_a$ when rearranged as:

$$[CS] = \frac{([C]_a - [CS]) \times ([S]_a - [CS])}{K_{cs}} = \frac{K_{cs} + [C]_a + [S]_a - \sqrt{[K_{cs} + [C]_a + [S]_a]^2 - 4 \cdot [C]_a \cdot [S]_a}}{2} \quad (8)$$

The target substrate and catalytic conjugate were purified into respective water solutions and lyophilised, where the removal of water into ice crystals before sublimation will concentrate and promote their self-complexations (**Supplementary Figure S19).** With diminished water, and thereby ionic

and hydrogen bonding, hydrophobic bonding within complexes will dominate. When reconstituted back into water and low ionic strength reaction mixtures, the positively-charged peptide in the structure of the catalytic conjugate is also likely to interact with the negatively-charged oligonucleotide (4) to reinforce self-complexations further.

Inactivated complexes of catalytic conjugates are likely to be activated, especially by binding of available substrate, initially to smaller exposed regions, with increase in activation facilitated by rise in substrate concentration. Competitive activation of catalytic conjugate by the substrate available from self-complexation can similarly consider the common conjugate available after their interaction at equilibrium, as above from (2, 3).

21.2 Calculation of fractions expected from this model

The expected target substrate complexed $[S]_c$ was calculated from input of its equilibrium dissociation K_s and the known total substrate concentration $[S]_t$ initially in isolation (eq. 4), when the available substrate $[S]_a$ was obtained by difference (eq. 6). The complexed conjugate was also calculated initially in isolation from an approximated equilibrium dissociation K_{cc} (eq. 2), when the activation of conjugate could be similarly calculated from an initially approximated equilibrium dissociation K_c in competition with the initially-available substrate $[S]_a$ to provide the activated concentration of conjugate available $[C]_a$ for each reaction. Initially approximated values were refined within the non-linear numerical fitting of the model below to simulate the observed data sets of every reaction sample for each conjugate catalyst.

The full binding of substrate and weaker competitive partial binding of substrate to the conjugate (**Figure 8** in paper) were calculated from their input equilibrium dissociations K_{CS} and K_{SS} (respectively), when partial binding of two substrate molecules to a conjugate ("double substrate" complex with K_{ds}) at higher substrate concentrations was also considered. As product formation was observed at each time point sampled, the corresponding depletion of substrate and the effect on the complexation of substrate were calculated to provide the dynamically-available substrate as the reaction progressed. The product bound to the available conjugate in competition with available substrate was also calculated for each time point from its dissociation K_P, as its observed equilibrium concentration dynamically changed as the reaction progressed.

Formation of the cleavable substrate complex $[CS]$ by full binding of the substrate to the different catalytic conjugates has dissociation K_{cs} characteristics reported here and earlier (5). Remaining dissociation characteristics were approximated initially as those expected from the dominant binding. The above calculations were completed alongside Michaelis-Menten kinetics for initial velocities v_0 and initial available substrate $[S]_{a0}$ including the active conjugate $[C]_a$ (in the $V_{max} = [C]_a \times k_{cat}$ term):

$$v_0 = \frac{[C]_a \times k_{cat} \times [S]_{a0}}{K_m + [S]_{a0}} \qquad (11)$$

Non-linear numerical fitting (*via* 'Solver' add-in for Excel) was used to vary initial approximations of k_{cat}, K_m and to refine approximated equilibrium dissociation characteristics (K_c, K_s, K_{ss_S}) by iteratively calculating the resulting expected velocities, until minimised against the observed velocities (*i.e.* minimum sum of squared differences between expected and observed velocities). All of the estimates of fractions of complexation expected were thereby calculated for each reaction time course, which are presented below on a log scale to discern also smaller-scale changes for three conjugate catalysts (**Supplementary Figure S21**).

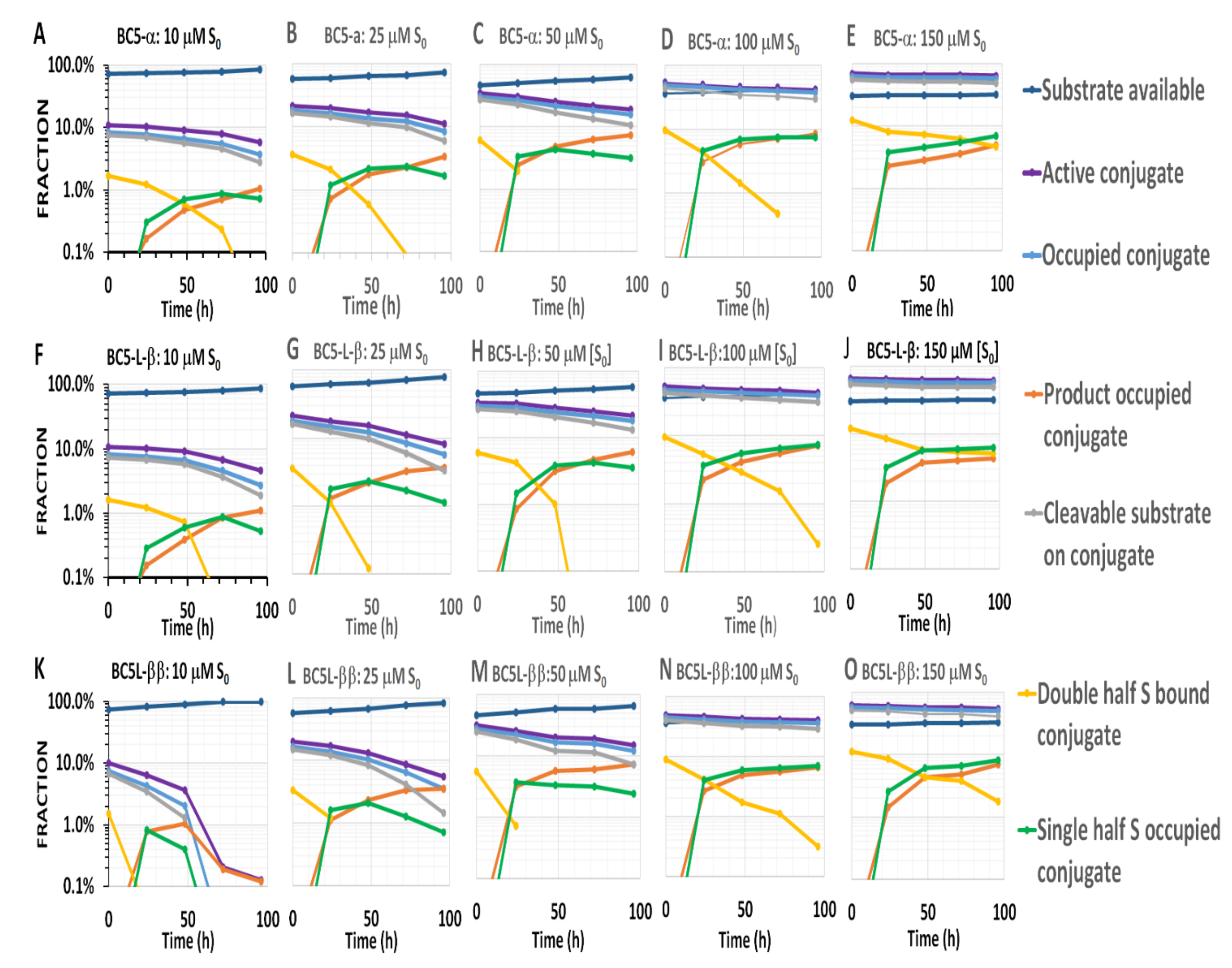

Figure S21. Fractional change in **F**-**Q**-RNA substrate, conjugate and product complexes during the progress of reactions of the catalytic conjugates BC5-α (top row), BC5-L-β (middle row) and BC5-L-ββ (bottom row) for excess initial (total) substrate concentrations (from left to right: $[S]_0$ 10, 25, 50, 100 and 150 µM), as described below in the text.

Substrate available (dark blue trace, **Figure S21**). Despite consumption of substrate into product, in this model, the fraction of ”Substrate available” will not decline to the same extent and remains relatively steady. Such steady levels particularly at higher initial substrate concentrations were related by the replacement of ”Substrate available” from complexed substrate as total substrate was depleted. This buffering of ”Substrate available” manifests as slightly increasing fractional levels of ”Substrate available” as substrate was consumed at lower initial substrate concentrations.

Active conjugate (purple trace, **Figure S21**). Of the total conjugate (5 µM in all cases), only a minor fraction (~10%) was available at small excesses of substrate and fractional levels declined during the course of the reaction, as activating substrate (at small excesses) declined with less buffering from complexed substrate. As the initial substrate concentration is increased, progressively greater proportions of the total conjugate will be activated in this part of the model. However, activated conjugate will still become inactivated at higher substrate concentrations (see below in **Figures S22** and **S23**, not shown in the above **Figure S21**).

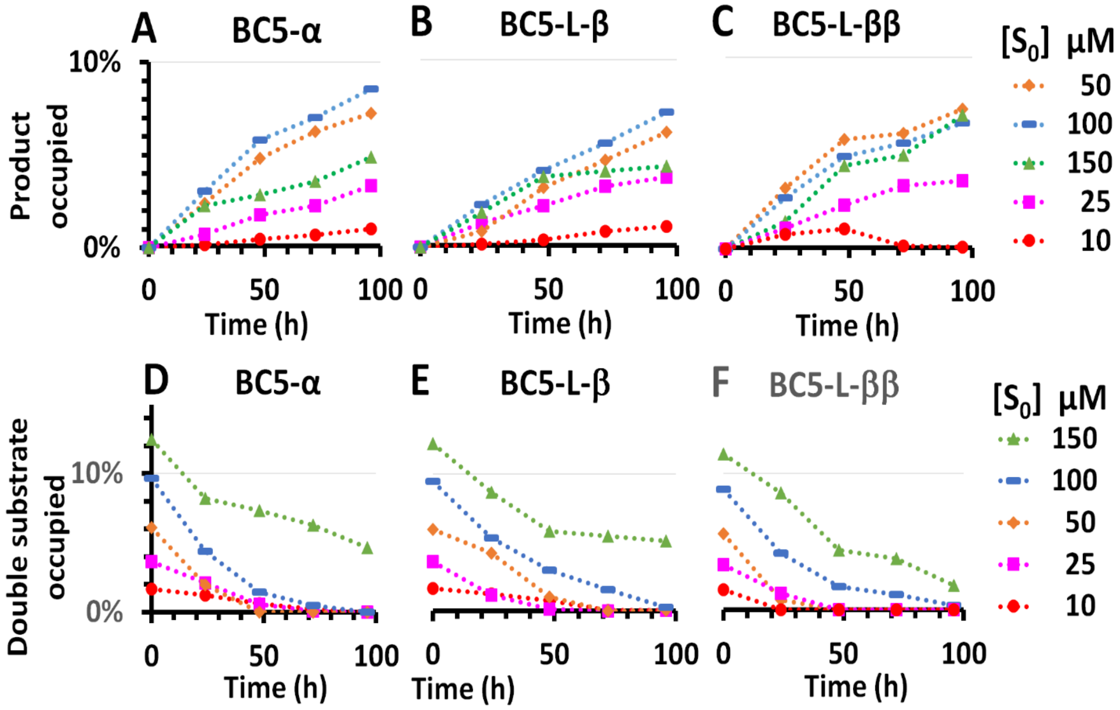

Figure S22. Fractional change in conjugate occupancy with product (top row) and with "double substrate" (bottom row) during the progress of cleavage reactions catalysed by BC5-α, BC5-L-β and BC5-L-ββ conjugate (5 μM), plotted for different initial (total) substrate concentrations of substrate $[S]_0$ present in excess: 10 μM (red-filled circles), 25 μM (pink-filled squares), 50 μM (orange- filled diamonds), 100 μM (blue dashes) and 150 μM (green-filled triangles).

Substrate and product-occupied conjugate. Cleavable substrate dominated the occupancy of the conjugate (grey trace **Figure S21**) due to formation of the "full-size", perfect-match complementary complex. As substrate is cleaved, the occupancy of the conjugate by product will rise but, given ~10× greater equilibrium dissociation constant, there will be less than 10% occupancy (**Figure S22** (**A-C**) and orange-red trace, **Figure S21**). Products of cleaved substrate will have similar dissociation to partial binding of substrate in this model, and cleaved product leaves part of the conjugate unoccupied for the partial binding of substrate (S) (**Figure S21**). Partial binding of substrate ("Single half S occupancy", green trace, **Figure S21**) will compete with and follow the occupancy of product. However, growing with large excesses of substrate, some of the conjugate will become doubly-occupied by a pair of substrate molecules (see **Figure 8** in paper), each partially bound with the other respective unbound portions of the substrates dangling. However, such "double substrate" occupancy of the conjugate (**Figure S22** (**D-F**)) will decline in this model in competition with occupancy of the conjugate by product (**Figure S21** (**A -C**), which increases during catalysis and will leave only a partial binding site for substrate. The rate of decline in double substrate occupancy was greater at lower substrate concentrations than higher substrate concentrations, when "double substrate" occupancy is retained for longer periods of reaction, and remained elevated at the higher substrate concentrations, where the conjugate was also prone to inactivation (**Figure S22** (**D-F**)).

The two dangling ends of the unbound substrate molecules and their two or more dangling positively-charged peptides in the structure of the catalytic conjugate are likely to promote agglomeration and inactivation of the conjugate, which would increase with the greater extent and longer duration of "double substrate" occupancy at higher substrate concentrations (**Figure 8**). The lesser rate of decline of 'double substrate' occupancy with increasing substrate concentrations was therefore used as the key determinant of inactivation in this model, where a power function of the more parabolic nature of agglomeration was estimated as best fit to the rate of "double substrate" occupancy with available substrate concentration.

21.3 Conjugate activation and inactivation model

The above characterisation of activation and inactivation interactions was used as a model to simulate the active conjugate concentration and the inactivated concentration of conjugate, as they vary with the dynamic changes in the available substrate concentration. The underlying equilibrium dissociation characteristics were refined numerically to produce the best fit (*i.e.*, minimum sum of squared residuals) between the expected velocities calculated from this model and the velocities observed experimentally. The resultant sum of this activation and inactivation gave the active conjugate concentration (**Figure S23** (**A-C**)).

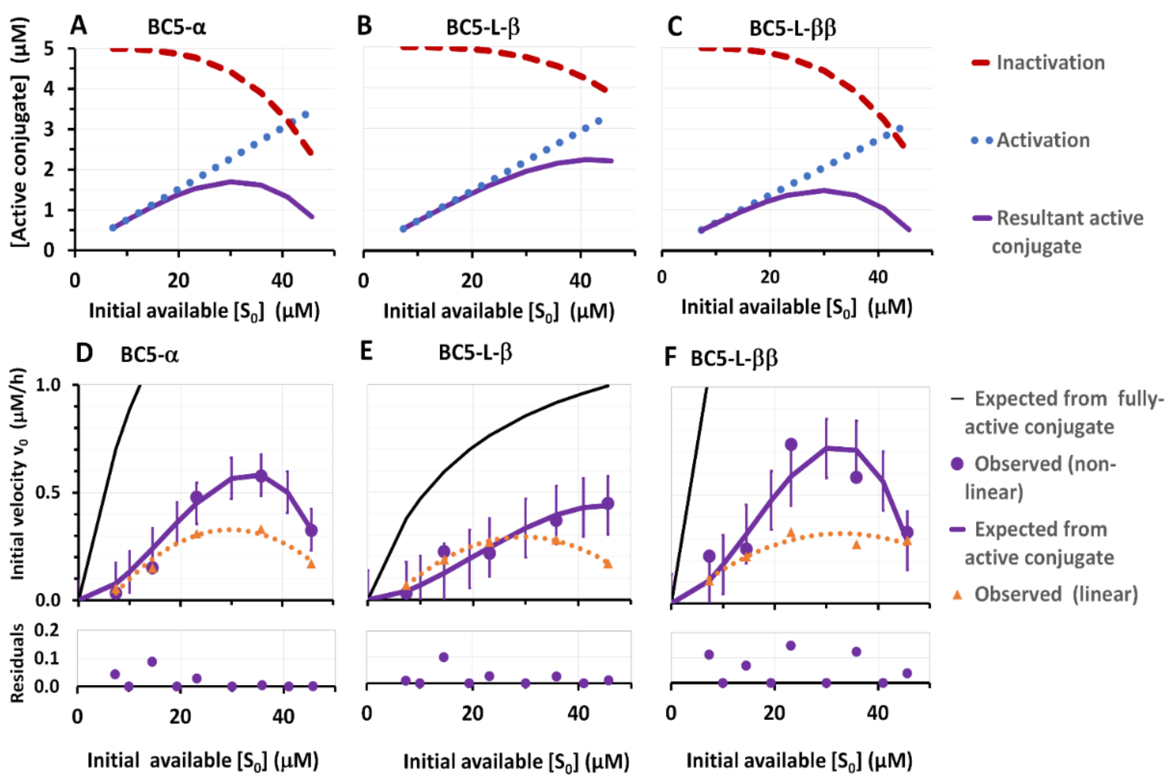

Figure S23. Conjugate activation and inactivation simulation of observed velocities within Michaelis-Menten kinetics. **Top row** compares the model of inactivation (dashed red curves) and activation (dotted blue curves) whose resultants gave the parabolic active conjugate concentrations (continuous purple curves) for three conjugates: (**A**) BC5-α, (**B**) BC5-L-β and (**C**) BC5-L-ββ. **Middle row** compares the initial reaction velocities (v_0) expected from the active conjugate (purple curve) with the observed initial velocities estimated from non-linear fits (purple-filled dots) and from linear fits (orange-filled triangles) of the reaction progress curves: **(D)** BC5-α, **(E)** BC5-L-β and **(F)** BC5-L-ββ. Error bars are standard errors of regression analysis of the observed and expected velocities. **Bottom row** compares the residuals as squared difference between observed and expected.

The model defined by the refined equilibrium dissociation characteristics gave the closest fit to the observed initial reaction velocities from non-linear fitting (**Figure S23** (**D-F**) continuous purple curve). Residuals were raised slightly for '*single*' conjugates at low substrate excesses (**Figure S23**, bottom row), suggestive of another lesser factor here, which had not been accounted for within the modelled complexity.

19.4 Competitive and uncompetitive inhibition

Michaelis-Menten kinetics were considered alone with competitive K_{ic} and uncompetitive K_{iu} inhibitions by product and substrate using Equation 12 (1):

$$v_0 = \frac{[C]_t \times k_{cat} \times [S]_0}{\left(1+{[P]_t}/{K_{ic}}\right)K_m + \left(1+{[P]_t}/{K_{iu}}\right)[S]_0} \quad (12)$$

but there was no possible fit to the observed velocities, particularly when initial velocities were estimated by non-linear fitting. The hyperbolic nature of Michaelis-Menten kinetics alone was unsuited to the peculiar skewed bell-shaped or parabolic kinetics observed, where combination with conjugate activation and inactivation produced a much better fit (**Figure S23**).

Consideration of product inhibition proved more fruitful when combined within the conjugate activation and inactivation model. Product inhibition was considered by the increases in product concentrations during the progress of each of the catalytic reactions, where velocities were approximated from the initial velocity v_0 and non-linearity factor η as described by using Equation 13 (1):

$$v = \frac{d[P]}{dt} = v_0 - \eta[P] \quad (13)$$

The velocities estimated for the sampled points during reaction progress were then compared to expected velocities calculated from using Equation 14 (1):

$$v_{exp} = \frac{[C]_a \times k_{cat} \times [S]_a}{\left(1+{[P]_t}/{K_{ic}}\right)K_m + \left(1+{[P]_t}/{K_{iu}}\right)[S]_a} \quad (14)$$

where the active conjugate $[C]_a$ was used together with the changes in available substrate $[S]_a$ and product $[P]_t$ concentrations during the time (t) course of the reactions (**Figure S24**). Although the available substrate declined less during the course of the reactions than the total substrate, at lower substrate concentrations, there was a greater decline in available substrate as product was produced.

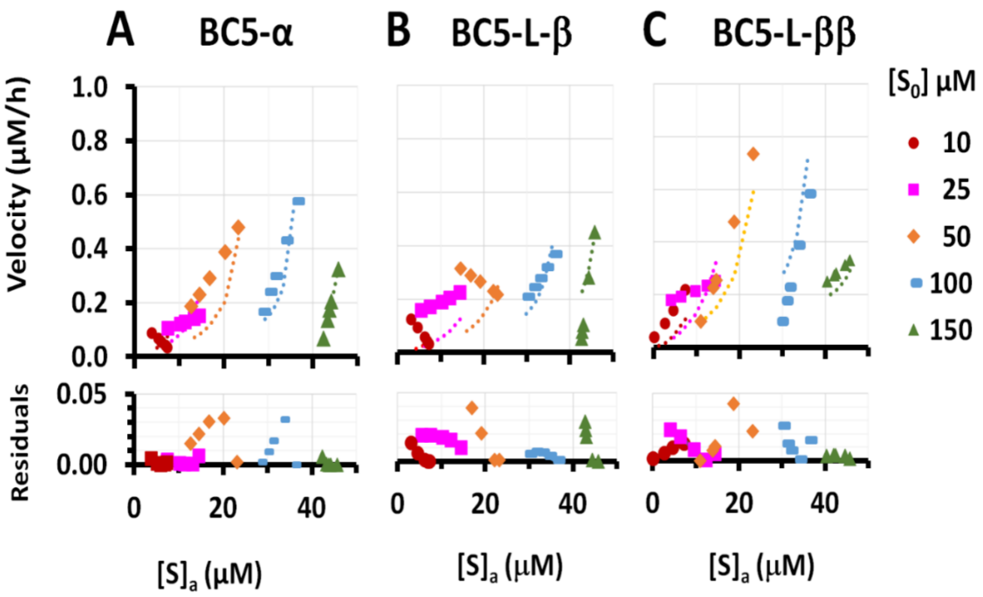

Figure S24. Uncompetitive product inhibition for conjugates BC5-α **(A)**, BC5-L-β **(B)** and BC5-L-ββ **(C)**. Velocities were estimated from the non-linear fit of reaction progress curves (filled symbols for different initial substrate concentrations [S_0]) and compared to velocities expected (dotted traces) from the model simulation of active conjugate and available substrate for the estimates of uncompetitive product inhibition (**Table S2**).

At low substrate concentrations, the reaction velocities increased during reaction progress for the conjugate catalysts with a single peptide (BC5-α, BC5-L-β), but velocities declined for the *"bis"* conjugate with two catalytic peptides (BC5-L-ββ). Although the velocities expected from Michaelis-Menten kinetics were in the same region, they did not show increases during reaction progress (**Figure S24** red and pink traces). However, at higher substrate concentrations, available substrate buffering maintained less change in available substrate during the time course (**Figure S21**). For these higher

substrate concentrations, the velocities expected from the model of complex interactions around Michaelis-Menten kinetics, with product inhibition included in the model, approached those estimated from the reaction progress curves with relatively small residuals (**Figure S24**).

Attempts were made to determine any competitive product inhibition using non-linear fitting by minimising residuals (sum of squared differences between estimated and expected velocities), but competitive inhibition was consistently insignificant with large K_{ic} values (>>100,000). However, uncompetitive product inhibition had consistently significant values in the region of 18-21 μM, a little less than the K_m values 20-23 μM (**Table S2**). Similar values may be expected as the same oligonucleotide binding sequence is used in all cases, with changes in the way the catalytic peptide is attached and number of peptides being the main variable.

Table S2. Kinetic characteristics input and output from the model described. The equilibrium dissociation constants for the cleavable substrate complex K_{cs} was input from the reciprocal of the equilibrium association constant estimated experimentally. The binding affinities of cleaved product and half substrate were assumed to be similar and input at the same value. All other kinetic characteristics were estimated by non-linear fitting.

			**BC5-α**	**BC5-L-β**	**BC5-L-ββ**
**Michaelis-Menten**	K_m	(μM)	23.81	20.65	20.69
**Catalytic turnover**	K_{cat}	(/h)	0.60	0.29	0.82
**Maximum velocity**	V_{max}	(μM/h)	2.99	1.44	4.10
**Uncompetitive inhibition**	K_{iu}	(μM)	20.73	20.98	18.71
**Competitive inhibition**	K_{ic}	(μM)	>100,000	>100,000	>100,000
**Conjugate complex**	K_{cc}	(μM)	22.10	21.91	21.49
**Conjugate activation**	K_c	(μM)	21.61	22.31	23.95
**Substrate complex**	K_{cs}	(μM)	2.00	2.00	2.00
**Half substrate complex**	K_{ss}	(μM)	20.00	20.00	20.00
**Product complex**	K_p	(μM)	20.00	20.00	20.00

The principal difference in kinetics arising from the structural differences in the conjugate catalysts was in V_{max} or the catalytic turnover k_{cat}. As may be expected, the conjugates with a pair of catalytic peptides had much higher reaction turnover (0.8 per hour), whereas, for single catalytic peptides, the α anomer produced the highest turnover (0.6 per hour), double that of the elongated β anomer (0.29 per hour).

22. References for the Supporting Information

1. Cao,W. and De La Cruz,E.M. (2013) Quantitative full time course analysis of nonlinear enzyme cycling kinetics. *Sci. Rep.*, **3**, 2658.

2. Bielec,K., Sozanski,K., Seynen,M., Dziekan,Z., ten Wolde,P.R. and Holyst,R. (2019) Kinetics and equilibrium constants of oligonucleotides at low concentrations. Hybridization and melting study. *Phys. Chem. Chem. Phys.*, **21**, 10798–10807.

3. Qi,X., Loiseau,F., Chan,W.L., Yan,Y., Wei,Z., Milroy,L.-G., Myers,R.M., Ley,S. V, Read,R.J., Carrell,R.W., et al. (2011) Allosteric modulation of hormone release from thyroxine and corticosteroid-binding globulins. *J. Biol. Chem.*, **286**, 16163–16173.

4. Gebrezgiabher,M., Zalloum,W.A., Clarke,D.J., Miles,S.M., Fedorova,A.A., Zenkova,M.A. and Bichenkova,E. V (2020) RNA knockdown by synthetic peptidyl-oligonucleotide ribonucleases: behavior of recognition and cleavage elements under physiological conditions. *J. Biomol. Struct. Dyn.*, 1–20.

5. Staroseletz,Y., Amirloo,B., Williams,A., Lomzov,A., Burusco,K.K., Clarke,D.J., Brown,T., Zenkova, M.A., Bichenkova,E.V. (2020) Strict conformational demands of RNA cleavage in bulge-loops created by peptidyl-oligonucleotide conjugates. *Nucl. Acids Res.*, **48**, 10662-10679.
